# Supplementary material for: Identifying the prevalence and correlates of multimorbidity in middle-aged men and women: a cross-sectional population-based study in four African countries
Source: BMJ Open. 2023 Mar 14;13(3):e067788. doi: 10.1136/bmjopen-2022-067788 (PMC10016250; doi:10.1136/bmjopen-2022-067788)
Supplement: Supplementary data [file bmjopen-2022-067788supp002.pdf]

Supplementary Table 1

	Agincourt, SA			Dikgale, SA			Soweto, SA		
	Men (n=573)	Women (n=892)	Total (n=1465)	Men (n=356)	Women (n=812)	Total (n=1168)	Men (n=1025)	Women (n=1002)	Total (n=2027)
**Age (yrs)**	51 (45-56)	51 (46-56)	51 (46-56)	50 (45-55)	51 (46-56)	51 (45-55)	49 (44-55)	49 (44-54)	49 (44-54)
**Highest level of education attained**									
No formal	123 (21.5)	280 (31.4)	403 (27.5)*	22 (6.2)	74 (9.1)	96 (8.2)	8 (0.8)	2 (0.3)	10 (0.6)*
Primary	235 (41.1)	340 (38.1)	575 (39.3)	113 (31.7)	273 (33.7)	386 (33.1)	117 (11.4)	636 (80.9)	753 (41.6)
Secondary	175 (30.6)	223 (25.0)	398 (27.2)	204 (57.3)	440 (54.2)	644 (55.2)	749 (73.0)	147 (18.7)	895 (49.4)
Tertiary	39 (6.8)	49 (5.5)	88 (6.0)	17 (4.8)	24 (3.0)	41 (3.5)	152 (14.8)	1 (0.1)	153 (8.4)
**Employment**									
Unemployed	326 (62.3)	558 (64.8)	884 (63.9)	193 (54.7)	532 (65.6)	725 (62.3)*	355 (34.6)	455 (45.4)	810 (40.0)*
Employed	197 (37.7)	303 (35.2)	500 (36.1)	160 (45.3)	279 (34.4)	439 (37.7)	670 (65.4)	547 (54.6)	1217 (60.0)
**Partnership status**									
Never married or co-habited	75 (13.1)	59 (6.3)	134 (9.2)*	103 (28.9)	185 (22.8)	288 (24.7)	265 (25.9)	51 (7.7)	316 (18.7)*
Married or co-habiting	445 (77.1)	537 (60.2)	982 (67.0)	178 (50.0	427 (52.6)	605 (51.8)	570 (55.7)	266 (40.1)	836 (49.5)
Divorced, separated, partner deceased	53 (9.3)	296 (33.2)	349 (23.8)	75 (21.1)	200 (24.6)	275 (23.5)	189 (18.5)	347 (52.3)	536 (31.8)
**SES site quintile**	3 (2-4)	4 (2-5)	3 (2-5)*	3 (2-4)	4 (2-5)	4 (2-5)*	4 (3-5)	2 (2-3)	3 (2-5)*
**BMI (kg/m^2)**	23 (20-27)	28.6 (24-33)	26 (22-31) *	20.6 (19-24)	30.1 (25-36)	26.9 (21-33)*	24.2 (20-28)	32.9 (29-38)	28.4 (23-34)*
**Smoking**									
Never smoker	291 (51.0)	882 (98.8)	1173(80.1)*	54 (15.2)	757 (93.3)	811 (69.6)*	311 (30.4)	893 (89.9)	1204(59.7)*
Current smoker	155 (27.1)	3 (0.3)	158 (10.8)	225 (63.4)	25 (3.1)	250 (21.4)	540 (52.8)	49 (4.9)	589 (29.2)

	Agincourt, SA			Dikgale, SA			Soweto, SA		
	Men (n=573)	Women (n=892)	Total (n=1465)	Men (n=356)	Women (n=812)	Total (n=1168)	Men (n=1025)	Women (n=1002)	Total (n=2027)
Previous smoker	125 (21.9)	7 (0.8)	132 (9.0)	76 (21.4)	29 (3.6)	105 (9.0)	172 (16.8)	51 (5.1)	223 (11.1)
**Alcohol status**									
Never consumed alcohol	187 (32.6)	731 (82.0)	918 (62.7)*	55 (15.5)	561 (69.3)	616 (52.9)*			
Current non-problematic consumer	218 (38.1)	52 (5.8)	270 (18.4)	77 (21.6)	45 (5.6)	122 (10.5)	726 (100.0)		726 (100.0)
Current problematic consumer	18 (3.1)	2 (0.2)	20 (1.4)	142 (39.9)	57 (7.1)	199 (17.1)			
Former consumer	150 (26.2)	107 (12.0)	257 (17.5)	82 (23.0)	146 (18.0)	228 (19.9)			
**GPAQ MVPA (mins/wk)/100**	7.2 (2-14.4)	6 (1.8-13.8)	6.3 (1.8-14.1)	11.4 (6.6-25.8)	12.6 (7.2-21)	12.1 (7.2-21.9)	6.6 (2.1-17.4)	2.1 (0.6-8.6)	1.2 (4.15-13.8)*

Continuous data presented as median (IQR) and categorical data as n(%); * = p<0.05 for difference between men and women in respective sites.

Supplementary Table 2

	Nairobi, Kenya			Nanoro, Burkina Faso			Navrongo, Ghana		
	Men (n=886)	Women (n=1056)	Total (n=1942)	Men (n=1045)	Women (n=1039)	Total (n=2084)	Men (n=923)	Women (n=1091)	Total (n=2014)
**Age (yrs)**	48 (44-53)	48 (44-52)	48 (44-53)	50 (44-55)	50 (45-54)	50 (45-55)	50 (46-55)	52 (47-56)	51 (46-56)*
**Highest level of education attained (%)**									
No formal	34 (3.8)	113 (10.7)	147 (7.6)*	758 (72.8)	960 (93.2)	1718 (83.0)*	570 (61.9)	843 (77.6)	1413 (70.4)*
Primary	447 (50.5)	663 (62.8)	1110 (57.2)	181 (17.4)	58 (5.6)	239 (11.5)	206 (22.4)	177 (16.3)	383 (19.1)
Secondary	383 (43.2)	276 (26.1)	659 (34.0)	86 (8.3)	10 (1.0)	96 (4.6)	118 (12.8)	57 (5.3)	175 (8.7)
Tertiary	22 (2.5)	4 (0.4)	26 (1.3)	16 (1.5)	2 (0.2)	18 (0.9)	27 (2.9)	9 (0.8)	36 (1.8)
**Employment (%)**									
Unemployed	23 (2.6)	90 (8.5)	113 (5.8)*	16 (1.5)	5 (.5)	21 (1.0)*	321 (34.9)	429 (39.4)	750 (37.4)*
Employed	860 (97.4)	966 (91.8)	1826 (94.2)	1026 (98.5)	1030 (99.5)	2056 (99.0)	599 (65.1)	659 (60.6)	1258 (62.6)
**Partnership status (%)**									
Never married or co-habited	13 (1.5)	70 (6.6)	83 (4.3)*	14 (1.3)	3 (0.3)	17* (0.8)	15 (1.6)	5 (0.5)	20* (1.0)
Married or co-habiting	808 (91.2)	486 (46.1)	1,294 (66.7)	1021 (97.9)	794 (76.7)	1815 (87.3)	787 (85.4)	694 (63.6)	1481 (73.6)
Divorced, separated, partner deceased	65 (7.3)	499 (47.3)	564 (29.1)	8 (0.8)	238 (23.0)	246 (11.8)	120 (13.0)	392 (35.9)	512 (25.4)
**SES site quintile (median)**	3 (2-5)	3 (2-4)	3 (2-4)*	3 (2-5)	3 (2-4)	3 (2-5)*	3 (2-5)	3 (2-4)	3 (2-4)*
**BMI (median)**	22.2 (20-25)	26.9 (23-32)	24.4 (21-29)*	21.1 (19-23)	19.8 (18-22)	20.4 (19-23)*	20.6 (19-22)	21.4 (20-24)	21 (19-23)*
**Smoking (%)**									
Never smoker	418 (47.2)	975 (92.3)	1393 (71.8)*	779 (74.7)	1033 (99.8)	1812 (87.2)*	332 (36.0)	1052 (96.6)	1384 (68.8)*
Current smoker	208 (23.5)	27 (2.6)	235 (12.1)	142 (13.6)	0.0 (0.0)	142 (6.8)	388 (42.0)	21 (1.9)	409 (20.3)

	Nairobi, Kenya			Nanoro, Burkina Faso			Navrongo, Ghana		
	Men (n=886)	Women (n=1056)	Total (n=1942)	Men (n=1045)	Women (n=1039)	Total (n=2084)	Men (n=923)	Women (n=1091)	Total (n=2014)
Previous smoker	259 (29.3)	54 (5.1)	313 (16.1)	122 (11.7)	2 (0.2)	124 (6.0)	203 (22.0)	16 (1.5)	219 (10.9)
**Alcohol status (%)**									
Never consumed alcohol	257 (29.0)	758 (71.8)	1015 (52.3)*	273 (26.2)	277 (26.7)	550 (26.5)*	71 (7.7)	231 (21.2)	302 (15.0)*
Current non-problematic consumer	174 (19.6)	32 (3.0)	206 (10.6)	544 (52.3)	520 (50.2)	1064 (51.2)	252 (27.4)	426 (39.1)	678 (33.7)
Current problematic consumer	126 (14.2)	30 (2.8)	156 (8.0)	160 (15.4)	100 (9.67	260 (12.5)	461 (50.0)	167 (15.3)	628 (31.3)
Former consumer	329(37.1)	236 (22.4)	565 (29.1)	64 (6.1)	139 (13.4)	203 (9.8)	137 (14.9)	265 (24.3)	402 (20.0)
**GPAQ MVPA (mins/wk)/100**	12.6 (5.4-31.8)	8.4 (3.9-21.6)	10.5 (4.2-26)*	21.6 (1.2-30.6)	29.4 (5.7-36)	25.2 (3.6-33.6)*	24 (9-30.6)	16.2 (3-26.1)	19.8 (4.5-28.8)*

Continuous data presented as median (IQR) and categorical data as n(%); * = p<0.05 for difference between men and women in respective sites.

Supplementary Table 3

	Agincourt: SA			Dikgale: SA			Soweto, SA		
	Men (n=573)	Women (n=892)	Total (n=1465)	Men (n=356)	Women (n=812)	Total (n=1168)	Men (n=1025)	Women (n=1002)	Total (n=2027)
HIV	190 (33.2)	315 (35.3)	505 (34.5)	73 (19.0)	178 (22.2)	251 (21.9)	198 (20.5)	121 (20.5)	319 (21.1)
Asthma	65 (12.8)	58 (8.0)	123 (10.0)	22 (6.6)	33 (4.2)	55 (4.9)	36 (3.6)		36 (3.6)
Diabetes	55 (9.6)	84 (9.4)	139 (9.5)	33 (9.3)	105 (12.9)	138 (11.8)	89 (8.7)	190 (19.0)	279 (13.8)
Chronic kidney disease	76 (13.9)	145 (17.4)	221 (16.0)	30 (9.4)	113 (15.5)	143 (13.6)	129 (13.4)	-	129 (13.4)
Dyslipidaemia	334 (58.3)	740 (83.0)	1074 (73.3)	208 (58.4)	710 (87.4)	918 (78.6)	639 (62.3)	772 (77.1)	1,411 (69.6)
Hypertension	248 (43.3)	513 (57.5)	761 (52.0)	114 (43.2)	391 (48.1)	505 (43.2)	539 (52.6)	540 (53.9)	1079 (53.2)
Cardiovascular disease	22 (3.8)	46 (5.2)	68 (4.6)	21 (5.9)	59 (7.3)	80 (6.9)	36 (3.5)		36 (3.5)
Number of conditions									
0	62 (10.8)	38 (4.3)	100 (6.8) *	72 (20.2)	36 (4.7)	108 (9.3) *	131 (12.8)	71 (7.1)	202 (10.0) *
1	196 (34.2)	193 (21.6)	389 (26.5)	133 (37.4)	243 (29.9)	376 (32.2)	351 (34.2)	368 (36.7)	719 (35.5)
2	192 (33.5)	354 (39.7)	546 (37.3)	102 (28.6)	323 (39.8)	425 (36.4)	358 (34.9)	440 (43.9)	798 (39.4)
3	89 (15.5)	236 (26.5)	325 (22.2)	36 (10.1)	148 (18.2)	184 (15.8)	143 (14.0)	117 (11.7)	260 (12.8)
4	27 (4.7)	63 (7.1)	90 (6.1)	9 (2.5)	55 (6.8)	64 (5.5)	40 (3.9)	6 (0.6)	46 (2.3)
5	7 (1.2)	8 (0.9)	15 (1.0)	4 (1.1)	6 (0.7)	10 (0.9)	2 (0.2)	-	2 (0.1)
6	0	0	0	0	1 (0.1)	1 (0.1)	0	-	0
Multimorbidity	315 (54.9)	661 (74.1) *	976 (66.6)	151 (42.4)	533 (65.5) *	684 (58.6)	543 (53)	563 (56.2) *	1106 (54.6)

Data presented as n(%); * = p<0.05 for difference between men and women in respective sites

Supplementary Table 4

	Nairobi, Kenya			Nanoro, Burkina Faso			Navrongo, Ghana		
	Men (n=886)	Women (n=1056)	Total (n=1942)	Men (n=1045)	Women (n=1039)	Total (n=2084)	Men (n=923)	Women (n=1091)	Total (n=2014)
HIV	67 (8.9)	172 (17.7)	239 (13.8)	5 (0.5)	4 (0.4)	9 (0.4)	9 (1.0)	7 (0.6)	16 (0.8)
Asthma	20 (2.3)	41 (4.0)	61 (3.2)	19 (2.1)	23 (2.2)	42 (2.2)	3 (0.3)	8 (0.7)	11 (0.5)
Diabetes	44 (5.0)	104 (9.2)	148 (7.6)	65 (6.2)	32 (3.1)	97 (4.6)	42 (4.6)	48 (4.4)	90 (4.5)
Chronic kidney disease	94 (11.5)	138 (14.4)	232 (13.1)	73 (7.4)	81 (7.9)	154 (7.7)	78 (9.0)	87 (8.5)	165 (8.7)
Dyslipidaemia	631 (71.2)	909 (86.1)	1540(79.3)	643 (61.5)	889 (85.6)	1,532 (73.5)	394 (42.7)	861 (78.9)	1,255 (62.3)
Hypertension	199 (22.5)	314 (29.7)	513 (26.4)	211 (20.2)	124 (11.9)	335 (16.1)	223 (24.2)	265 (24.3)	488 (24.2)
Cardiovascular disease	28 (3.2)	55 (5.2)	83 (4.3)	15 (1.4)	15 (1.4)	31 (1.5)	23 (2.5)	28 (2.6)	51 (2.5)
Number of conditions									
0	156 (17.6)	64 (6.1)	220 (11.3) *	295 (28.2)	113 (10.9)	408 (19.6) *	345 (37.4)	147 (13.5)	492 (24.4) *
1	453 (51.1)	481 (45.6)	934 (48.1)	518 (49.6)	716 (68.9)	1234 (59.2)	412 (44.6)	640 (58.7)	1052 (52.2)
2	209 (23.6)	336 (31.8)	545 (28.1)	188 (18.0)	183 (17.6)	371 (17.8)	139 (15.1)	252 (23.1)	391 (19.4)
3	60 (6.8)	130 (12.3)	190 (9.8)	39 (3.7)	22 (2.1)	61 (2.9)	26 (2.8)	49 (4.5)	75 (3.7)
4	8 (0.9)	38 (3.6)	16 (0.8)	5 (0.5)	4 (0.4)	9 (0.4)	1 (0.1)	2 (0.2)	3 (0.1)
5	0	4 (0.4)	4 (0.2)	0	1 (0.1)	1 (0.1)	0	1 (0.1)	1 (0.1)
6	0	3 (0.3)	3 (0.2)	0	0	0	0	0	0
Multimorbidity	277 (31.3)	511 (48.4) *	758 (39)	232 (22.2)	210 (20.2) *	442 (21.2)	166 (18)	304 (27.9) *	470 (23.3)

Data presented as n(%); * = p<0.05 for difference between men and women in respective site
