# Supplementary material for: Identifying the prevalence and correlates of multimorbidity in middle-aged men and women: a cross-sectional population-based study in four African countries
Source: BMJ Open. 2023 Mar 14;13(3):e067788. doi: 10.1136/bmjopen-2022-067788 (PMC10016250; doi:10.1136/bmjopen-2022-067788)

Supplementary figure 1

a

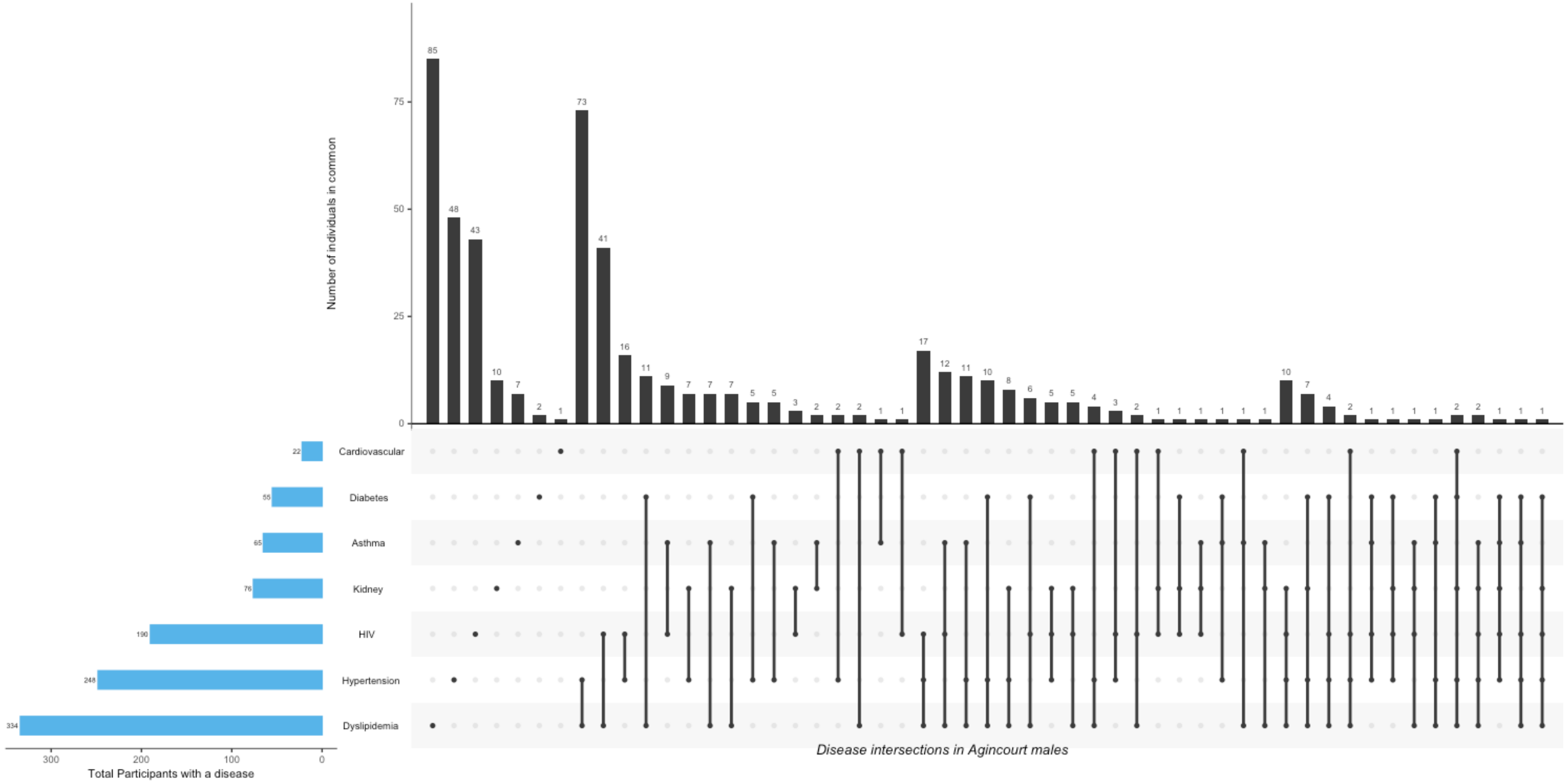

Supplementary figure 2

b

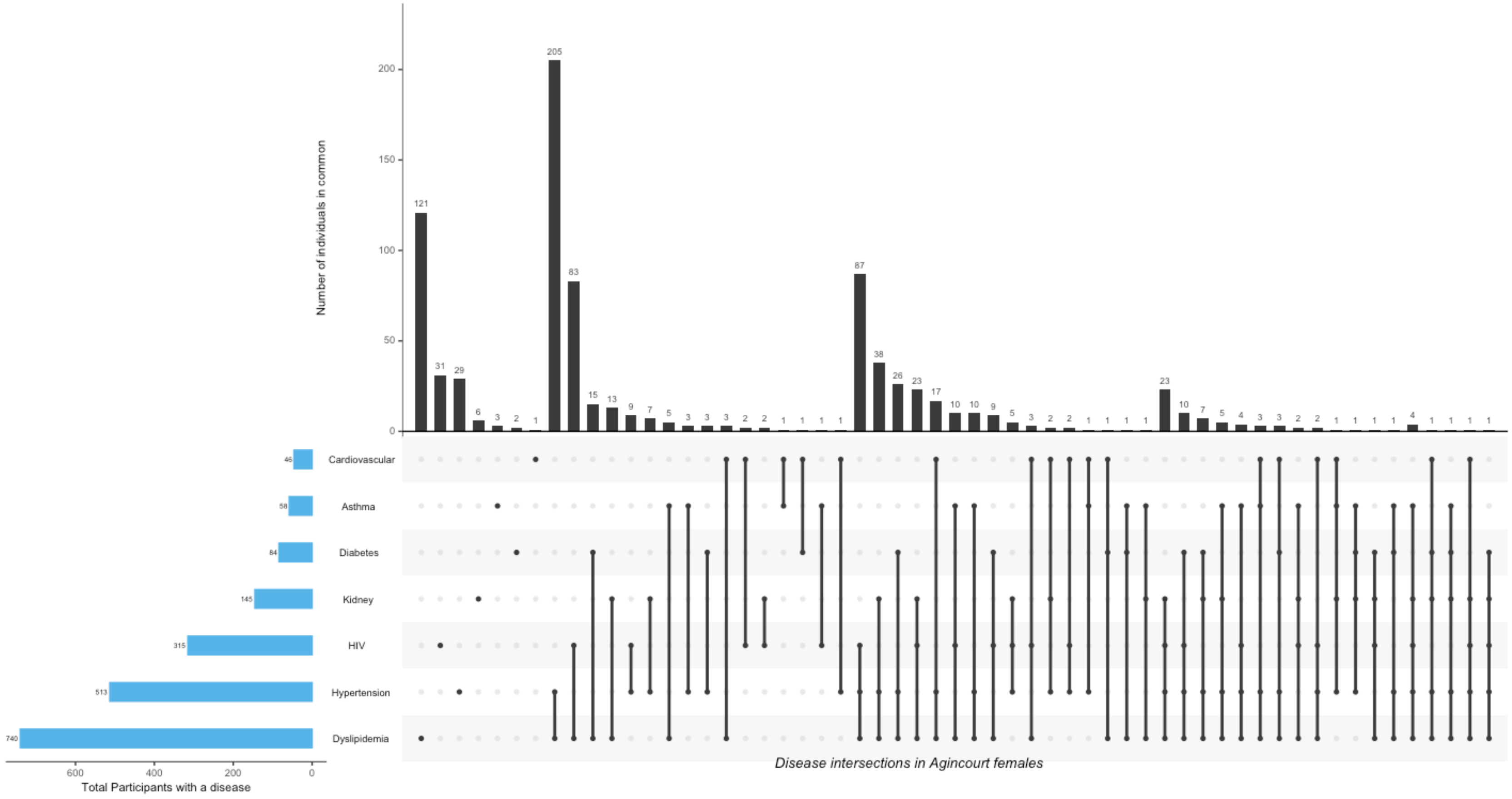

Supplementary figure 3

c

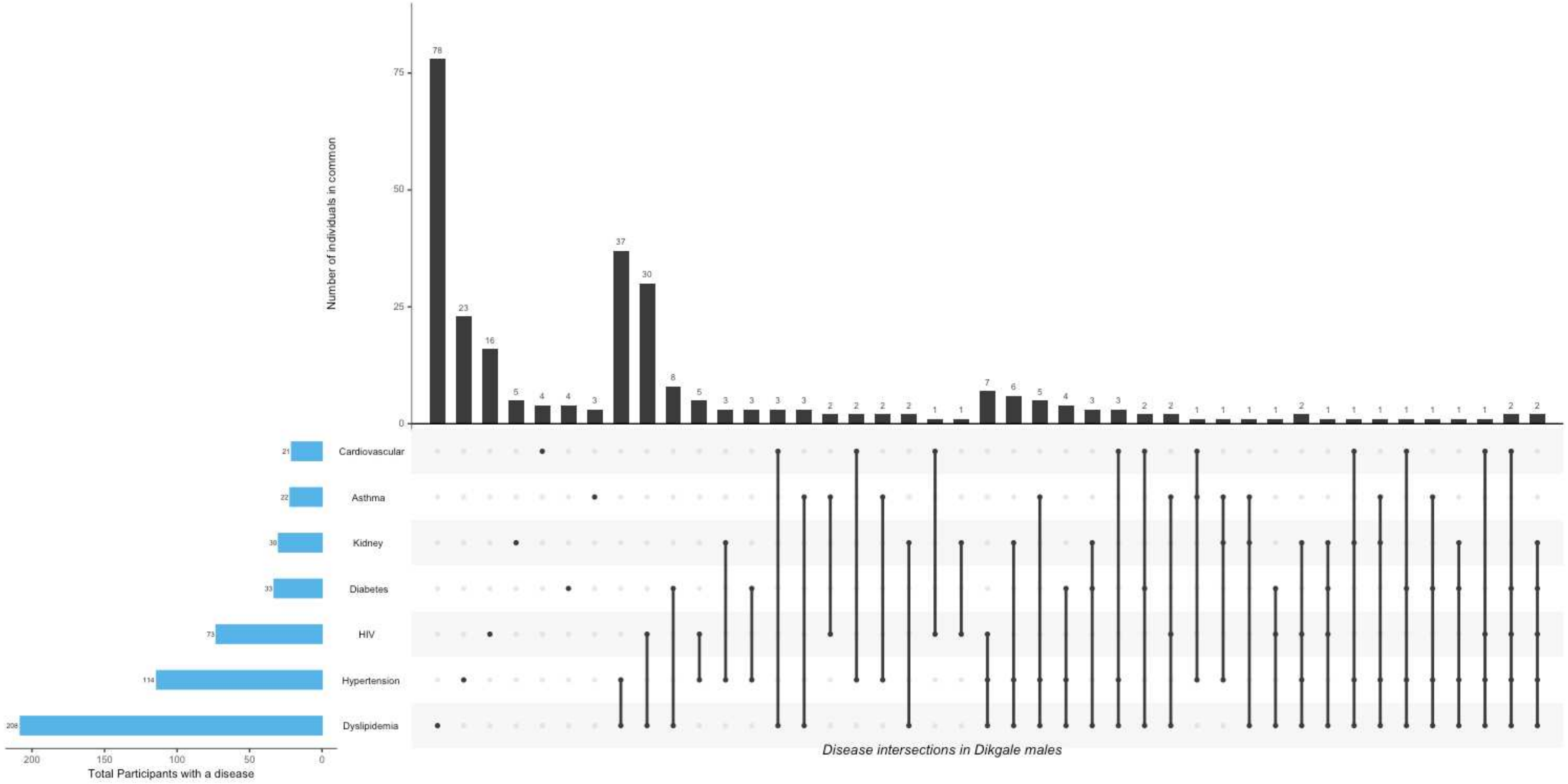

Supplementary figure 4

d

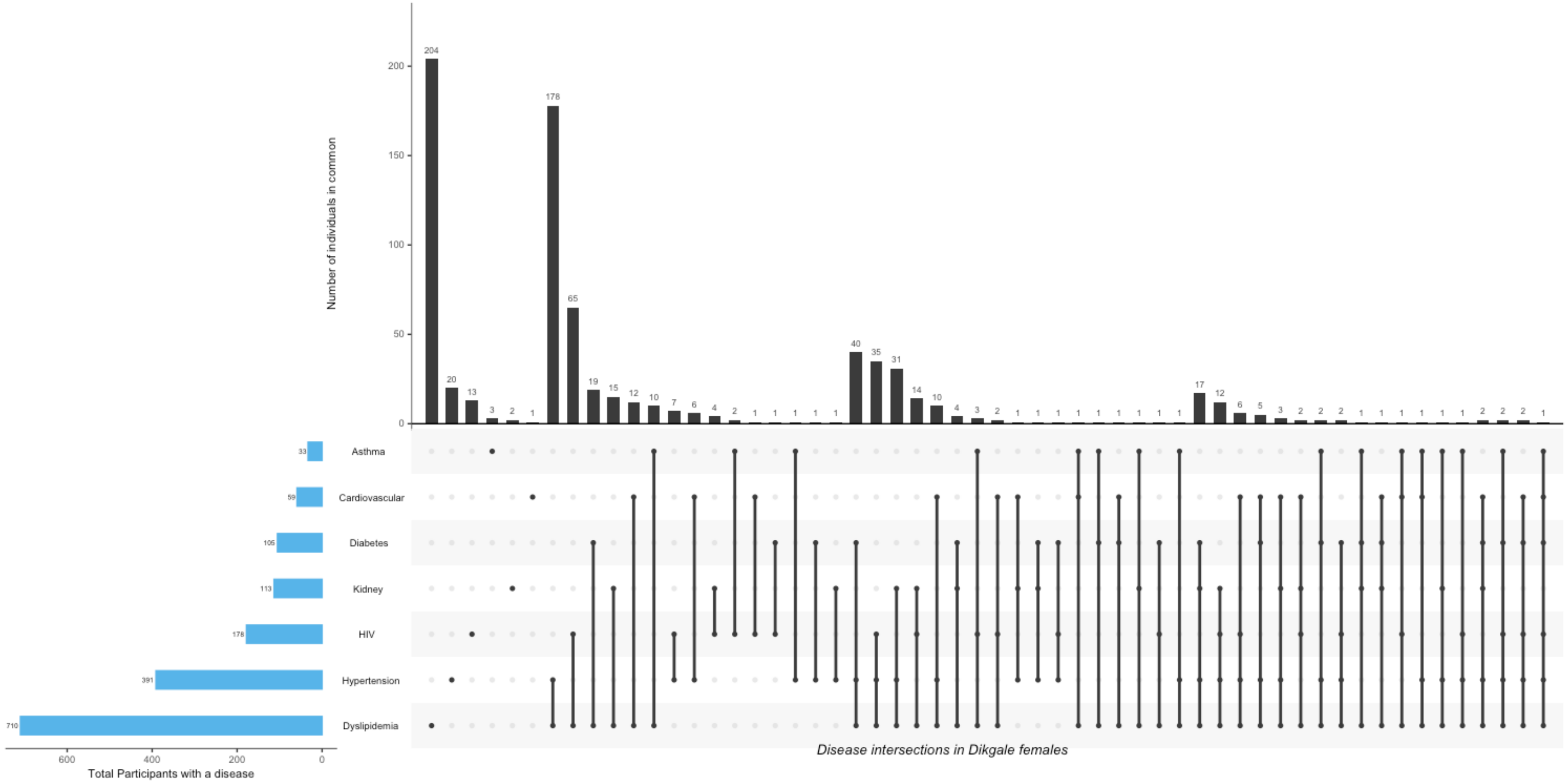

Supplementary figure 5 e

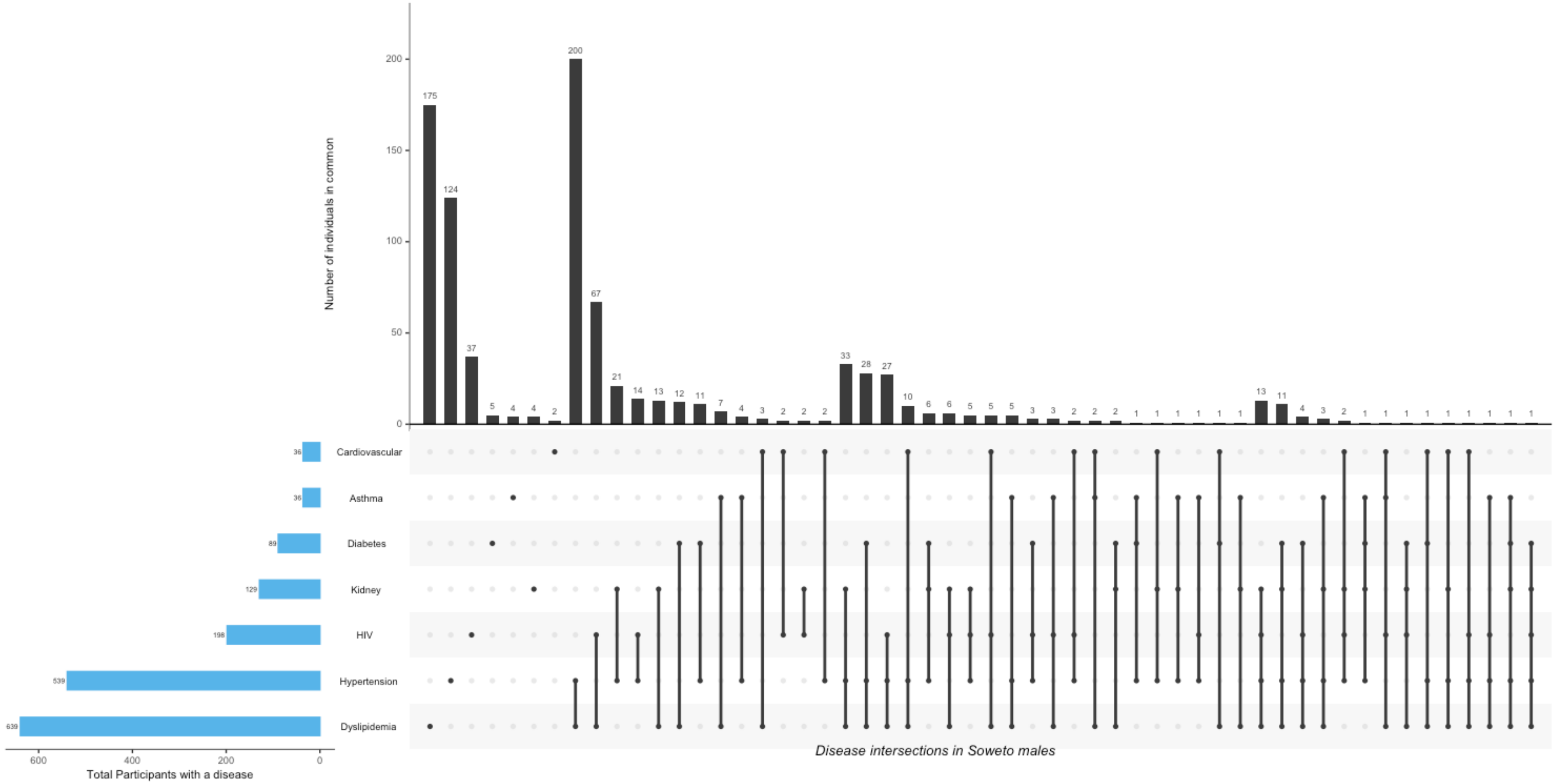

Supplementary figure 6

f

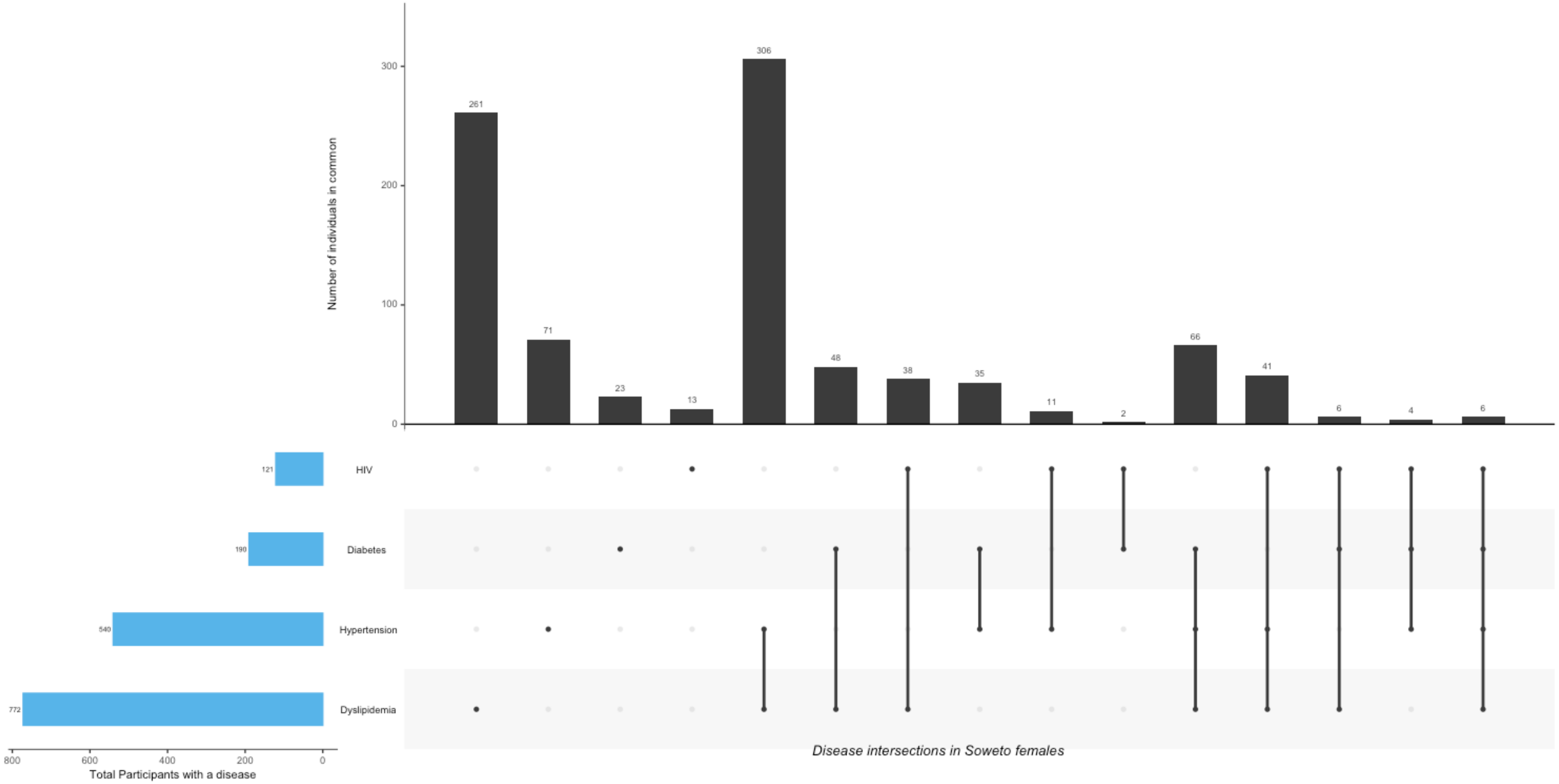

Supplementary figure 7

g

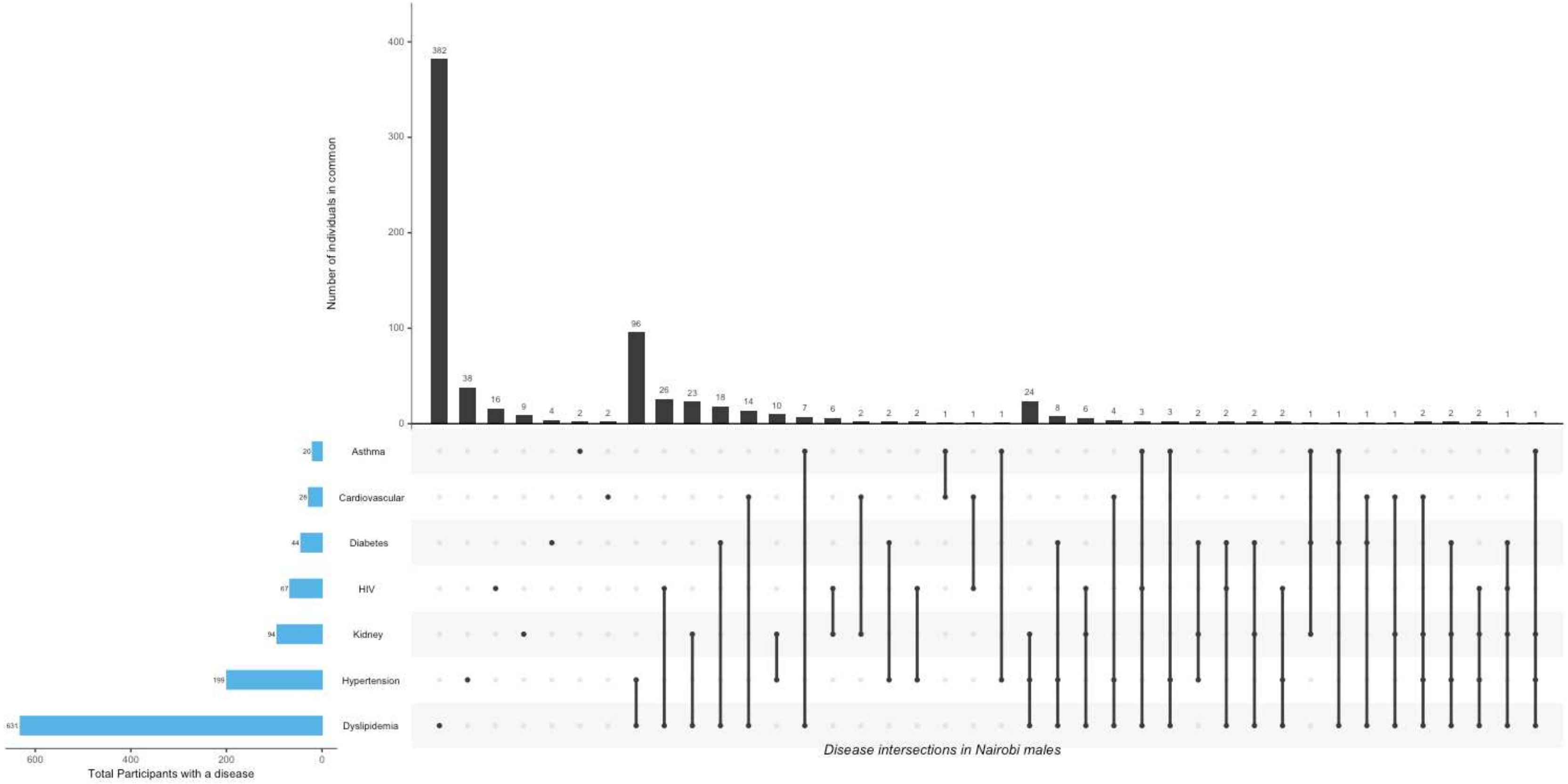

Supplementary figure 8

h

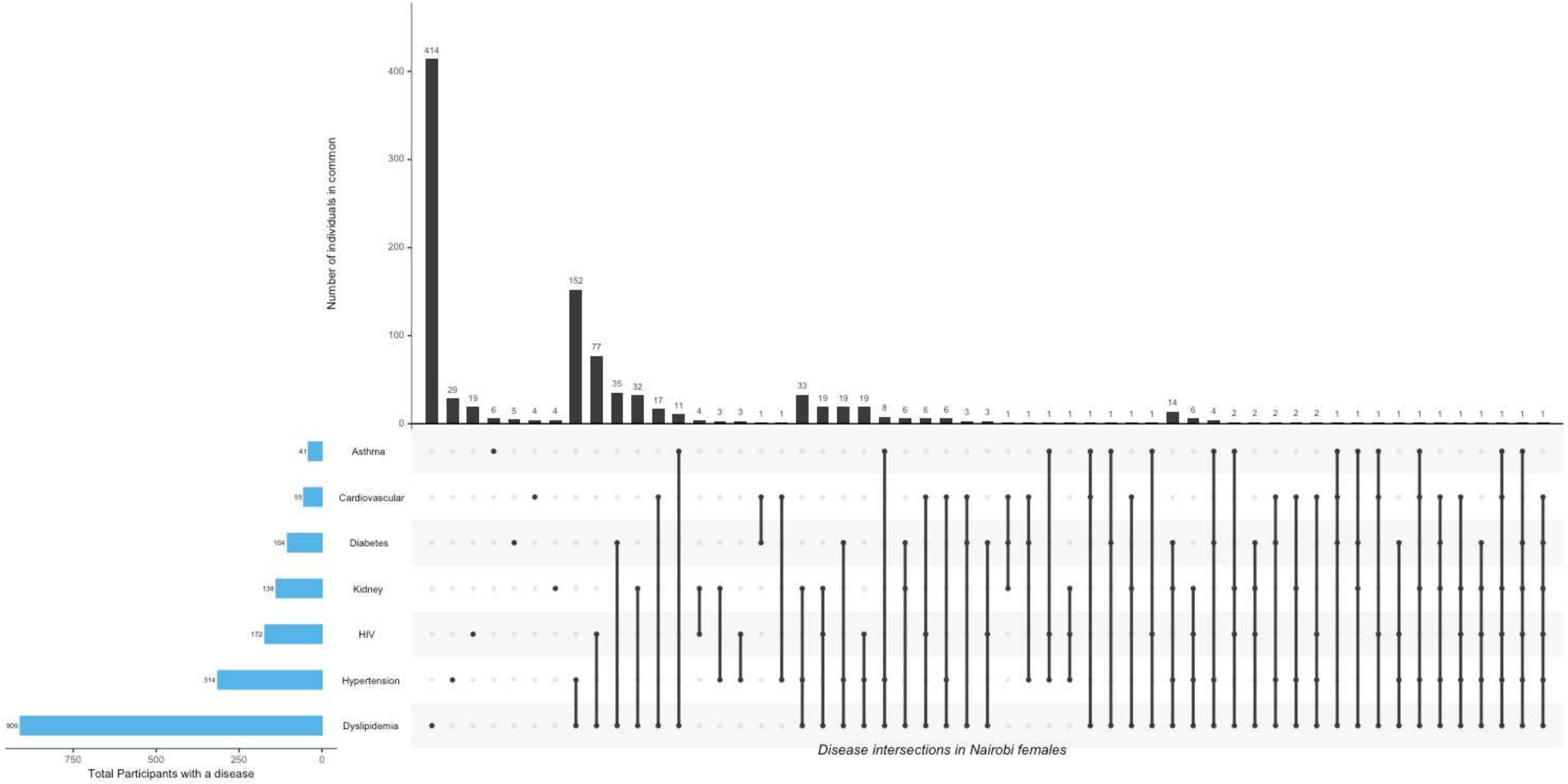

Supplementary figure 9

i

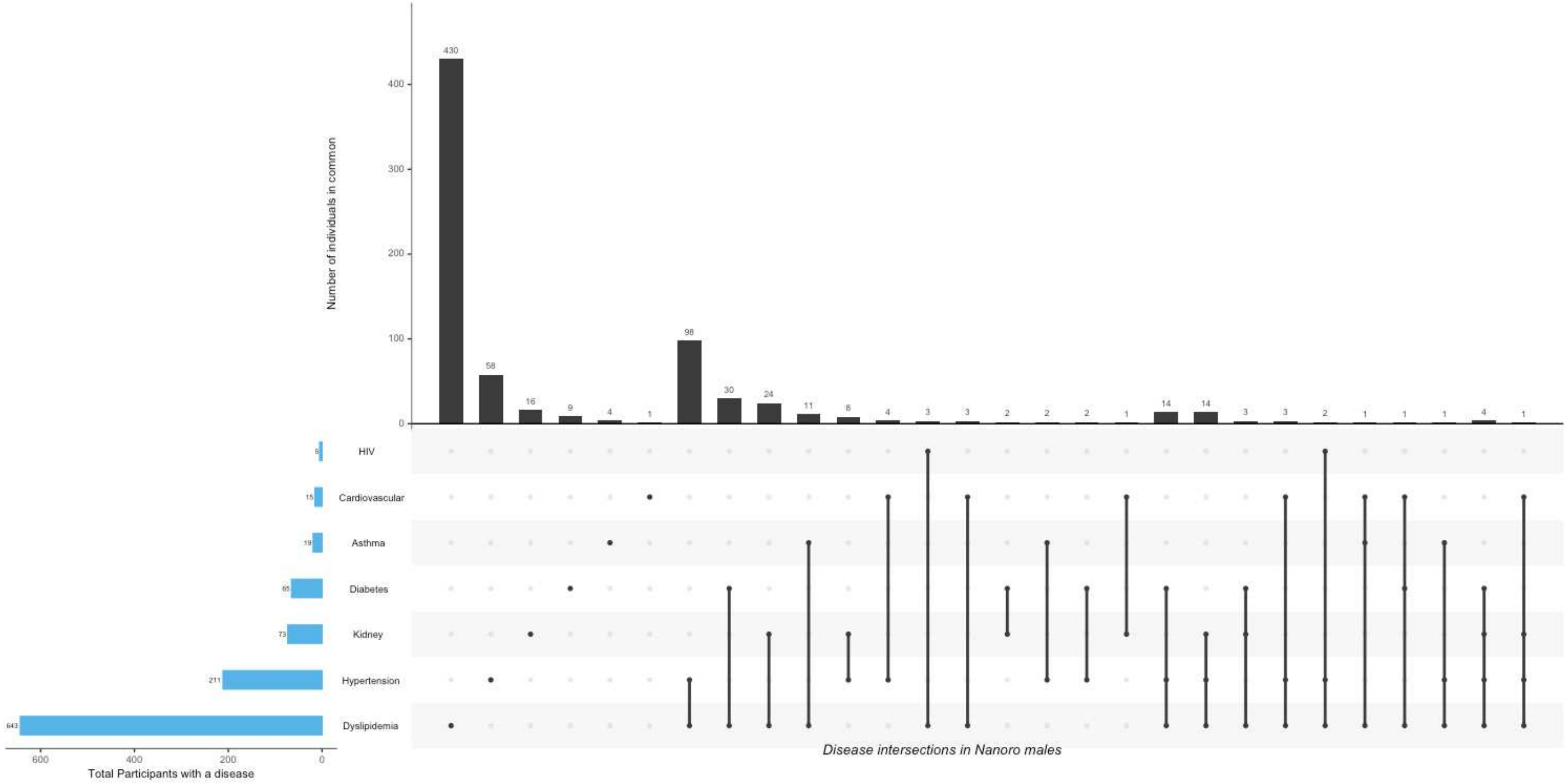

Supplementary figure 10

j

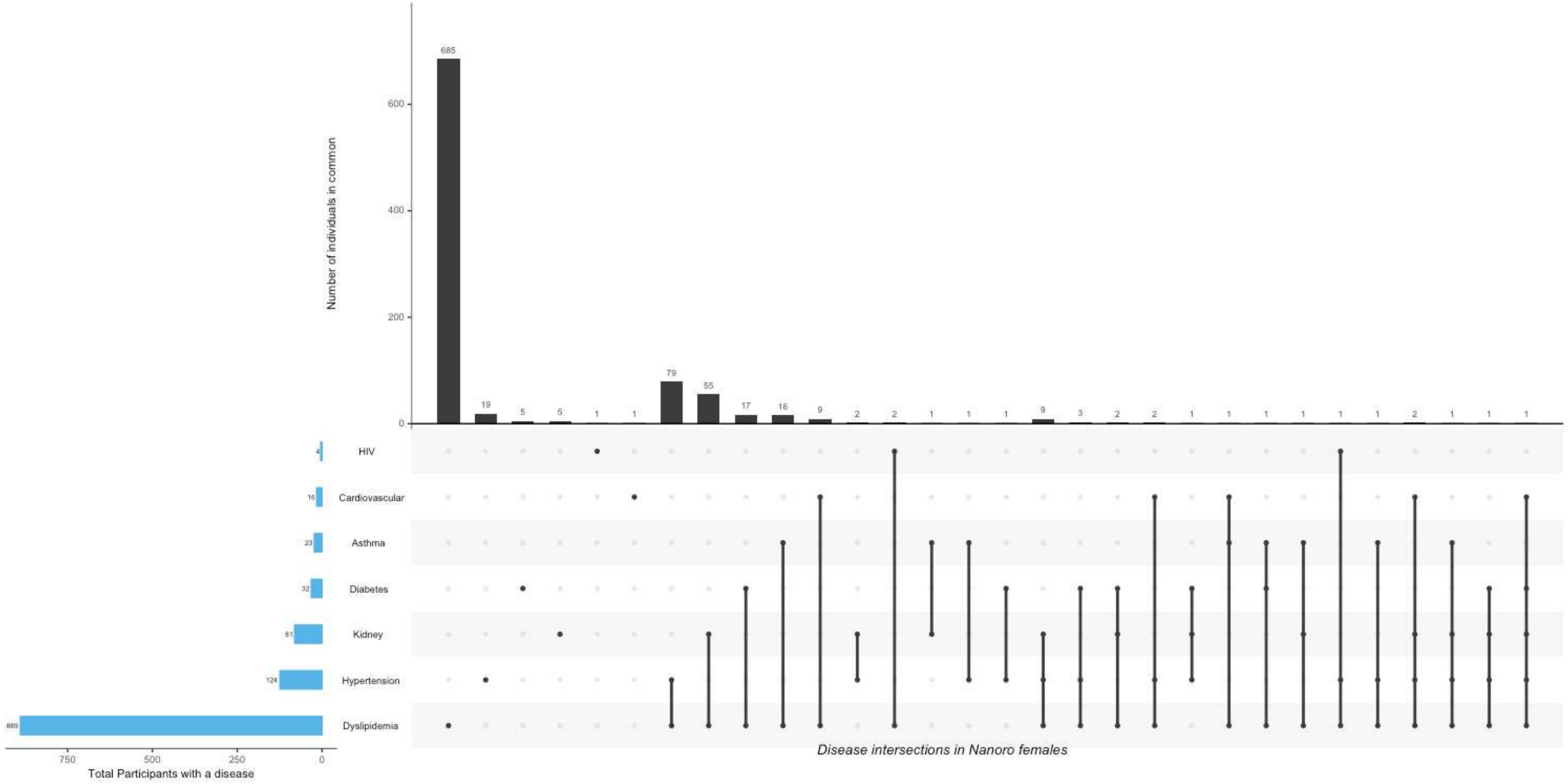

Supplementary figure 11

k

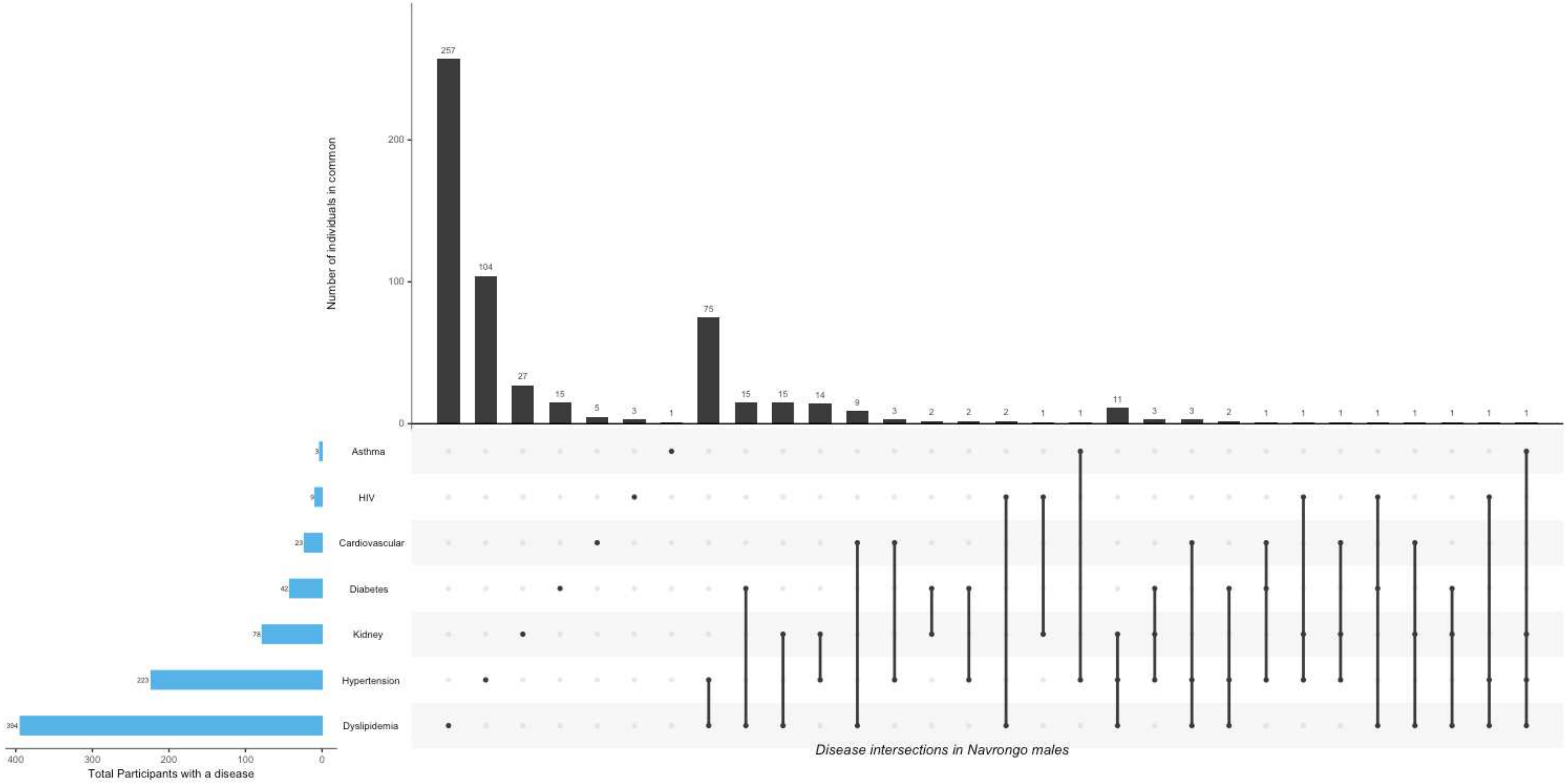

Supplementary figure 12

I

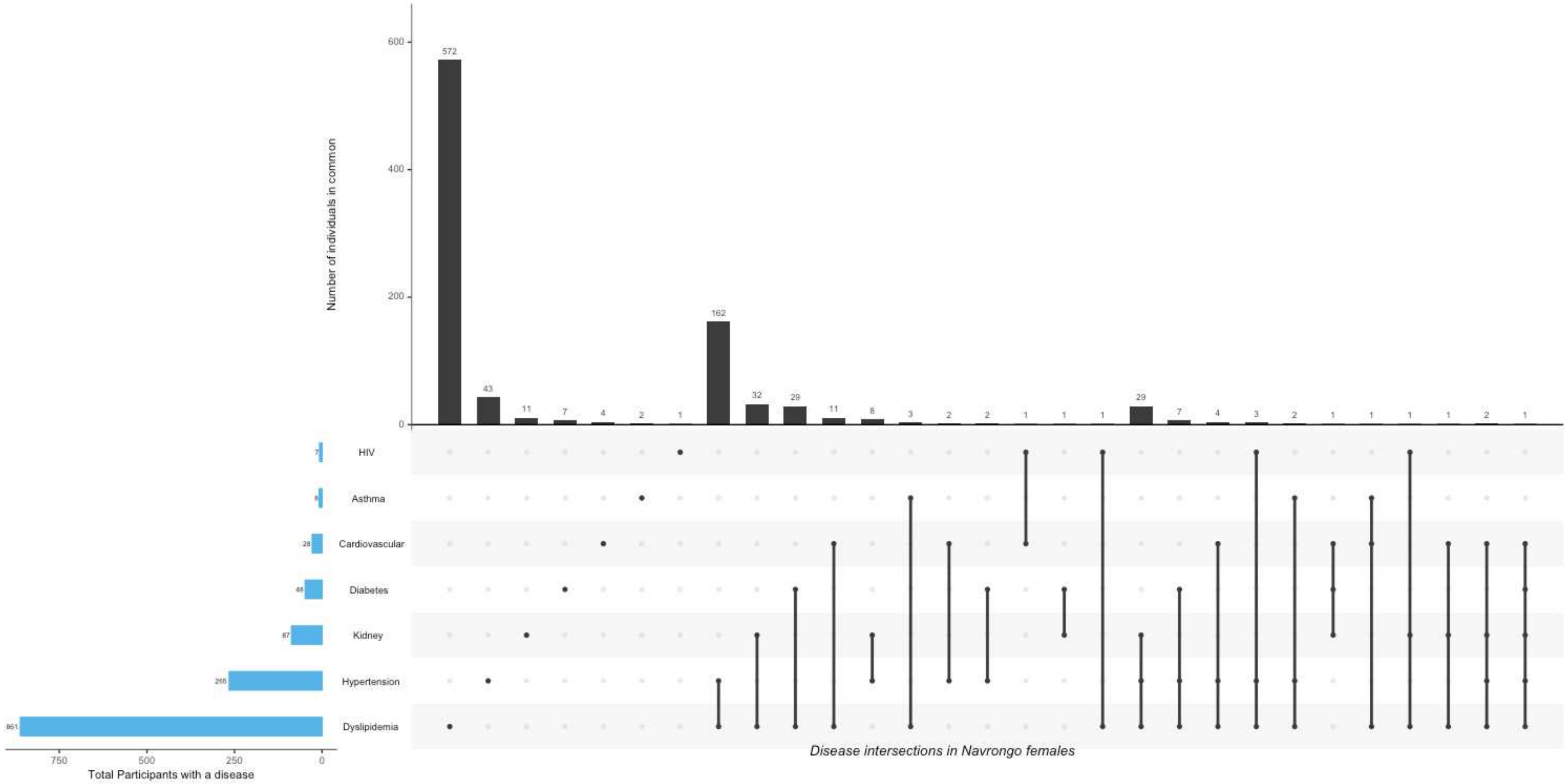

Supplement: Supplementary data [file bmjopen-2022-067788supp001.pdf]
