# Supplementary material for: Identifying the prevalence and correlates of multimorbidity in middle-aged men and women: a cross-sectional population-based study in four African countries
Source: BMJ Open. 2023 Mar 14;13(3):e067788. doi: 10.1136/bmjopen-2022-067788 (PMC10016250; doi:10.1136/bmjopen-2022-067788)
Supplement: Author's manuscript [file bmjopen-2022-067788.draft_revisions.pdf]

BMJ Open is committed to open peer review. As part of this commitment we make the peer review history of every article we publish publicly available.

When an article is published we post the peer reviewers' comments and the authors' responses online. We also post the versions of the paper that were used during peer review. These are the versions that the peer review comments apply to.

The versions of the paper that follow are the versions that were submitted during the peer review process. They are not the versions of record or the final published versions. They should not be cited or distributed as the published version of this manuscript.

BMJ Open is an open access journal and the full, final, typeset and author-corrected version of record of the manuscript is available on our site with no access controls, subscription charges or pay-per-view fees (http://bmjopen.bmj.com).

If you have any questions on BMJ Open's open peer review process please email info.bmjopen@bmj.com

BMJ Open

Identifying the prevalence and correlates of multimorbidity in middle-aged men and women: a cross-sectional population-based study in four African countries

Journal:	*BMJ Open*
Manuscript ID	bmjopen-2022-067788
Article Type:	Original research
Date Submitted by the Author:	29-Aug-2022
Complete List of Authors:	Micklesfield, Lisa; University of the Witwatersrand Johannesburg Faculty of Health Sciences, SAMRC/Wits Developmental Pathways for Health Research Unit, Department of Paediatrics<br>Munthali, Richard; University of the Witwatersrand Johannesburg Faculty of Health Sciences, SAMRC/Wits Developmental Pathways for Health Research Unit, Department of Paediatrics<br>Agongo, Godfred; C K Tedam University of Technology and Applied Sciences, Department of Biochemistry and Forensic Sciences; Ghana Health Service, Navrongo Health Research Centre<br>Asiki, G; African Population and Health Research Center<br>Boua, Palwende; Institut de Recherche en Sciences de la Santé, Clinical Research Unit of Nanoro; University of the Witwatersrand Johannesburg, Sydney Brenner Institute of Molecular Bioscience<br>Choma, Solomon SR; University of Limpopo, Department of Pathology<br>Crowther, Nigel; University of the Witwatersrand Johannesburg Faculty of Health Sciences, Department of Chemical Pathology<br>Fabian, June; University of the Witwatersrand Johannesburg, Wits Donald Gordon Medical Centre<br>Gómez-Olivé, F. Xavier; University of the Witwatersrand Johannesburg Faculty of Health Sciences, Medical Research Council/Wits Rural Health and Health Transitions Unit (Agincourt), School of Public Health, Faculty of Health Sciences<br>Kabudula, Chodziwadziwa; University of the Witwatersrand School of Public Health, MRC/Wits Rural Public Health and Health Transitions Research Unit (Agincourt),<br>Maimela, Eric; University of Limpopo, Department of Pathology<br>Mohamed, Shukri; African Population and Health Research Center, Health and Systems for Health<br>Nonterah, Engelbert ; Ghana Health Service, Navongo Health Research Centre; Utrecht University, Julius Global Health, Julius Centre for Health Sciences and Primary Care<br>Raal, Frederick; University of the Witwatersrand Johannesburg Faculty of Health Sciences, Department of Medicine<br>Sorgho, Hermann; Institut de Recherche en Sciences de la Santé<br>Tluway, Furahini; University of the Witwatersrand Johannesburg Faculty of Health Sciences, Sydney Brenner Institute for Molecular Bioscience<br>Wade, Alisha N.; University of the Witwatersrand Johannesburg Faculty of Health Sciences, 9MRC/Wits Rural Public Health and Health Transitions Research Unit, School of Public Health

Norris, Shane; University of the Witwatersrand Faculty of Health Sciences, SAMRC/Wits Developmental Pathways for Health Research Unit, Department of Paediatrics
Ramsay, Michele; University of the Witwatersrand Johannesburg Faculty of Health Sciences, Sydney Brenner Institute for Molecular Bioscience

Keywords: EPIDEMIOLOGY, PUBLIC HEALTH, Lipid disorders < DIABETES & ENDOCRINOLOGY

SCHOLARONE™ Manuscripts

I, the Submitting Author has the right to grant and does grant on behalf of all authors of the Work (as defined in the below author licence), an exclusive licence and/or a non-exclusive licence for contributions from authors who are: i) UK Crown employees; ii) where BMJ has agreed a CC-BY licence shall apply, and/or iii) in accordance with the terms applicable for US Federal Government officers or employees acting as part of their official duties; on a worldwide, perpetual, irrevocable, royalty-free basis to BMJ Publishing Group Ltd ("BMJ") its licensees and where the relevant Journal is co-owned by BMJ to the co-owners of the Journal, to publish the Work in this journal and any other BMJ products and to exploit all rights, as set out in our licence.

The Submitting Author accepts and understands that any supply made under these terms is made by BMJ to the Submitting Author unless you are acting as an employee on behalf of your employer or a postgraduate student of an affiliated institution which is paying any applicable article publishing charge ("APC") for Open Access articles. Where the Submitting Author wishes to make the Work available on an Open Access basis (and intends to pay the relevant APC), the terms of reuse of such Open Access shall be governed by a Creative Commons licence – details of these licences and which Creative Commons licence will apply to this Work are set out in our licence referred to above.

Other than as permitted in any relevant BMJ Author's Self Archiving Policies, I confirm this Work has not been accepted for publication elsewhere, is not being considered for publication elsewhere and does not duplicate material already published. I confirm all authors consent to publication of this Work and authorise the granting of this licence.

For peer review only

Identifying the prevalence and correlates of multimorbidity in middle-aged men and women: a cross-sectional population-based study in four African countries

Running title: Multimorbidity in African adults

Lisa K. Micklesfield[1], Richard Munthali[1], Godfred Agongo[2,3], Gershim Asiki[4], Palwende Romuald Boua[5,12], Solomon SR Choma[6], Nigel J. Crowther[7], June Fabian[8], F. Xavier Gomez-Olive[9], Chodziwadziwa W Kabudula[9], Eric Maimela[6], Shukri F Mohamed[4], Engelbert A. Nonterah[3,10], Frederick Raal[11], Hermann Sorgho[5], Furahini D. Tluway[12], Alisha N. Wade[9], Shane A. Norris[1], Michèle Ramsay[12] as members of AWI-Gen and the H3Africa Consortium.

[1]South African Medical Research Council/Wits Developmental Pathways for Health Research Unit, Department of Paediatrics, Faculty of Health Sciences, University of the Witwatersrand, Johannesburg, South Africa

[2]Department of Biochemistry and Forensic Sciences, C.K. Tedam University of Technology and Applied Sciences, Navrongo, Ghana

[3]Navrongo Health Research Centre, Ghana Health Service, Navrongo, Ghana

[4]African Population and Health Research Center, Nairobi

[5]Clinical Research Unit of Nanoro, Institut de Recherche en Sciences de la Santé, Burkina Faso

[6]DIMAMO Health and Population Research Centre, Department of Pathology, Faculty of Health Sciences University of Limpopo

[7]Department of Chemical Pathology, National Health Laboratory Service, University of the Witwatersrand Faculty of Health Sciences, Johannesburg

[8]Wits Donald Gordon Medical Centre, School of Clinical Medicine, Faculty of Health Sciences, 7 York Rd, Parktown, 2193, Johannesburg, South Africa

[9]MRC/Wits Rural Public Health and Health Transitions Research Unit, School of Public Health, Faculty of Health Sciences, University of the Witwatersrand, 27 St. Andrew's Road, Parktown, Johannesburg 2193, South Africa

[10]Julius Global Health, Julius Centre for Health Sciences and Primary Care, University Medical Centre Utrecht, Utrecht University, Utrecht, the Netherlands

[11]Carbohydrate & Lipid Metabolism Research Unit, Division of Endocrinology & Metabolism, Department of Medicine, Faculty of Health Sciences, University of the Witwatersrand, Parktown, Johannesburg, South Africa

For peer review only

[12]Sydney Brenner Institute for Molecular Bioscience, Faculty of Health Sciences, University of the Witwatersrand, Johannesburg, South Africa

[#]**Corresponding author**: Professor Lisa Micklesfield, SAMRC/Wits Developmental Pathways for Health Research Unit, University of the Witwatersrand, Johannesburg, South Africa. Email: lisa.micklesfield@wits.ac.za; Tel: 0027 (0)11 9331122.

Key words: multimorbidity, Africa, chronic disease, prevalence, disease risk

Word count: 4594

For peer review only

Abstract

Objectives: To determine the prevalence of multimorbidity, to identify which chronic conditions cluster together, and to identify factors associated with a greater risk for multimorbidity in sub-Saharan Africa.

Design: Cross-sectional, multicentre, population-based study

Setting: Six urban and rural communities in four sub-Saharan African countries.

Participants: Men (n=4808) and women (n=5892) between the ages of 40 and 60 years from the AWI-Gen study.

Measures: Sociodemographic and anthropometric data, and multimorbidity as defined by the presence of two or more of the following conditions: HIV infection, cardiovascular disease, chronic kidney disease, asthma, diabetes, dyslipidaemia, hypertension.

Results: Multimorbidity prevalence was higher in women compared to men (47.2 vs. 35%), and higher in South African men and women compared to their East and West African counterparts. The most common disease combination at all sites was dyslipidaemia and hypertension, with this combination being more prevalent in South African women than any single disease (25 vs 21.6%). Age and BMI were associated with a higher risk of multimorbidity in men and women, however lifestyle correlates such as smoking and physical activity were different between the sexes.

Conclusions: The high prevalence of multimorbidity in middle-aged adults in sub-Saharan Africa is of concern, with women currently being at higher risk. This is expected to increase in men, as well as the East and West African region with the ongoing epidemiological transition, and identifying common disease clusters and correlates of multimorbidity is critical to providing effective interventions.

For peer review only

Strengths and limitations of this study

- A strength of the study is that it provides multimorbidity prevalence data on a large (n=11 273), population-based sample of middle-aged men and women from sub-Saharan Africa.
- Study sites include urban and rural communities from South, East and West Africa.
- A limitation is the cross-sectional study design with some of the diseases measured via self-report with no longitudinal follow-up.
- The cross-sectional study design only allows for the identification of correlates of multimorbidity, rather than causal factors.

For peer review only

Introduction

Sub-Saharan Africa is experiencing the highest rate of urbanisation globally and together with an increase in life expectancy must prepare for the prevalence of non-communicable diseases (NCDs) to increase further (1). Multimorbidity, the co-occurrence of two or more chronic diseases in one individual, is common, challenging the affected individual, their attending healthcare professionals, and an overstretched health system. A recent systematic review highlighted a paucity of multimorbidity costing studies from low- and middle-income countries, but concluded that multimorbidity places an enormous economic burden on health systems with higher country GDP per capita associated with higher costs of multimorbidity (2). When describing global patterns of multimorbidity Afshar et al., reported a positive but non-linear association between country GDP and multimorbidity prevalence, and also identified an inverse association between multimorbidity and SES in countries with the highest GDP, with the gradient sometimes reversed in countries with lower GDP(3). Understanding the social and structural forces that drive the clustering of diseases thereby exacerbating their impact on disease outcomes, is defined as syndemics(4), and is critical to our understanding of multimorbidity.

Recent data from low- and middle-income countries (LMICs) have been reported in a scoping review of NCD multimorbidity, which ranged in prevalence from 3.2% to 90.5% (5). The studies included in this review used various chronic diseases comprising the multimorbidity profile, and different diagnostic criteria for each chronic disease, highlighting the need for a consensus on the definition of multimorbidity and the core conditions that it is comprised of. Further the inclusion of chronic infectious diseases such as HIV and tuberculosis should also be considered when quantifying the burden of multimorbidity but may be more relevant in some settings than others. There is a dearth of multimorbidity prevalence data from Africa, with studies reporting data from single countries such as South Africa (6,7), Ghana (8), Burkina Faso (9), and Kenya (10). Although the influence of age and socioeconomic status on multimorbidity prevalence are well recognised globally (3,5,11,12), several African studies have also identified associations between lifestyle factors, mental health and multimorbidity (7,10,12). Comparing and contrasting different African settings and identifying which diseases cluster together and the factors associated with this clustering, will assist in the design of effective interventions.

The double burden of non-communicable and infectious diseases as well as the impact of urbanisation on disease risk, highlights the need to undertake multimorbidity research in Africa,

For peer review only

and the heterogeneity in the epidemiological and nutrition transition, and disease burden of different African countries, provides an opportunity to compare and contrast correlates and prevalence's of multimorbidity across different African settings. The aim of this study was to determine the prevalence of multimorbidity; to identify which chronic conditions cluster together; and to identify factors associated with a greater risk for multimorbidity in six rural and urban communities in four SSA countries. This study will contribute to the evidence-base on multimorbidity by providing prevalence data across different African settings and identifying common disease clusters to potentially understand the pathogenesis of different diseases as well as in the design of more effective interventions. This formative data on multimorbidity can also inform policymakers in the planning and implementation of more effective health systems with particular relevance to other low- and middle- income countries.

For peer review only

Methods

Study population

Data included in this study are from the Africa Wits-INDEPTH (University of the Witwatersrand, Johannesburg, and the International Network for the Demographic Evaluation of Populations and Their Health) partnership for Genomic Studies (AWI-Gen) (13), which is a National Institutes of Health funded Collaborative Centre of the Human Heredity and Health in Africa (H3Africa) Consortium. AWI-Gen is a population-based cross-sectional study of adults and includes six participating urban and rural centres in four Sub-Saharan African countries. West Africa included two countries, Ghana (Navrongo) and Burkina Faso (Nanoro); East Africa included Kenya (Nairobi); and South Africa had three study sites (Soweto, Agincourt and Dikgale). Details of the recruitment strategy as well as other data collected as part of the larger study, are described in Ali et al.(14). For this study we only included participants between the ages of 40 and 60 years (n=11 273).

Socio-demographic and anthropometric data

Standard structured AWI-Gen questionnaires were administered by trained field staff with some country and site-specific modifications to suit their context (14). Data on self-reported socio-demographics (age, highest level of education attained, partnership status, employment status) were collected. Each study participant was assigned to a socio-economic status (SES) quintile which was determined for each study site by categorizing factor scores that were predicted from a principal component analysis of the number of household assets. Alcohol consumption was categorised as never, current problematic, current non-problematic or former consumer. Smoking of tobacco products was categorised as never, current or previous smoker. Total moderate-vigorous intensity physical activity (MVPA) was calculated as minutes per week from the accumulation of occupation, walking for travel and leisure time activity collected by self-report using the Global Physical Activity Questionnaire (GPAQ) (15). Weight and height were used to calculate body mass index (BMI: weight (kg)/height (m^2)).

Multimorbidity

We defined multimorbidity (MM) as the presence of two or more of the following seven conditions (HIV infection, cardiovascular disease, chronic kidney disease (CKD), asthma, diabetes, dyslipidemia and hypertension) for all sites except for women from Soweto, where data was only available for four of the conditions (HIV infection, diabetes, dyslipidemia and hypertension).

For peer review only - http://bmjopen.bmj.com/site/about/guidelines.xhtml

Obesity was not included as one of the conditions as in the context of this paper it is considered an NCD risk factor rather than a disease. For this reason, BMI was included as an independent variable in the multinomial regression.

HIV status was self-reported although participants in South Africa and Kenya were offered a voluntary government-approved rapid HIV test. Due to the low prevalence of HIV in Burkina Faso and Ghana participants were not offered HIV tests.

Hypertension was defined as systolic blood pressure equal or greater than 140 mm Hg and/or diastolic blood pressure equal or greater than 90 mm Hg, in line with the seventh report of the Joint National Committee on Prevention, Detection, Evaluation, and Treatment of High Blood Pressure, or if the participant was taking hypertension medication.

Diabetes was defined using the World Health Organisation criteria which are the presence of a previous diagnosis of diabetes by a healthcare professional or fasting blood glucose >=7 mmol/L or random glucose >=11.1 mmol/L, or on diabetes medication at the time of recruitment.

Dyslipidemia was defined as the presence of one of the following: total cholesterol (TC) ≥5.0mmol/L, or high-density lipoprotein cholesterol (HDL-C) <1.0mmol/L for men and <1.3mmol/L for women, or low-density lipoprotein cholesterol (LDL-C) ≥3. 0mmol/L, or triglycerides (TG) ≥1.7 mmol/L, or on lipid-modifying medication (16), or self-report of ever being diagnosed by a health professional with high cholesterol. The Randox Daytona Plus (Randox Laboratories Ltd, UK) autoanalyzer was used to analyze HDL-C, TG and TC on fasting venous blood samples. Low density lipoprotein cholesterol (LDL-C) was calculated using the Friedewald equation (17).

Chronic kidney disease was defined as estimated glomerular filtration rate (eGFR) <60 mL/min per 1.73 m^2 (calculated using the CKD-EPI (creatinine) equation 2009, without adjustment for African American ethnicity), presence of albuminuria (urine albumin creatinine ratio >3 mg/mmol) or both (18). These data were not available for Soweto women. As the study was cross-sectional, low EGFR and albuminuria were not confirmed with follow up testing. At each partner site, blood and urine specimens were processed and stored at -112°F. After completion of the study each partner site transported samples on dry ice to a central laboratory in Johannesburg, South Africa. At the central laboratory all analyses were batched and performed according to good laboratory practice with external monitoring for quality control. Serum and

For peer review only - http://bmjopen.bmj.com/site/about/guidelines.xhtml

urine creatinine (mg/dL) were measured using Jaffe's kinetic method calibrated to an isotope dilution mass spectrometry (IDMS)-traceable standard. Urinary albumin concentration was measured with immunoturbidimetry. The urine albumin to urine creatinine ratio (ACR; mg/mmol) was calculated from these measurements.

Cardiovascular disease (CVD) was defined as present if the participant reported having had a heart attack or stroke or transient ischemic attack (TIA). Participants previously diagnosed with congestive heart failure (CHF) or angina were also classified as having CVD. No data were available for angina or heart attack prevalence for the Soweto men so cardiovascular disease prevalence were calculated using the remaining data. For the Soweto women data were not available for cardiovascular disease.

Asthma was determined by self-report or use of medication for the condition. These data were not available for Soweto women.

Statistical analysis

Data were summarized using means and standard deviations (±SD) for continuous parametric data, and median (interquartile range) for non-parametric data. A two-sided Students t-test was used to test for differences between men and women on parametric continuous variables within each site while Kruskal–Wallis test was used for non-parametric data. Chi-square test was used for categorical data. Due to significant sex differences within sites all further analyses were stratified by sex. Since the multimorbidity outcome had three categories, zero, one, and at least two conditions of the seven conditions under study, multinomial logistic regression was used to explore the factors associated with either one condition or at least two conditions, with none as the reference group, in men and women separately. A *P*-value less than 0.05 was considered statistically significant in all tests carried out using STATA version 14.1 SE (Stata Corporation, College Station, TX).

To understand the different multimorbidity patterns between men and women, within and between countries, and between regions i.e. South (South African sites), East (Kenyan site), and West (Ghana and Burkina Faso) Africa, different diseases were plotted using UpSetR, an R package (19). Separate analyses were done for men and women, separated by site and then geographic region.

For peer review only

Ethics and consent

All participants provided written informed consent before any study procedures were conducted. The Human Research Ethics Committee (Medical) of the University of the Witwatersrand approved the protocol (certificate numbers M121029 and M170880). Each study centre obtained local ethics approval as detailed in Ali et al. 2018 (14).

Patient and public involvement

This study did not involve any patients and/or the public.

For peer review only

Results

Socio-demographic and lifestyle factors

Only participants between the ages of 40 and 60 years were selected for these analyses (n=11 273), and the median age ranged from 48 years in Nairobi to 51 years in Agincourt, Dikgale and Navrongo (Supplementary tables 1 and 2). The only site where there was a significant sex difference in age was Navrongo where women were older than men. Sex differences in education level attained were significant at all sites, except Dikgale where the majority of participants had completed secondary school (55.2%), while in Navrongo and Nanoro the majority of the participants did not have any formal education. Employment was above 90% in both Nairobi and Nanoro, 62.6% and 60% in Navrongo and Soweto, respectively and only 36.1% in Agincourt and 37.7% in Dikgale. There were significant sex differences in SES at all six sites. Smoking and alcohol consumption were significantly different between the sexes at all sites except Soweto (alcohol status was not known in the women), as previously reported (20).

Multimorbidity prevalence

For the total study population as well as when the sites were combined by geographical area, multimorbidity prevalence was higher in women compared to men, with nearly 50% of the women in the total sample presenting with multimorbidity compared to 35% of the men (Figure 1. Multimorbidity prevalence was highest in the South African men and women (51.7% and 64.9%, respectively), followed by East Africa (31.3% and 48.4%, respectively), and then West Africa (20.2% and 24.1%, respectively). Overall, the site with the highest multimorbidity prevalence was Agincourt with 66.6%, and the site with the lowest prevalence was Nanoro with 21.2% (Supplementary tables 3 and 4).

Multimorbidity clusters

Multimorbidity clustering for the total study population, and for the 3 geographical regions, stratified by sex, are presented in Figures 2 and 3, respectively.

Study population, stratified by sex (Figure 2)

The majority of the total study population of men (42.9%) and women (44.8%) had one disease only, and in those with only two diseases the most common combination was dyslipidemia and hypertension (men 12%; women 18%) (Figure 2a and b). While in men, the most common

For peer review only

combination of three diseases was dyslipidemia, hypertension, and chronic kidney disease (2% of the total study population), in women the most common combination was dyslipidemia, hypertension, and HIV infection (3.2%). The prevalence of four or more diseases was marginally higher in women compared to men (3.3% vs. 2.1%) with the most common cluster of four diseases being dyslipidemia, hypertension, HIV, and chronic kidney disease in both sexes.

Geographical regions, stratified by sex (Figure 3)

The majority of men (51.7%) and women (64.9%) living in South Africa presented with multimorbidity (Supplementary Table 2, Figure 1), with two diseases (hypertension and dyslipidemia) (Figure 3a and b) as the most common combination in the men (33.4%) and women (41.3%). In contrast the majority of men (51.1%) and women (45.6%) from East Africa reported only one disease, although the pattern in those with two diseases was similar to South Africa as dyslipidemia and hypertension were the most common cluster (Figures 3c and d), and this was the same in West Africa (Figures 3e and f). It is in the clustering of three or more diseases that there are differences between the sites as the most common clustering of three diseases in South African men and women was dyslipidemia, hypertension and HIV, while in East and West Africa dyslipidemia and hypertension were most commonly clustered with chronic kidney disease.

Sites, stratified by sex

At all sites except Nanoro the prevalence of multimorbidity was higher in women than men, with the prevalence being highest in Agincourt women (74.1%) and lowest in men from Navongo (18%) (Supplementary tables 3 and 4). In all the sites the most common disease combination in men and women with two diseases only was dyslipidemia and hypertension (Supplementary figures 1 to 12), with the lowest prevalence in Nanoro women (7.6%) and the highest prevalence in Soweto women (30.5%). There were differences between the sites in the most common disease clusters in men and women with three diseases only, with dyslipidemia and hypertension clustered with CKD in Soweto men, Nairobi men and women, Navrongo men and women, and Nanoro women. In men from Dikgale, and Agincourt men and women, the most common disease cluster in those with only three diseases was dyslipidemia, hypertension and HIV, while in Dikgale and Soweto women, and Nanoro men, it was dyslipidemia, hypertension and diabetes. The highest prevalence with four or more diseases was reported in women from the South African sites: 8% of the Agincourt women and 7.6% of the Dikgale women (data was

For peer review only

not available for Soweto women). Except for Nairobi women, less than 1% of the men and women from the East and West African sites presented with four or more diseases.

Multinomial logistic regression, stratified by sex

Risk factors associated with having one condition and with having multimorbidity, compared to no condition, were determined using multinomial regression, and are presented for women (Table 1) and men (Table 2).

Table 1

	No disease vs. one disease			No disease vs. multimorbidity		
	RRR	95% CI	p value	RRR	95% CI	p value
Site (Ref: Agincourt)						
Dikgale	1.42	0.84, 2.43	0.185	0.77	0.46, 1.28	0.306
Nairobi	1.92	1.18, 3.11	0.009	0.58	0.36,0.93	0.023
Nanoro	3.10	1.78, 5.41	0.000	0.38	0.22,0.67	0.001
Navrongo	1.56	0.97, 2.52	0.068	0.25	0.16, 0.41	0.000
Age (yrs)	1.00	0.98, 1.02	0.932	1.04	1.02, 1.06	0.000
BMI (kg/m^2)	1.07	1.04, 1.10	0.000	1.11	1.08, 1.14	0.000
Smoking status (Ref: never smoked)						
Current smoker	0.58	0.27, 1.26	0.171	0.82	0.38, 1.80	0.628
Previous smoker	0.56	0.27, 1.18	0.126	0.76	0.37, 1.58	0.468
Alcohol consumption (Ref: never consumed)						
Current non-problematic consumer	0.86	0.64, 1.16	0.327	0.97	0.70, 1.34	0.851
Current consumer (problematic)	1.01	0.66, 1.55	0.959	1.14	0.72, 1.79	0.580
Former consumer	1.49	1.04, 2.12	0.028	1.97	1.37, 2.84	0.000
GPAQ MVPA (mins/wk/100)	0.98	0.99, 1.01	0.550	0.99	0.99, 1.00	0.132
Partnership status (Ref: never married or co-habited)						
Married or co-habiting	1.40	0.82, 2.4	0.223	1.13	0.66, 1.90	0.658
Divorced, separated, partner	1.62	0.93, 2.82	0.090	1.48	0.86, 2.54	0.157

For peer review only

deceased						
Highest level of education attained (Ref: no formal education)						
Primary education	1.12	0.80, 1.57	0.505	1.34	0.95, 1.90	0.094
Secondary education	1.31	0.84, 2.03	0.235	1.51	0.97, 2.36	0.067
Tertiary education	2.95	0.67, 12.9	0.152	2.81	0.65, 12.18	0.168
Employment status (Ref: unemployed)						
Employed	0.76	0.57, 1.02	0.069	0.79	0.59, 1.07	0.125
SES quintile (Ref: quintile 1)						
Quintile 2	0.96	0.68, 1.34	0.799	1.16	0.81, 1.66	0.412
Quintile 3	1.27	0.88, 1.83	0.195	1.35	0.92, 1.98	0.127
Quintile 4	0.99	0.70, 1.39	0.945	1.03	0.72, 1.48	0.857
Quintile 5	0.99	0.68, 1.42	0.940	1.15	0.78, 1.68	0.489

Table 2

	No disease vs. one disease			No disease vs. multimorbidity		
	RRR	95% CI	p value	RRR	95% CI	p value
**Site** (Ref: Agincourt)						
Dikgale	0.75	0.48, 1.17	0.204	0.59	0.38, 0.92	0.019
Nairobi	0.95	0.64, 1.40	0.781	0.42	0.28, 0.63	0.000
Nanoro	0.80	0.54, 1.18	0.256	0.29	0.19, 0.43	0.000
Navrongo	0.55	0.38, 0.80	0.002	0.16	0.11, 0.24	0.000
Soweto	1.15	0.75, 1.78	0.515	1.19	0.77, 1.84	0.422
Age (yrs)	1.01	0.99,1.02	0.369	1.03	1.02, 1.05	0.000
BMI (kg/m^2)	1.09	1.06, 1.12	0.000	1.14	1.11, 1.18	0.000
Smoking status (Ref: never smoked)						
Current smoker	1.07	0.87, 1.32	0.520	0.95	0.75, 1.20	0.671
Previous smoker	1.25	0.99, 1.58	0.061	1.17	0.91, 1.52	0.223

For peer review only

Alcohol consumption (Ref: never consumed)						
Current non-problematic consumer	0.66	0.52, 0.85	0.001	0.68	0.51,0.89	0.006
Current consumer (problematic)	0.63	0.47, 0.83	0.001	0.77	0.56, 1.06	0.105
Former consumer	1.09	0.81, 1.48	0.571	1.10	0.79, 1.54	0.568
GPAQ MVPA (mins/wk/100)	0.99	0.99, 0.10	0.015	0.99	0.99, 0.10	0.004
Partnership status (Ref: never married or co-habited)						
Married or co-habiting	1.39	1.01, 1.92	0.045	1.53	1.10, 2.15	0.011
Divorced, separated, partner deceased	1.61	1.08, 2.40	0.018	2.43	1.62, 3.65	0.000
Highest level of education attained (Ref: no formal education)						
Primary education	1.11	0.89, 1.40	0.355	1.30	1.00, 1.69	0.049
Secondary education	1.10	0.85, 1.44	0.460	1.22	0.91,1.64	0.183
Tertiary education	1.04	0.65, 1.68	0.858	1.10	0.67, 1.82	0.710
Employment status (Ref: unemployed)						
Employed	0.94	0.75, 1.17	0.555	0.77	0.61, 0.98	0.033
SES quintile (Ref: quintile 1)						
Quintile 2	1.09	0.83, 1.43	0.525	1.18	0.87, 1.61	0.293
Quintile 3	1.07	0.81, 1.41	0.630	1.08	0.79, 1.49	0.632
Quintile 4	1.12	0.86, 1.48	0.381	1.11	0.81, 1.51	0.512
Quintile 5	1.03	0.78, 1.36	0.823	1.303	0.96, 1.78	0.093

Due to the lower prevalence of multimorbidity in women from East and West Africa compared to South Africa, women living in Nairobi and Nanoro were more likely to have one condition compared to Agincourt women, and conversely, together with women from Navrongo, they were at a lower relative risk of multimorbidity compared to women from Agincourt. In women, being older was associated with a higher relative risk of multimorbidity, and a 1 kg/m^2 higher BMI was associated with a 7% higher risk of having one condition and an 11% higher risk of multimorbidity. Further, being a former consumer of alcohol compared to never consuming alcohol, was associated with a higher risk of having one disease, and with multimorbidity. None

For peer review only

of the other measures of socio-economic status or lifestyle behaviors were associated with multimorbidity risk in women.

When compared to the Agincourt men the relative risk of multimorbidity was lower at all sites, except Soweto. Similar to the women, older age and BMI were associated with a higher risk of multimorbidity with every year of age being associated with a 3% higher risk of multimorbidity, and a 1 kg/m^2 higher BMI was associated with a 9% higher risk of having one condition, and a 14% higher risk of multimorbidity. While smoking was not associated with disease risk the current consumption of alcohol, whether non-problematic or problematic, was associated with a lower risk of having one disease, and current non-problematic consumption of alcohol was associated with a 32% lower risk of multimorbidity. Another lifestyle behavior that was associated with disease risk was time spent in moderate-vigorous intensity physical activity, with more time spent in MVPA associated with a lower risk of having one condition, and with multimorbidity. Living with a partner either presently or in the past was associated with a higher risk of one condition and an even higher risk of multimorbidity when compared to not living with a partner. The attainment of primary school level education was associated with a 30% higher risk of multimorbidity compared to no formal education, and being employed was associated with a 33% lower risk of having multimorbidity compared to being unemployed, however there was no association between socio-economic status quintile and risk for one disease or multimorbidity.

Discussion

In this study of middle-aged men and women living in South, East and West Africa we have reported sex differences in the prevalence of multimorbidity, and identified socio-demographic, socio-economic and lifestyle factors associated with multimorbidity risk in men and women. While the prevalence of multimorbidity was more than 50% in South Africa, it was only just over 20% in West Africa, with East Africa reporting a prevalence of 31.3% in men and 48.4% in women. What was consistent across all sites was the higher prevalence of multimorbidity in women compared to men, and in both men and women age and BMI were independently associated with a higher risk of multimorbidity. These findings highlight the need for longitudinal studies of aging African populations to examine the effect of multimorbidity on mortality, as studies from high-income countries have shown that this is influenced by disease type, number and certain combinations that comprise multimorbidity (21,22). Our study has also shown that

For peer review only

the most commonly occurring disease cluster at all SSA sites was dyslipidaemia with hypertension, the prevalence of which was 12% in the total sample of men and 18% in women.

A recent systematic review of prevalence studies from South Africa reported a 3-23% prevalence of multimorbidity in studies with a wide range of age groups, and 30-71% in older adults (23). The higher prevalence of multimorbidity in the South African sites in the current study (54-66%) may be attributed to the advanced epidemiological transition and increasing urbanisation in South Africa (24). This coincides with a higher BMI and prevalence of obesity in the three South African sites compared to the West and East African sites (25). Despite being considered a rural site, Agincourt reported the highest prevalence of multimorbidity at 66.6%. This prevalence is similar to those reported from the same study site but including larger sample sizes of participants older than 40 years (n>3000) and different chronic diseases in the definition of multimorbidity (26,27). Kabudula et al., (28) have described a 'protracted' epidemiological transition in Agincourt between 1993 and 2013 as a result of the co-existence of infectious and non-communicable diseases, as well as the influence of social changes, and their results highlight the different transition experience in low and middle-income countries compared to high income countries (24). Although the West African sites, Ghana and Burkina Faso, reported the lowest prevalence of multimorbidity and a similar prevalence between men and women, it is expected that this will increase, following a similar trajectory to the South African sites. Mohamed et al., (10) have reported a lower multimorbidity prevalence of 28.7% (31.5% in women vs. 21.4% in men) in the same AWI-Gen study participants from Nairobi, Kenya. Reasons for the different prevalence compared to the current study is that they included 16 chronic conditions, and all measurements, except for hypertension and obesity, were via self-report. This may have resulted in an underestimation of multimorbidity prevalence as it is well recognised that many of these chronic diseases are undiagnosed in Africa (29–31).

Dyslipidaemia was the most commonly occurring disease across all the sites, with the prevalence ranging from 42.7% in men from Navrongo, to 87.4% in women from Dikgale. These results are similar to other African studies (32,33), with a recent study by Masilela et al., (34) reporting a prevalence of 76.7% in South African adults receiving care for diabetes and hypertension, and Reiger et al.,(35) reporting a prevalence of 67.3% in over 4000 adults aged 40 years and older from Agincourt, South Africa. Many of these African studies have reported that low HDL-C is the main driver of the high prevalence of dyslipidaemia, and it is important to debate whether the current international cut-offs for HDL-C, triglyceride and other cholesterol measures are relevant to the African population. A low HDL-C is frequently seen with obesity

For peer review only

and the metabolic syndrome and is associated with hypertriglyceridemia, particularly post-prandially (36). The main driver of atherosclerotic cardiovascular disease is LDL-C so this may be a more important marker of risk for cardiovascular disease (37). If this is the main criterion for defining dyslipidaemia and low HDL-C were excluded from the definition, then the prevalence of dyslipidaemia would be considered to have a lesser effect in the context of multimorbidity. Data from Agincourt has reported that while low HDL (<1.19 mmol/L) was prevalent in 26.5% of the adults aged 40 years and older, high LDL (>4.1 mmol/L) was measured in only 3.7% (38).

In the current study the most common cluster of two diseases at all sites was dyslipidaemia with hypertension, with this prevalence being higher than the prevalence of any single disease in Agincourt women, and Soweto men and women. This is in contrast to a recent systematic review from South Africa which identified the common disease clusters as hypertension and diabetes, hypertension and HIV, and TB and HIV (23). Irrespective of the difference in findings these studies highlight the high prevalence of hypertension in these large samples of middle-aged men and women from East, West and South Africa as previously reported by Gomez-Olive et al (31). A recent systematic review and meta-analysis assessing chronic kidney disease in African participants with hypertension provides strong evidence for the devastating outcomes of untreated hypertension as it reports a 17.8% pooled prevalence of chronic kidney disease in people with hypertension(39). In the total sample of men in the current study the most prevalent combination of three diseases was dyslipidaemia, hypertension and kidney disease (2% of the total sample) while in the women this was dyslipidaemia, hypertension and HIV (3% of the total sample). The co-occurrence of kidney disease and hypertension was more common than kidney disease and diabetes in both men and women, a finding which has previously been reported in Africa (40). Several multimorbidity patterns including a ‘cardio-respiratory’ pattern and a ‘metabolic’ pattern were identified across countries in the SAGE study reporting data on 12 chronic conditions, although the factors associated with multimorbidity differed between countries and could be explained by their diverse development status (11). Reporting on the South African data only Chidumwa et al., identified three groups using latent class analysis (7). While 88% of the sample were classified as minimal multimorbidity risk, 11% were classified as concordant (hypertension and diabetes) multimorbidity and 6% as discordant (angina, asthma, chronic lung disease, arthritis and depression) multimorbidity.

Identifying factors associated with multimorbidity clustering is important in designing country or region-specific strategies to manage multimorbidity. Results from this study as well as global

For peer review only

and African studies have reported an increased risk of multimorbidity with increasing age (10,27). Although this association is well accepted and understood, in the study by Garin et al., (11) multimorbidity prevalence decreased with age in the South African cohort, which they suggeseted may be due to the decrease in HIV prevalence with age. The current study also reported an 11-14% higher risk of multimorbidity with each 1kg/m^2 increase in BMI in both men and women. Overweight and obesity are well known risk factors for non-communicable diseases such as diabetes, hypertension and dyslipidaemia, particularly a low HDL-C, and obesity is a targeted risk factor by the World Health Organisation in their Global Action Plan for the prevention and control of NCDs (41). Although age and BMI were associated with multimorbidity prevalence in both men and women, lifestyle correlates displayed sex differences. In the women being a former consumer of alcohol was associated with nearly twice the risk of having multimorbidity, while men who were current, non-problematic consumers of alcohol were at lower risk of multimorbidity. Data from the same SSA cohort has reported that current alcohol consumption is lower in women compared to men at all AWI-Gen sites, with the characteristics of alcohol consumption such as type and frequency of consumption being significantly different between the sexes (20). This may explain the different associations with multimorbidity risk in the current study, and together with the finding that time spent in moderate-vigorous intensity physical activity was inversely associated with multimorbidity risk in men only, suggests that further intervention studies to reduce multimorbidity risk may need to be stratified by sex. The influence of socioeconomic patterns on disease risk is well recognised, and may differ between countries at different stages of the epidemiological transition (24). Employment was associated with a reduced risk of multimorbidity in this study and supports the inverse association between socio-economic status and disease risk reported by others in LMICs (42). We have shown that when compared to men who have never married or been in a union, men who are and men who have previously been in a union (divorced, separated, partner deceased) were at a significantly higher risk of multimorbidity. In their study of adults living in four sub-Saharan African countries Ajayi et al., (43) reported similar associations with BMI but in only two of the sites, rural and peri-urban Uganda, with no associations in adults from Tanzania and South Africa. We have previously shown in the AWI-Gen cohort that married men had a higher BMI compared to their unmarried counterparts, which consisted of those who had never been married or were no longer married (44), while results from the PURE study have shown that people who have experienced marital loss are at higher cardiovascular disease risk (45). Marital status may represent social position, socio-economic status as well as cultural beliefs

For peer review only

around body size and marriage, and these results highlight the complexity of the association with disease risk.

It is acknowledged that these data are cross-sectional and single screen testing without follow up may result in the overestimation of diseases such as chronic kidney disease. Further, only prevalence and correlates of multimorbidity could be identified, however the sample size is large and represents four sub-Saharan African countries to help understand differences and similarities between populations at different stages of the epidemiological transition. These prevalence figures make an important contribution to the increasing knowledge around multimorbidity and may be particularly useful in developing indices that can be used as part of machine learning approaches in predicting multimorbidity in the future.

In conclusion, this study has shown that nearly half of the population-based sample of women and more than a third of the men between the ages of 40 and 60 years from South, East and West Africa, are living with two or more chronic diseases. Common disease clusters have been identified and future efforts should focus on managing multiple commonly occurring diseases rather than single diseases. Although the prevalence of multimorbidity is higher in women living in South Africa, it is expected that as the obesity epidemic continues to increase that the prevalence will increase in men as well as East and West Africa. Identifying correlates of multimorbidity is critical to providing focussed and effective interventions.

Contributorship statement

LM, SN and MR conceptualized the study. RM did the analyses and prepared all the tables and figures. LM wrote the first manuscript draft and RM, SN and MR offered further guidance on the analyses, interpretation and writing. All authors reviewed the manuscript and approved the manuscript before submission.

Competing interests

None declared.

Funding

The AWI-Gen Collaborative Centre is funded by the National Human Genome Research Institute (NHGRI), the National Institute of Environmental Health Sciences (NIEHS), the Office of AIDS research (OAR) and the National Institute of Diabetes and Digestive and Kidney Diseases (NIDDK), of the National Institutes of Health (NIH) under award number

For peer review only

U54HG006938, as part of the H3Africa Consortium, and by the Department of Science and Innovation, South Africa, award number DST/CON 0056/2014. MR is a South African Research Chair in Genomics and Bioinformatics of African populations hosted by the University of the Witwatersrand, funded by the Department of Science and Technology and administered by National Research Foundation of South Africa (NRF). The Birth to Twenty Cohort (Soweto, South Africa) is supported by University of the Witwatersrand, the South African Medical Research Council, and The Wellcome Trust, UK. SAN is supported by the DST/NRF Centre of Excellence in Human Development at the University of the Witwatersrand, Johannesburg.
ANW is supported by the Fogarty International Centre, National Institutes of Health [grant number K43TW010698].

This paper describes the views of the authors and does not necessarily represent the official views of the National Institutes of Health or the National Research Foundation of South Africa who funded this research.

Data sharing statement

The data have been submitted to the European Genome-Phenome Archive (EGA), accession number EGA00001002482 (46). The Human Heredity and Health in Africa (H3Africa) Data and Biospecimen Access Committee (DBAC) will review requests for the AWI-Gen phenotype dataset. Related documents including study protocol and statistical analysis plan will be available upon request from the corresponding author.

Acknowledgements

This study would not have been possible without the generosity of the participants who spent many hours responding to questionnaires, being measured and having samples taken. We wish to acknowledge the contributions of our field workers, phlebotomists, laboratory scientists, administrators, data personnel and other investigators who contributed to the data and sample collections, processing, storage and shipping.

Investigators responsible for the conception and design of the AWI-Gen study include the following Michèle Ramsay (PI, Wits), Osman Sankoh (co-PI, INDEPTH), Stephen Tollman and Kathleen Kahn (Agincourt PI), Marianne Alberts (Dikgale (renamed DIMAMO) PI (deceased)), Catherine Kyobutungi (Nairobi PI), Halidou Tinto (Nanoro PI), Abraham Oduro (Navrongo PI), Shane Norris (Soweto PI), and Scott Hazelhurst, Nigel Crowther, Himla Soodyall and Zane Lombard (Wits). We would like to acknowledge each of the following investigators for the

For peer review only

significant contributions to this research, mentioned according to affiliation are: Wits AWI-Gen Collaborative Centre - Stuart Ali, Ananyo Choudhury, Scott Hazelhurst, Freedom Mukomana, Cassandra Soo; Soweto (DPHRU): Nomses Baloyi, Yusuf Guman.

For peer review only

References

1. Gouda HN, Charlson F, Sorsdahl K, Ahmadzada S, Ferrari AJ, Erskine H, et al. Burden of non-communicable diseases in sub-Saharan Africa,1990–2017: results from the Global Burden of Disease Study 2017. Lancet Glob Health. 2019;7:e1375–87.
2. Tran PB, Kazibwe J, Nikolaidis GF, Linnosmaa I, Rijken M, van Olmen J. Costs of multimorbidity: a systematic review and meta-analyses. BMC Med. 2022;20:234.
3. Afshar S, Roderick PJ, Kowal P, Dimitrov BD, Hill AG. Multimorbidity and the inequalities of global ageing: A cross-sectional study of 28 countries using the World Health Surveys. BMC Public Health. 2015;15:776.
4. Mendenhall E. Syndemics: a new path for global health research. Vol. 389, The Lancet. 2017;889–91.
5. Abebe F, Schneider M, Asrat B, Ambaw F. Multimorbidity of chronic non-communicable diseases in low- and middle-income countries: A scoping review. J Comorb. 2020;10:2235042X2096191.
6. Lalkhen H, Mash R. Multimorbidity in non-communicable diseases in South African primary healthcare. South African Medical Journal. 2015;105:134–8.
7. Chidumwa G, Maposa I, Corso B, Minicuci N, Kowal P, Micklesfield LK, et al. Identifying co-occurrence and clustering of chronic diseases using latent class analysis: Cross-sectional findings from SAGE South Africa Wave 2. BMJ Open. 2021;11:e041604.
8. Nimako BA, Baiden F, Sackey SO, Binka F. Multimorbidity of chronic diseases among adult patients presenting to an inner-city clinic in Ghana. Global Health. 2013;9:61.
9. Hien H, Berthé A, Drabo MK, Meda N, Konaté B, Tou F, et al. Prevalence and patterns of multimorbidity among the elderly in Burkina Faso: Cross-sectional study. Tropical Medicine and International Health. 2014;19:1328–33.
10. Mohamed SF, Haregu TN, Uthman OA, Khayeka-Wandabwa C, Muthuri SK, Asiki G, et al. Multimorbidity from chronic conditions among adults in urban slums: The Awi-Gen Nairobi site study findings. Glob Heart. 2021;16:6.
11. Garin N, Koyanagi A, Chatterji S, Tyrovolas S, Olaya B, Leonardi M, et al. Global Multimorbidity Patterns: A Cross-Sectional, Population-Based, Multi-Country Study. Journals of Gerontology - Series A Biological Sciences and Medical Sciences. 2016;71:205–14.
12. Alaba O, Chola L. The social determinants of multimorbidity in South Africa. Int J Equity Health. 2013;12:63.

For peer review only

13. Ramsay M, Crowther N, Tambo E, Agongo G, Baloyi V, Dikotope S, et al. H3Africa AWI-Gen Collaborative Centre: A resource to study the interplay between genomic and environmental risk factors for cardiometabolic diseases in four sub-Saharan African countries. Glob Health Epidemiol Genom. 2016;1e20.

14. Ali SA, Soo C, Agongo G, Alberts M, Amenga-Etego L, Boua RP, et al. Genomic and environmental risk factors for cardiometabolic diseases in Africa: methods used for Phase 1 of the AWI-Gen population cross-sectional study. Glob Health Action. 2018;11(sup2):1507133.

15. Bull FC, Maslin TS, Armstrong T. Global Physical Activity Questionnaire (GPAQ): Nine Country Reliability and Validity Study. Journal of Physical Activity and Health. 2009;6:790-804.

16. Klug EQ, Raal FJ, Marais AD, Smuts CM, Schamroth C, Jankelow D, et al. South African dyslipidaemia guideline consensus statement: 2018 update A joint statement from the South African Heart Association (SA Heart) and the Lipid and Atherosclerosis Society of Southern Africa (LASSA). South African Medical Journal. 2018;108:975–1000.

17. Friedewald WT, Levy RI, Fredrickson DS. Estimation of the concentration of Low-Density Lipoprotein Cholesterol in plasma, without use of the preparative ultracentrifuge. Clinical Chemistry 1972;18(6):499-502.

18. Stevens PE, Levin A. Evaluation and Management of Chronic Kidney Disease: Synopsis of the Kidney Disease: Improving Global Outcomes 2012 Clinical Practice Guideline. Ann Intern Med. 2013;158:825–30.

19. Conway JR, Lex A, Gehlenborg N. UpSetR: An R package for the visualization of intersecting sets and their properties. Bioinformatics. 2017 Sep 15;33:2938–40.

20. Boua PR, Soo CC, Debpuur C, Maposa I, Nkoana S, Mohamed SF, et al. Prevalence and socio-demographic correlates of tobacco and alcohol use in four sub-Saharan African countries: a cross-sectional study of middle-aged adults. BMC Public Health. 2021;21:1126.

21. Schäfer I, Kaduszkiewicz H, Nguyen TS, van den Bussche H, Scherer M, Schön G. Multimorbidity patterns and 5-year overall mortality: Results from a claims data–based observational study. J Comorb. 2018;8:2235042X1881658.

22. Willadsen TG, Siersma V, Nicolaisdóttir DR, Køster-Rasmussen R, Jarbøl DE, Reventlow S, et al. Multimorbidity and mortality: A 15-year longitudinal registry-based nationwide Danish population study. J Comorb. 2018;8: 2235042X18804063.

For peer review only

23. Roomaney RA, van Wyk B, Turawa EB, Pillay-van Wyk V. Multimorbidity in South Africa: a systematic review of prevalence studies. BMJ Open. 2021;11:e048676.
24. Stringhini S, Forrester TE, Plange-Rhule J, Lambert EV, Viswanathan B, Riesen W, et al. The social patterning of risk factors for noncommunicable diseases in five countries: Evidence from the modeling the epidemiologic transition study (METS). BMC Public Health. 2016;16:956.
25. Ramsay M, Crowther NJ, Agongo G, Ali SA, Asiki G, Boua RP, et al. Regional and sex-specific variation in BMI distribution in four sub-Saharan African countries: The H3Africa AWI-Gen study. Glob Health Action. 2018;11(sup2):1556561.
26. Chang AY, Gómez-Olivé FX, Payne C, Rohr JK, Manne-Goehler J, Wade AN, et al. Chronic multimorbidity among older adults in rural South Africa. BMJ Glob Health. 2019;4:e001386.
27. Wade AN, Payne CF, Berkman L, Chang A, Gómez-Olivé FX, Kabudula C, et al. Multimorbidity and mortality in an older, rural black South African population cohort with high prevalence of HIV findings from the HAALSI Study. BMJ Open. 2021;11:e047777.
28. Kabudula CW, Houle B, Collinson MA, Kahn K, Gómez-Olivé FX, Clark SJ, et al. Progression of the epidemiological transition in a rural South African setting: findings from population surveillance in Agincourt, 1993-2013. BMC Public Health. 2017;17:424.
29. Hoffman RM, Chibwana F, Kahn D, Banda BA, Phiri L, Chimombo M, et al. High Rates of Uncontrolled Blood Pressure in Malawian Adults Living with HIV and Hypertension. Glob Heart. 2021;16:81.
30. Stokes A, Berry KM, Mchiza Z, Parker W ah, Labadarios D, Chola L, et al. Prevalence and unmet need for diabetes care across the care continuum in a national sample of South African adults: Evidence from the SANHANES-1, 2011-2012. PLoS One. 2017;12:e0184264.
31. Gómez-Olivé FX, Ali SA, Made F, Kyobutungi C, Nonterah E, Micklesfield L, et al. Regional and Sex Differences in the Prevalence and Awareness of Hypertension: An H3Africa AWI-Gen Study Across 6 Sites in Sub-Saharan Africa. Glob Heart. 2017;12:81–90.
32. Chori B, Danladi B, Nwakile P, Okoye I, Abdullahi U, Zawaya K, et al. Prevalence, patterns and predictors of dyslipidaemia in Nigeria: a report from the REMAH study. Cardiovasc J Afr. 2022;33:14–21.

For peer review only

33. Fiseha T, Alemu W, Dereje H, Tamir Z, Gebreweld A. Prevalence of dyslipidaemia among HIV-infected patients receiving combination antiretroviral therapy in North Shewa, Ethiopia. PLoS One. 2021;16:e0250328
34. Masilela C, Adeniyi OV, Benjeddou M. Prevalence, patterns and determinants of dyslipidaemia among South African adults with comorbidities. Sci Rep. 2022;12:337.
35. Reiger S, Jardim TV, Abrahams-Gessel S, Crowther NJ, Wade A, Gomez-Olive FX, et al. Awareness, treatment, and control of dyslipidemia in rural South Africa: The HAALSI (Health and Aging in Africa: A Longitudinal Study of an INDEPTH Community in South Africa) study. PLoS One. 2017;12:e0187347.
36. Klop B, Elte JWF, Cabezas MC. Dyslipidemia in Obesity: Mechanisms and Potential Targets. Nutrients. 2013;5:1218–40.
37. Ference BA, Ginsberg HN, Graham I, Ray KK, Packard CJ, Bruckert E, et al. Low-density lipoproteins cause atherosclerotic cardiovascular disease. 1. Evidence from genetic, epidemiologic, and clinical studies. A consensus statement from the European Atherosclerosis Society Consensus Panel. Eur Heart J. 2017;38:2459–72.
38. Gaziano TA, Abrahams-Gessel S, Gomez-Olive FX, Wade A, Crowther NJ, Alam S, et al. Cardiometabolic risk in a population of older adults with multiple co-morbidities in rural South Africa: the HAALSI (Health and Aging in Africa: longitudinal studies of INDEPTH communities) study. BMC Public Health. 2017;17:1–10.
39. Ajayi SO, Ekrikpo UE, Ekanem AM, Raji YR, Ogah OS, Ojji DB, et al. Prevalence of Chronic Kidney Disease as a Marker of Hypertension Target Organ Damage in Africa: A Systematic Review and Meta-Analysis. Vol. 2021, International Journal of Hypertension. 2021:7243523.
40. Bikbov B, Purcell CA, Levey AS, Smith M, Abdoli A, Abebe M, et al. Global, regional, and national burden of chronic kidney disease, 1990–2017: a systematic analysis for the Global Burden of Disease Study 2017. The Lancet. 2020;395:709–33.
41. World Health Organisation. WHO Global NCD Action Plan 2013-2020. 2013.
42. Hosseinpoor AR, Bergen N, Kunst A, Harper S, Guthold R, Rekve D, et al. Socioeconomic inequalities in risk factors for non communicable diseases in low-income and middle-income countries: results from the World Health Survey. BMC Public Health. 2012;12:912.
43. Ajayi IOO, Adebamowo C, Adami HO, Dalal S, Diamond MB, Bajunirwe F, et al. Urban-rural and geographic differences in overweight and obesity in four sub-Saharan African

For peer review only

adult populations: a multi-country cross-sectional study. BMC Public Health. 2016;16:1–13.

44. Micklesfield LK, Kagura J, Munthali R, Crowther NJ, Jaff N, Gradidge P, et al. Demographic, socio-economic and behavioural correlates of BMI in middle-aged black men and women from urban Johannesburg, South Africa. Glob Health Action. 2018;11:1448250.

45. Egbujie BA, Igumbor EU, Puoane T. A cross-sectional study of socioeconomic status and cardiovascular disease risk among participants in the prospective Urban Rural Epidemiological (PURE) study. South African Medical Journal. 2016;106:900–6.

46. The European Genome-Phenome Archive (EGA). AWI-GEN Phase 1 Phenotype Dataset [Internet]. 2019 [cited 2021 May 31]. Available from: https://ega-archive.org/datasets/EGAD00001006425.

For peer review only

Figures

Figure 1: Prevalence of 0, 1, 2, 3 and 4 or more chronic conditions (including HIV infection, cardiovascular disease, kidney disease, asthma, diabetes, dyslipidemia and hypertension) in men and women from South, East and West Africa, and the full AWI-Gen cohort

Figure 2: Multimorbidity clustering for the total AWI-Gen cohort in a) women and b) men

Figure 3: Multimorbidity clustering in a) South African women; b) South African men; c) East African women; d) East African men; e) West African women; f) West African men

Supplementary Figure 1: Multimorbidity clustering in Agincourt men from the AWI-Gen study

Supplementary Figure 2: Multimorbidity clustering in Agincourt women from the AWI-Gen study

Supplementary Figure 3: Multimorbidity clustering in Dikgale men from the AWI-Gen study

Supplementary Figure 4: Multimorbidity clustering in Dikgale women from the AWI-Gen study

Supplementary Figure 5: Multimorbidity clustering in Soweto men from the AWI-Gen study

Supplementary Figure 6: Multimorbidity clustering in Soweto women from the AWI-Gen study

Supplementary Figure 7: Multimorbidity clustering in Nairobi men from the AWI-Gen study

Supplementary Figure 8: Multimorbidity clustering in Nairobi women from the AWI-Gen study

Supplementary Figure 9: Multimorbidity clustering in Nanoro men from the AWI-Gen study

Supplementary Figure 10: Multimorbidity clustering in Nanoro women from the AWI-Gen study

Supplementary Figure 11: Multimorbidity clustering in Navrongo men from the AWI-Gen study

Supplementary Figure 12: Multimorbidity clustering in Navrongo women from the AWI-Gen study

Tables

Table 1: Multinomial logistic regression to determine factors associated with either one condition or at least two conditions, with none as the reference group, in AWI-Gen women

Table 2: Multinomial logistic regression to determine factors associated with either one condition or at least two conditions, with none as the reference group, in AWI-Gen men

Supplementary Table 1: Descriptive characteristics of socio-demographic, socio-economic and lifestyle factors of men and women from the AWI-Gen Agincourt, Dikgale and Soweto sites

Supplementary Table 2: Descriptive characteristics of socio-demographic, socio-economic and lifestyle factors of men and women from the AWI-Gen Nairobi, Nanoro and Navrongo sites

Supplementary Table 3: Prevalence of the seven chronic diseases and multimorbidity of men and women from the AWI-Gen Agincourt, Dikgale and Soweto sites

For peer review only

Supplementary Table 4: Prevalence of the seven chronic diseases and multimorbidity of men and women from the AWI-Gen Nairobi, Nanoro and Navrongo sites

For peer review only

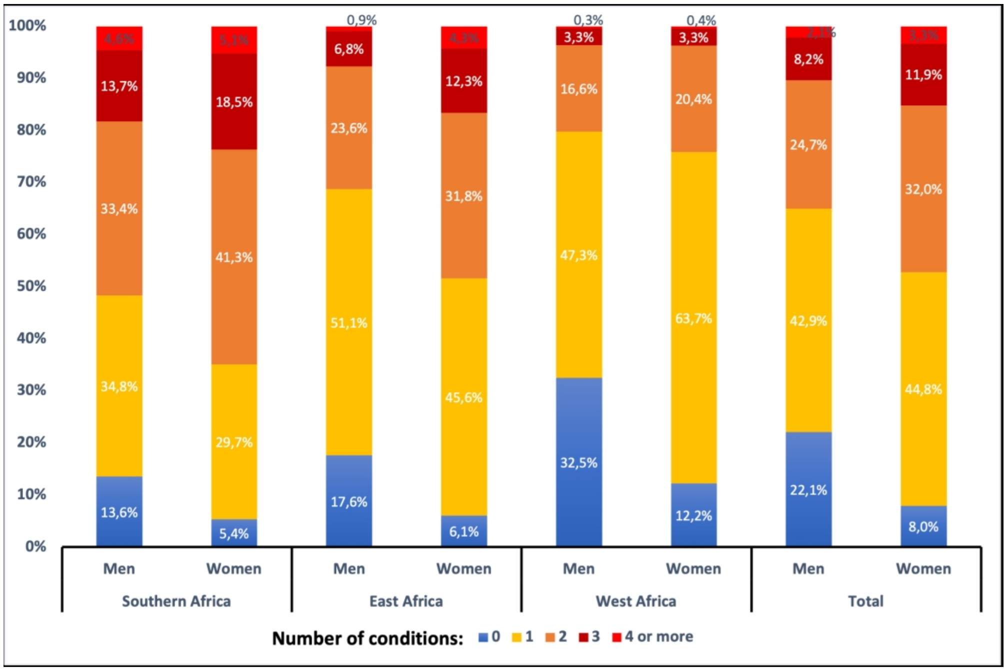

Prevalence of 0, 1, 2, 3 and 4 or more chronic conditions (including HIV infection, cardiovascular disease, kidney disease, asthma, diabetes, dyslipidemia and hypertension) in men and women from South, East and West Africa, and the full AWI-Gen cohort

479x317mm (130 x 130 DPI)

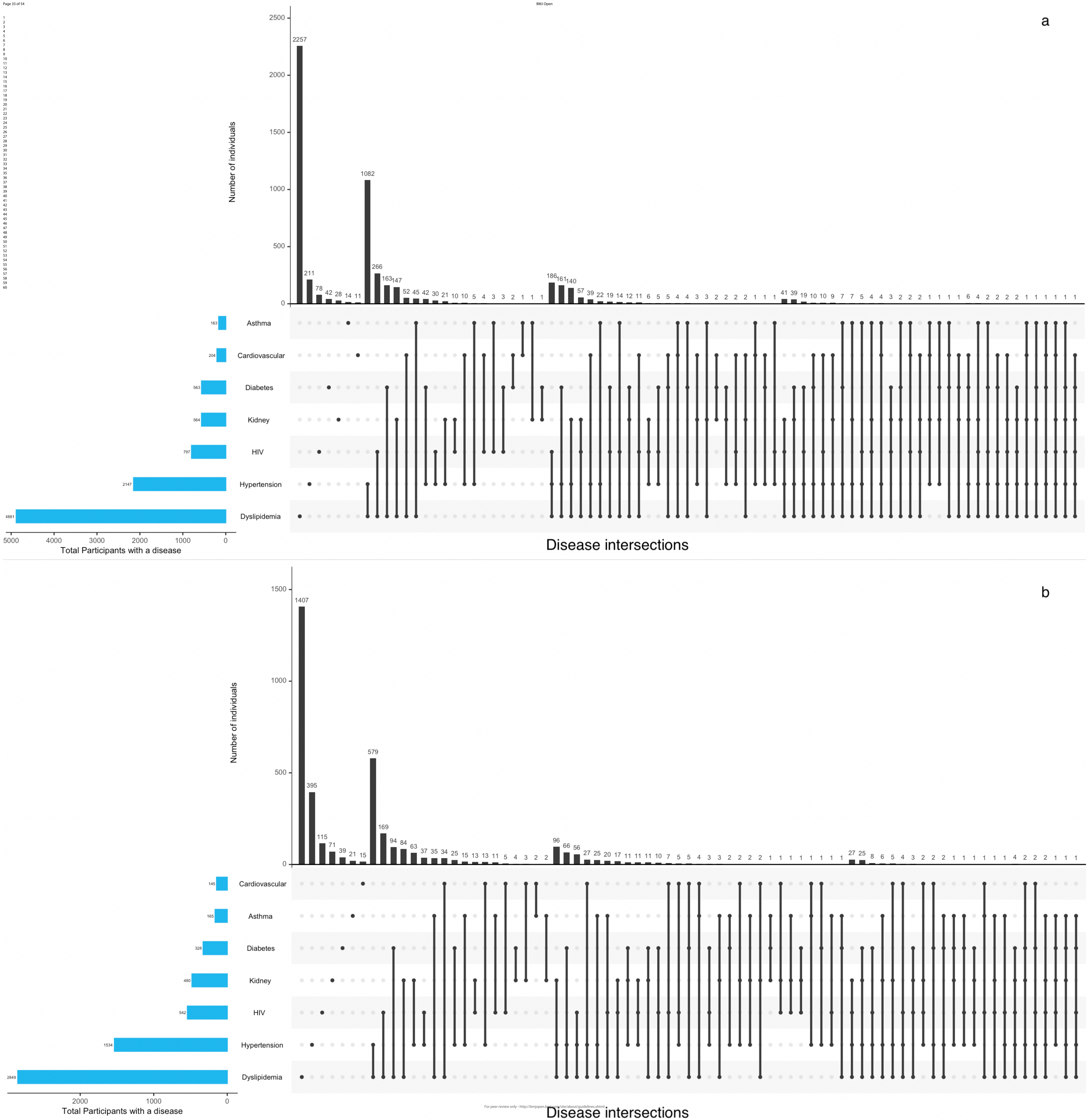
a
Number of individuals
Asthma
Cardiovascular
Diabetes
Kidney
HIV
Hypertension
Dyslipidemia
Total Participants with a disease
Disease intersections
b
Number of individuals
Cardiovascular
Asthma
Diabetes
Kidney
HIV
Hypertension
Dyslipidemia
Total Participants with a disease
Disease intersections

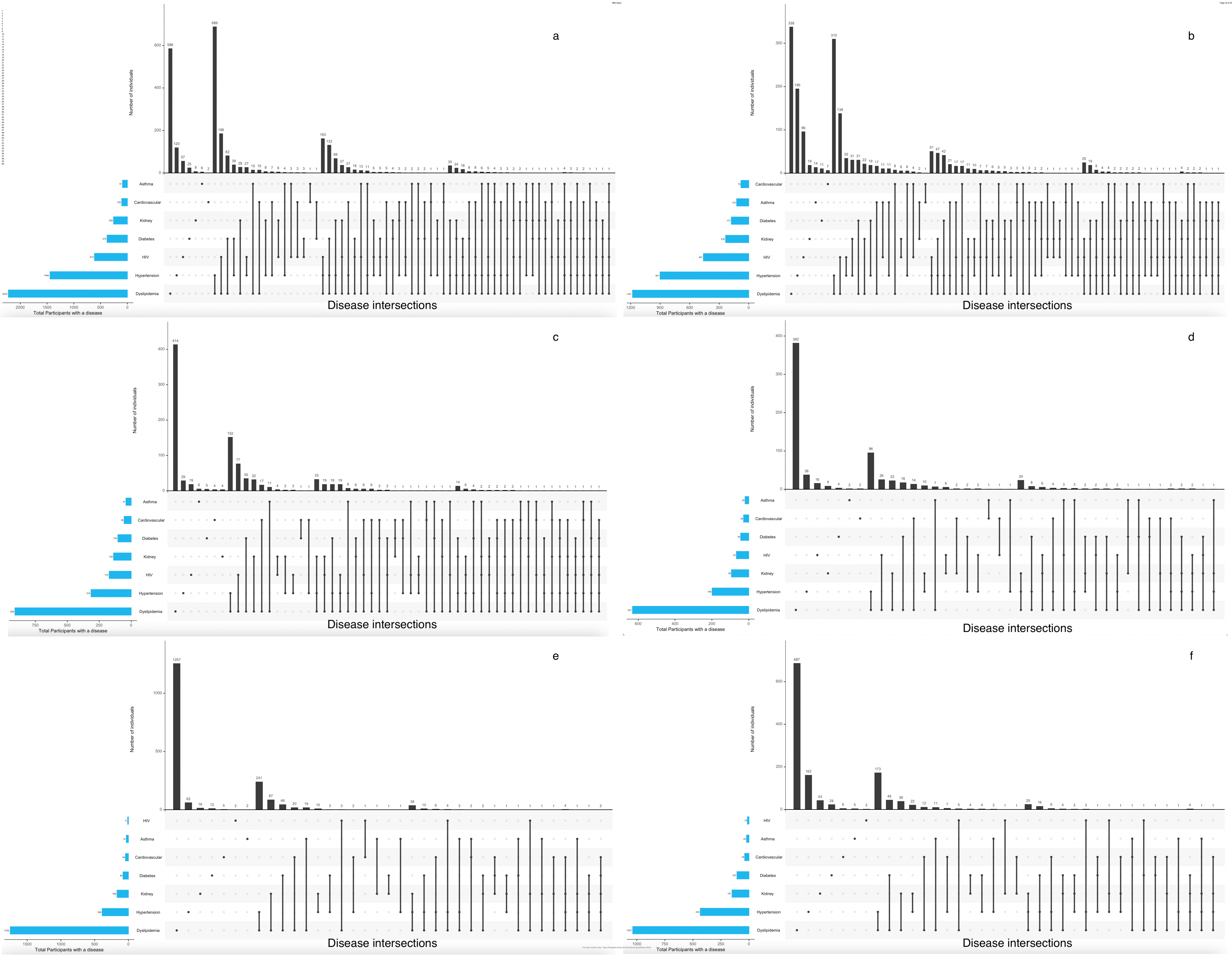
a
b
c
d
e
f
Number of individuals
Disease intersections
Total Participants with a disease
Asthma
Cardiovascular
Kidney
Diabetes
HIV
Hypertension
Dyslipidemia

Supplementary Table 1

	Agincourt, SA			Dikgale, SA			Soweto, SA		
	Men (n=573)	Women (n=892)	Total (n=1465)	Men (n=356)	Women (n=812)	Total (n=1168)	Men (n=1025)	Women (n=1002)	Total (n=2027)
**Age (yrs)**	51 (45-56)	51 (46-56)	51 (46-56)	50 (45-55)	51 (46-56)	51 (45-55)	49 (44-55)	49 (44-54)	49 (44-54)
**Highest level of education attained**									
No formal	123 (21.5)	280 (31.4)	403 (27.5)*	22 (6.2)	74 (9.1)	96 (8.2)	8 (0.8)	2 (0.3)	10 (0.6)*
Primary	235 (41.1)	340 (38.1)	575 (39.3)	113 (31.7)	273 (33.7)	386 (33.1)	117 (11.4)	636 (80.9)	753 (41.6)
Secondary	175 (30.6)	223 (25.0)	398 (27.2)	204 (57.3)	440 (54.2)	644 (55.2)	749 (73.0)	147 (18.7)	895 (49.4)
Tertiary	39 (6.8)	49 (5.5)	88 (6.0)	17 (4.8)	24 (3.0)	41 (3.5)	152 (14.8)	1 (0.1)	153 (8.4)
**Employment**									
Unemployed	326 (62.3)	558 (64.8)	884 (63.9)	193 (54.7)	532 (65.6)	725 (62.3)*	355 (34.6)	455 (45.4)	810 (40.0)*
Employed	197 (37.7)	303 (35.2)	500 (36.1)	160 (45.3)	279 (34.4)	439 (37.7)	670 (65.4)	547 (54.6)	1217 (60.0)
**Partnership status**									
Never married or co-habited	75 (13.1)	59 (6.3)	134 (9.2)*	103 (28.9)	185 (22.8)	288 (24.7)	265 (25.9)	51 (7.7)	316 (18.7)*
Married or co-habiting	445 (77.1)	537 (60.2)	982 (67.0)	178 (50.0	427 (52.6)	605 (51.8)	570 (55.7)	266 (40.1)	836 (49.5)
Divorced, separated, partner deceased	53 (9.3)	296 (33.2)	349 (23.8)	75 (21.1)	200 (24.6)	275 (23.5)	189 (18.5)	347 (52.3)	536 (31.8)
**SES site quintile**	3 (2-4)	4 (2-5)	3 (2-5)*	3 (2-4)	4 (2-5)	4 (2-5)*	4 (3-5)	2 (2-3)	3 (2-5)*
**BMI (kg/m^2)**	23 (20-27)	28.6 (24-33)	26 (22-31) *	20.6 (19-24)	30.1 (25-36)	26.9 (21-33)*	24.2 (20-28)	32.9 (29-38)	28.4 (23-34)*
**Smoking**									
Never smoker	291 (51.0)	882 (98.8)	1173(80.1)*	54 (15.2)	757 (93.3)	811 (69.6)*	311 (30.4)	893 (89.9)	1204(59.7)*
Current smoker	155 (27.1)	3 (0.3)	158 (10.8)	225 (63.4)	25 (3.1)	250 (21.4)	540 (52.8)	49 (4.9)	589 (29.2)

For peer review only

	Agincourt, SA			Dikgale, SA			Soweto, SA		
	Men (n=573)	Women (n=892)	Total (n=1465)	Men (n=356)	Women (n=812)	Total (n=1168)	Men (n=1025)	Women (n=1002)	Total (n=2027)
Previous smoker	125 (21.9)	7 (0.8)	132 (9.0)	76 (21.4)	29 (3.6)	105 (9.0)	172 (16.8)	51 (5.1)	223 (11.1)
**Alcohol status**									
Never consumed alcohol	187 (32.6)	731 (82.0)	918 (62.7)*	55 (15.5)	561 (69.3)	616 (52.9)*			
Current non-problematic consumer	218 (38.1)	52 (5.8)	270 (18.4)	77 (21.6)	45 (5.6)	122 (10.5)	726 (100.0)		726 (100.0)
Current problematic consumer	18 (3.1)	2 (0.2)	20 (1.4)	142 (39.9)	57 (7.1)	199 (17.1)			
Former consumer	150 (26.2)	107 (12.0)	257 (17.5)	82 (23.0)	146 (18.0)	228 (19.9)			
**GPAQ MVPA (mins/wk)/100**	7.2 (2-14.4)	6 (1.8-13.8)	6.3 (1.8-14.1)	11.4 (6.6-25.8)	12.6 (7.2-21)	12.1 (7.2-21.9)	6.6 (2.1-17.4)	2.1 (0.6-8.6)	1.2 (4.15-13.8)*

Continuous data presented as median (IQR) and categorical data as n(%); * = p<0.05 for difference between men and women in respective sites.

For peer review only

Supplementary Table 2

	Nairobi, Kenya			Nanoro, Burkina Faso			Navrongo, Ghana		
	Men (n=886)	Women (n=1056)	Total (n=1942)	Men (n=1045)	Women (n=1039)	Total (n=2084)	Men (n=923)	Women (n=1091)	Total (n=2014)
**Age (yrs)**	48 (44-53)	48 (44-52)	48 (44-53)	50 (44-55)	50 (45-54)	50 (45-55)	50 (46-55)	52 (47-56)	51 (46-56)*
**Highest level of education attained (%)**									
No formal	34 (3.8)	113 (10.7)	147 (7.6)*	758 (72.8)	960 (93.2)	1718 (83.0)*	570 (61.9)	843 (77.6)	1413 (70.4)*
Primary	447 (50.5)	663 (62.8)	1110 (57.2)	181 (17.4)	58 (5.6)	239 (11.5)	206 (22.4)	177 (16.3)	383 (19.1)
Secondary	383 (43.2)	276 (26.1)	659 (34.0)	86 (8.3)	10 (1.0)	96 (4.6)	118 (12.8)	57 (5.3)	175 (8.7)
Tertiary	22 (2.5)	4 (0.4)	26 (1.3)	16 (1.5)	2 (0.2)	18 (0.9)	27 (2.9)	9 (0.8)	36 (1.8)
**Employment (%)**									
Unemployed	23 (2.6)	90 (8.5)	113 (5.8)*	16 (1.5)	5 (.5)	21 (1.0)*	321 (34.9)	429 (39.4)	750 (37.4)*
Employed	860 (97.4)	966 (91.8)	1826 (94.2)	1026 (98.5)	1030 (99.5)	2056 (99.0)	599 (65.1)	659 (60.6)	1258 (62.6)
**Partnership status (%)**									
Never married or co-habited	13 (1.5)	70 (6.6)	83 (4.3)*	14 (1.3)	3 (0.3)	17* (0.8)	15 (1.6)	5 (0.5)	20* (1.0)
Married or co-habiting	808 (91.2)	486 (46.1)	1,294 (66.7)	1021 (97.9)	794 (76.7)	1815 (87.3)	787 (85.4)	694 (63.6)	1481 (73.6)
Divorced, separated, partner deceased	65 (7.3)	499 (47.3)	564 (29.1)	8 (0.8)	238 (23.0)	246 (11.8)	120 (13.0)	392 (35.9)	512 (25.4)
**SES site quintile (median)**	3 (2-5)	3 (2-4)	3 (2-4)*	3 (2-5)	3 (2-4)	3 (2-5)*	3 (2-5)	3 (2-4)	3 (2-4)*
**BMI (median)**	22.2 (20-25)	26.9 (23-32)	24.4 (21-29)*	21.1 (19-23)	19.8 (18-22)	20.4 (19-23)*	20.6 (19-22)	21.4 (20-24)	21 (19-23)*
**Smoking (%)**									
Never smoker	418 (47.2)	975 (92.3)	1393 (71.8)*	779 (74.7)	1033 (99.8)	1812 (87.2)*	332 (36.0)	1052 (96.6)	1384 (68.8)*
Current smoker	208 (23.5)	27 (2.6)	235 (12.1)	142 (13.6)	0.0 (0.0)	142 (6.8)	388 (42.0)	21 (1.9)	409 (20.3)

For peer review only

	Nairobi, Kenya			Nanoro, Burkina Faso			Navrongo, Ghana		
	Men (n=886)	Women (n=1056)	Total (n=1942)	Men (n=1045)	Women (n=1039)	Total (n=2084)	Men (n=923)	Women (n=1091)	Total (n=2014)
Previous smoker	259 (29.3)	54 (5.1)	313 (16.1)	122 (11.7)	2 (0.2)	124 (6.0)	203 (22.0)	16 (1.5)	219 (10.9)
**Alcohol status (%)**									
Never consumed alcohol	257 (29.0)	758 (71.8)	1015 (52.3)*	273 (26.2)	277 (26.7)	550 (26.5)*	71 (7.7)	231 (21.2)	302 (15.0)*
Current non-problematic consumer	174 (19.6)	32 (3.0)	206 (10.6)	544 (52.3)	520 (50.2)	1064 (51.2)	252 (27.4)	426 (39.1)	678 (33.7)
Current problematic consumer	126 (14.2)	30 (2.8)	156 (8.0)	160 (15.4)	100 (9.67	260 (12.5)	461 (50.0)	167 (15.3)	628 (31.3)
Former consumer	329(37.1)	236 (22.4)	565 (29.1)	64 (6.1)	139 (13.4)	203 (9.8)	137 (14.9)	265 (24.3)	402 (20.0)
**GPAQ MVPA (mins/wk)/100**	12.6 (5.4-31.8)	8.4 (3.9-21.6)	10.5 (4.2-26)*	21.6 (1.2-30.6)	29.4 (5.7-36)	25.2 (3.6-33.6)*	24 (9-30.6)	16.2 (3-26.1)	19.8 (4.5-28.8)*

Continuous data presented as median (IQR) and categorical data as n(%); * = p<0.05 for difference between men and women in respective sites.

Supplementary Table 3

	Agincourt: SA			Dikgale: SA			Soweto, SA		
	Men (n=573)	Women (n=892)	Total (n=1465)	Men (n=356)	Women (n=812)	Total (n=1168)	Men (n=1025)	Women (n=1002)	Total (n=2027)
HIV	190 (33.2)	315 (35.3)	505 (34.5)	73 (19.0)	178 (22.2)	251 (21.9)	198 (20.5)	121 (20.5)	319 (21.1)
Asthma	65 (12.8)	58 (8.0)	123 (10.0)	22 (6.6)	33 (4.2)	55 (4.9)	36 (3.6)		36 (3.6)
Diabetes	55 (9.6)	84 (9.4)	139 (9.5)	33 (9.3)	105 (12.9)	138 (11.8)	89 (8.7)	190 (19.0)	279 (13.8)
Chronic kidney disease	76 (13.9)	145 (17.4)	221 (16.0)	30 (9.4)	113 (15.5)	143 (13.6)	129 (13.4)	-	129 (13.4)
Dyslipidaemia	334 (58.3)	740 (83.0)	1074 (73.3)	208 (58.4)	710 (87.4)	918 (78.6)	639 (62.3)	772 (77.1)	1,411 (69.6)
Hypertension	248 (43.3)	513 (57.5)	761 (52.0)	114 (43.2)	391 (48.1)	505 (43.2)	539 (52.6)	540 (53.9)	1079 (53.2)
Cardiovascular disease	22 (3.8)	46 (5.2)	68 (4.6)	21 (5.9)	59 (7.3)	80 (6.9)	36 (3.5)		36 (3.5)
Number of conditions									
0	62 (10.8)	38 (4.3)	100 (6.8) *	72 (20.2)	36 (4.7)	108 (9.3) *	131 (12.8)	71 (7.1)	202 (10.0) *
1	196 (34.2)	193 (21.6)	389 (26.5)	133 (37.4)	243 (29.9)	376 (32.2)	351 (34.2)	368 (36.7)	719 (35.5)
2	192 (33.5)	354 (39.7)	546 (37.3)	102 (28.6)	323 (39.8)	425 (36.4)	358 (34.9)	440 (43.9)	798 (39.4)
3	89 (15.5)	236 (26.5)	325 (22.2)	36 (10.1)	148 (18.2)	184 (15.8)	143 (14.0)	117 (11.7)	260 (12.8)
4	27 (4.7)	63 (7.1)	90 (6.1)	9 (2.5)	55 (6.8)	64 (5.5)	40 (3.9)	6 (0.6)	46 (2.3)
5	7 (1.2)	8 (0.9)	15 (1.0)	4 (1.1)	6 (0.7)	10 (0.9)	2 (0.2)	-	2 (0.1)
6	0	0	0	0	1 (0.1)	1 (0.1)	0	-	0
Multimorbidity	315 (54.9)	661 (74.1) *	976 (66.6)	151 (42.4)	533 (65.5) *	684 (58.6)	543 (53)	563 (56.2) *	1106 (54.6)

Data presented as n(%); * = p<0.05 for difference between men and women in respective sites

For peer review only

Supplementary Table 4

	Nairobi, Kenya			Nanoro, Burkina Faso			Navrongo, Ghana		
	Men (n=886)	Women (n=1056)	Total (n=1942)	Men (n=1045)	Women (n=1039)	Total (n=2084)	Men (n=923)	Women (n=1091)	Total (n=2014)
HIV	67 (8.9)	172 (17.7)	239 (13.8)	5 (0.5)	4 (0.4)	9 (0.4)	9 (1.0)	7 (0.6)	16 (0.8)
Asthma	20 (2.3)	41 (4.0)	61 (3.2)	19 (2.1)	23 (2.2)	42 (2.2)	3 (0.3)	8 (0.7)	11 (0.5)
Diabetes	44 (5.0)	104 (9.2)	148 (7.6)	65 (6.2)	32 (3.1)	97 (4.6)	42 (4.6)	48 (4.4)	90 (4.5)
Chronic kidney disease	94 (11.5)	138 (14.4)	232 (13.1)	73 (7.4)	81 (7.9)	154 (7.7)	78 (9.0)	87 (8.5)	165 (8.7)
Dyslipidaemia	631 (71.2)	909 (86.1)	1540(79.3)	643 (61.5)	889 (85.6)	1,532 (73.5)	394 (42.7)	861 (78.9)	1,255 (62.3)
Hypertension	199 (22.5)	314 (29.7)	513 (26.4)	211 (20.2)	124 (11.9)	335 (16.1)	223 (24.2)	265 (24.3)	488 (24.2)
Cardiovascular disease	28 (3.2)	55 (5.2)	83 (4.3)	15 (1.4)	15 (1.4)	31 (1.5)	23 (2.5)	28 (2.6)	51 (2.5)
Number of conditions									
0	156 (17.6)	64 (6.1)	220 (11.3) *	295 (28.2)	113 (10.9)	408 (19.6) *	345 (37.4)	147 (13.5)	492 (24.4) *
1	453 (51.1)	481 (45.6)	934 (48.1)	518 (49.6)	716 (68.9)	1234 (59.2)	412 (44.6)	640 (58.7)	1052 (52.2)
2	209 (23.6)	336 (31.8)	545 (28.1)	188 (18.0)	183 (17.6)	371 (17.8)	139 (15.1)	252 (23.1)	391 (19.4)
3	60 (6.8)	130 (12.3)	190 (9.8)	39 (3.7)	22 (2.1)	61 (2.9)	26 (2.8)	49 (4.5)	75 (3.7)
4	8 (0.9)	38 (3.6)	16 (0.8)	5 (0.5)	4 (0.4)	9 (0.4)	1 (0.1)	2 (0.2)	3 (0.1)
5	0	4 (0.4)	4 (0.2)	0	1 (0.1)	1 (0.1)	0	1 (0.1)	1 (0.1)
6	0	3 (0.3)	3 (0.2)	0	0	0	0	0	0
Multimorbidity	277 (31.3)	511 (48.4) *	758 (39)	232 (22.2)	210 (20.2) *	442 (21.2)	166 (18)	304 (27.9) *	470 (23.3)

Data presented as n(%); * = p<0.05 for difference between men and women in respective site

For peer review only - http://bmjopen.bmj.com/site/about/guidelines.xhtml

For peer review only

Supplementary figure 1

a

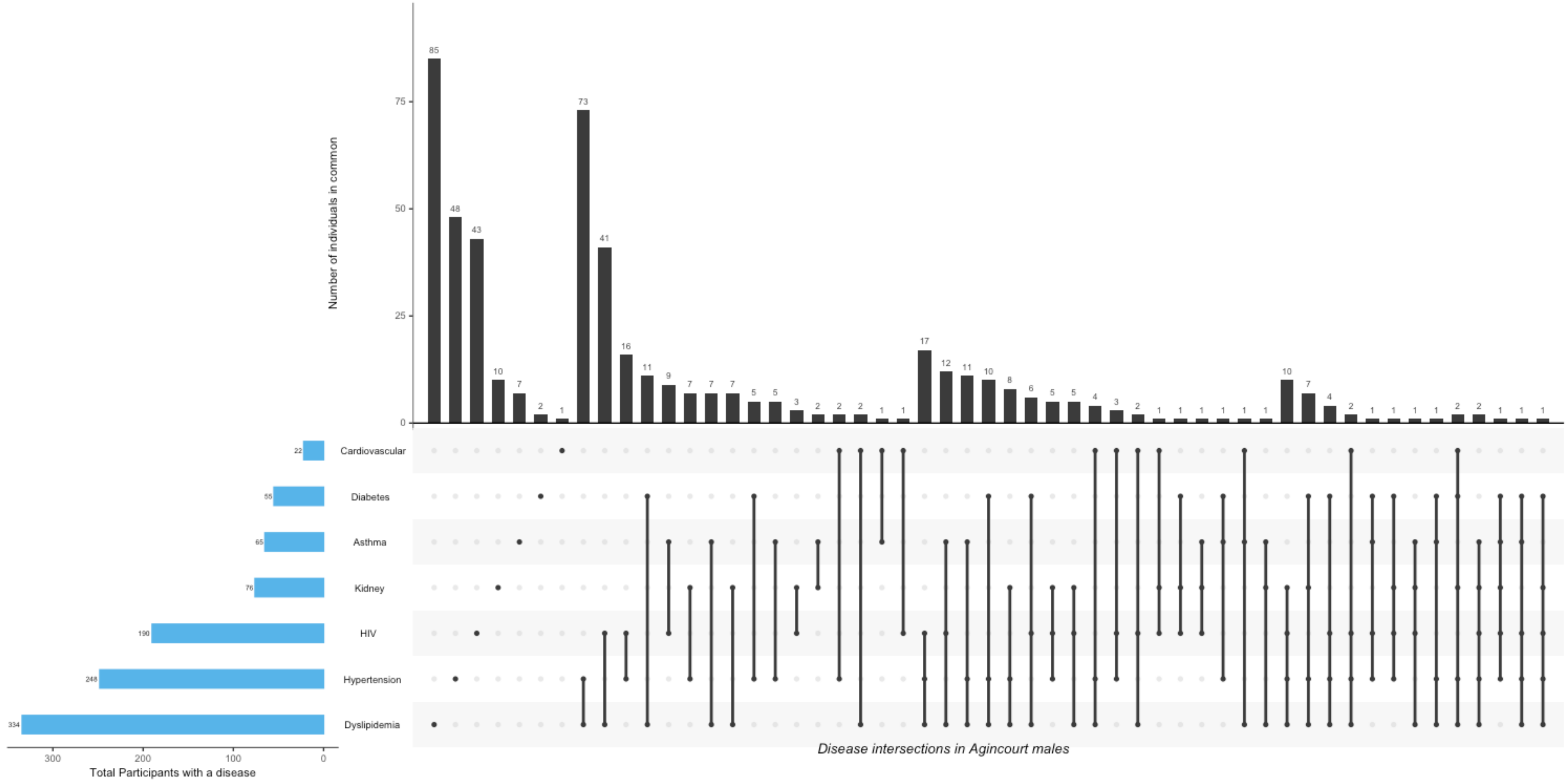

Supplementary figure 2

b

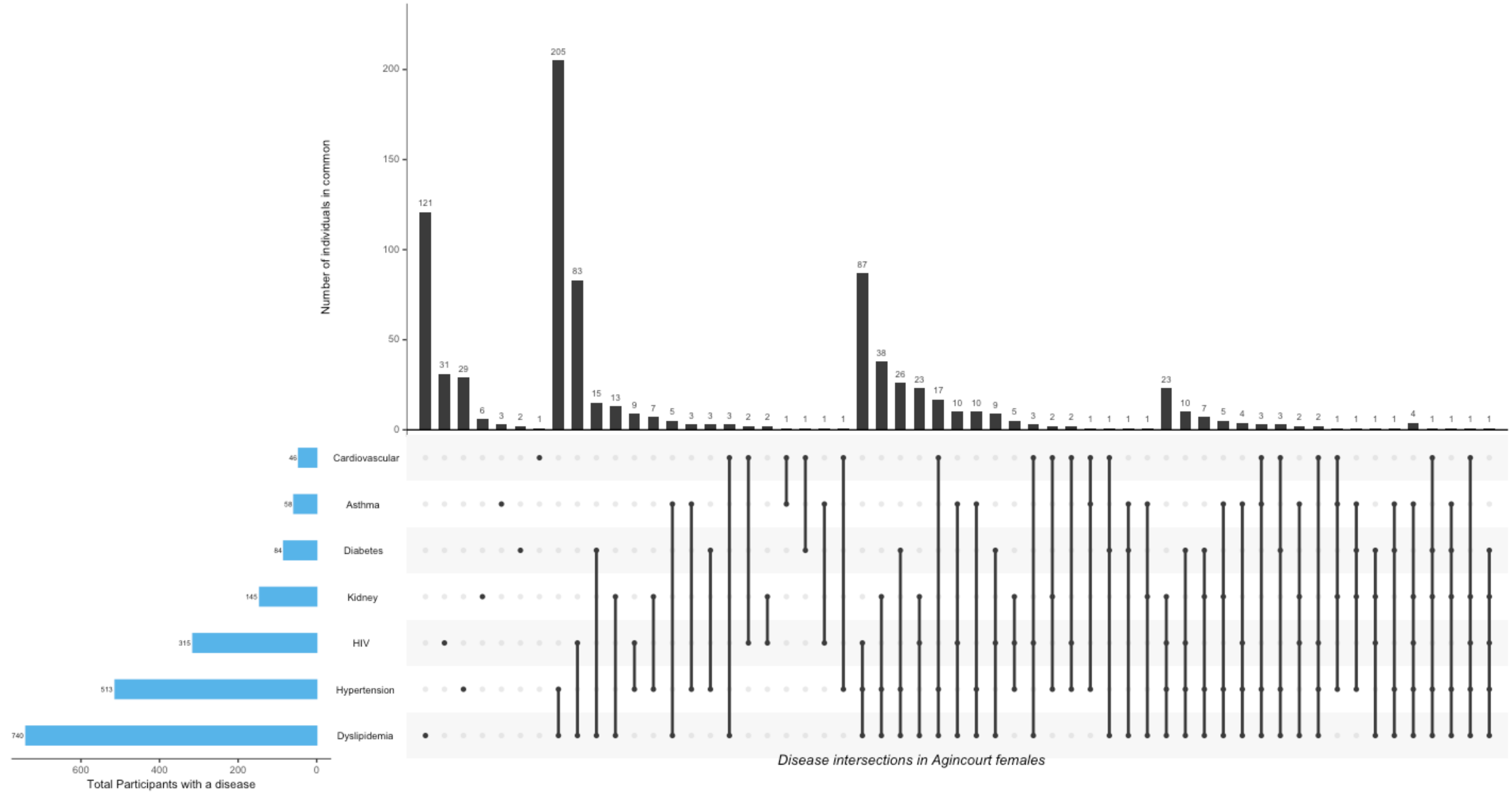

Supplementary figure 3

c

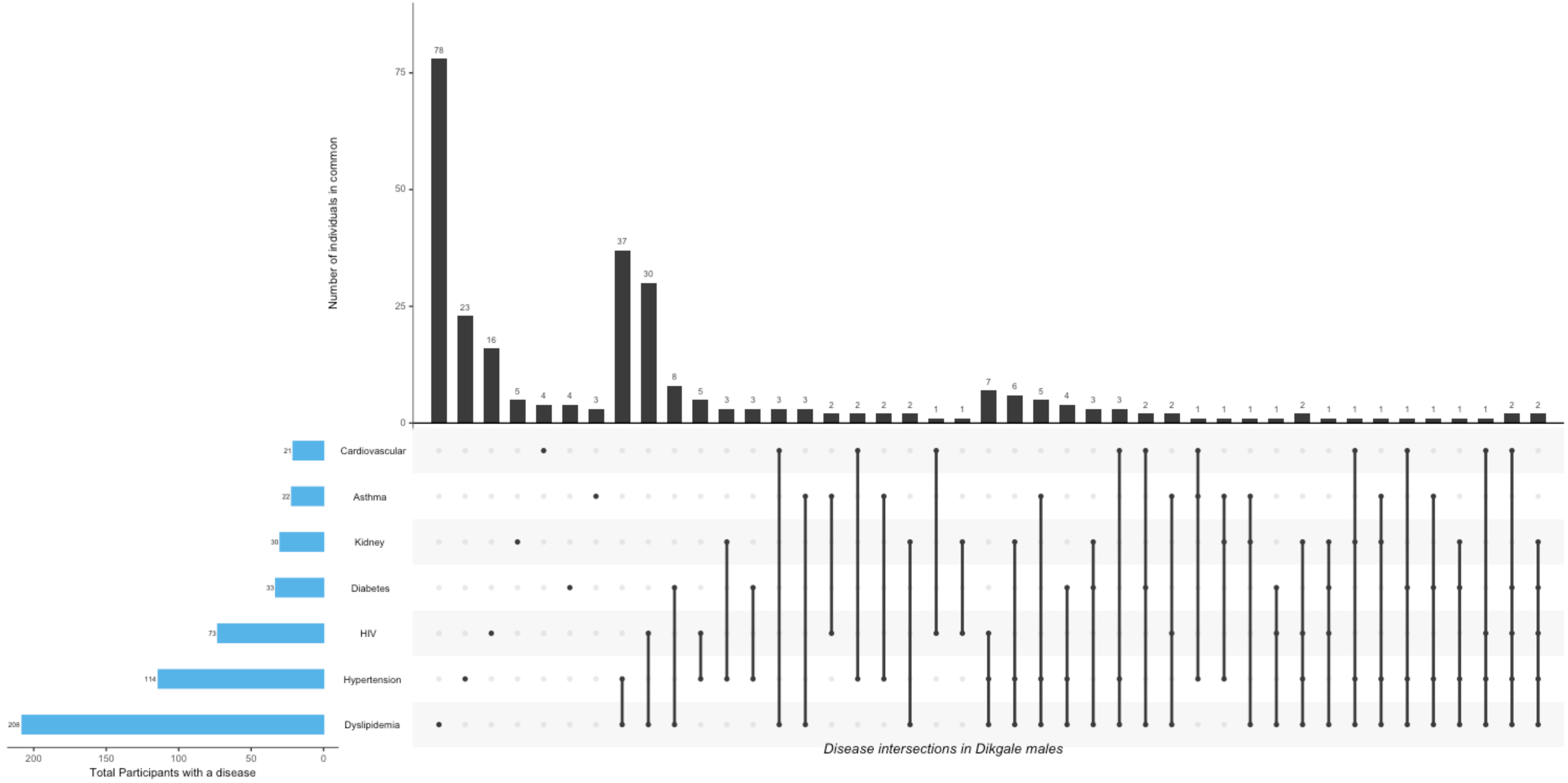

Supplementary figure 4

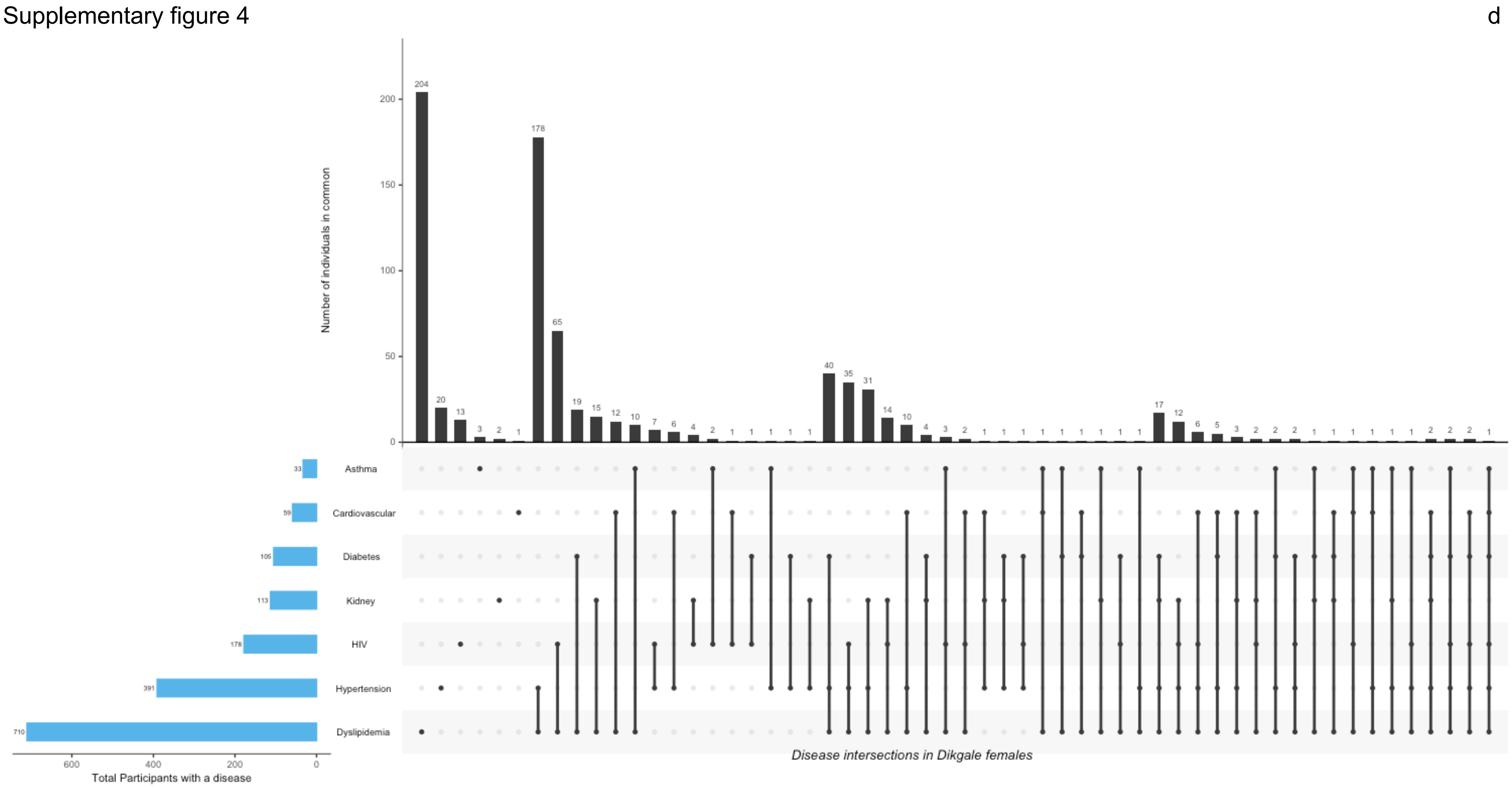

Supplementary figure 5

e

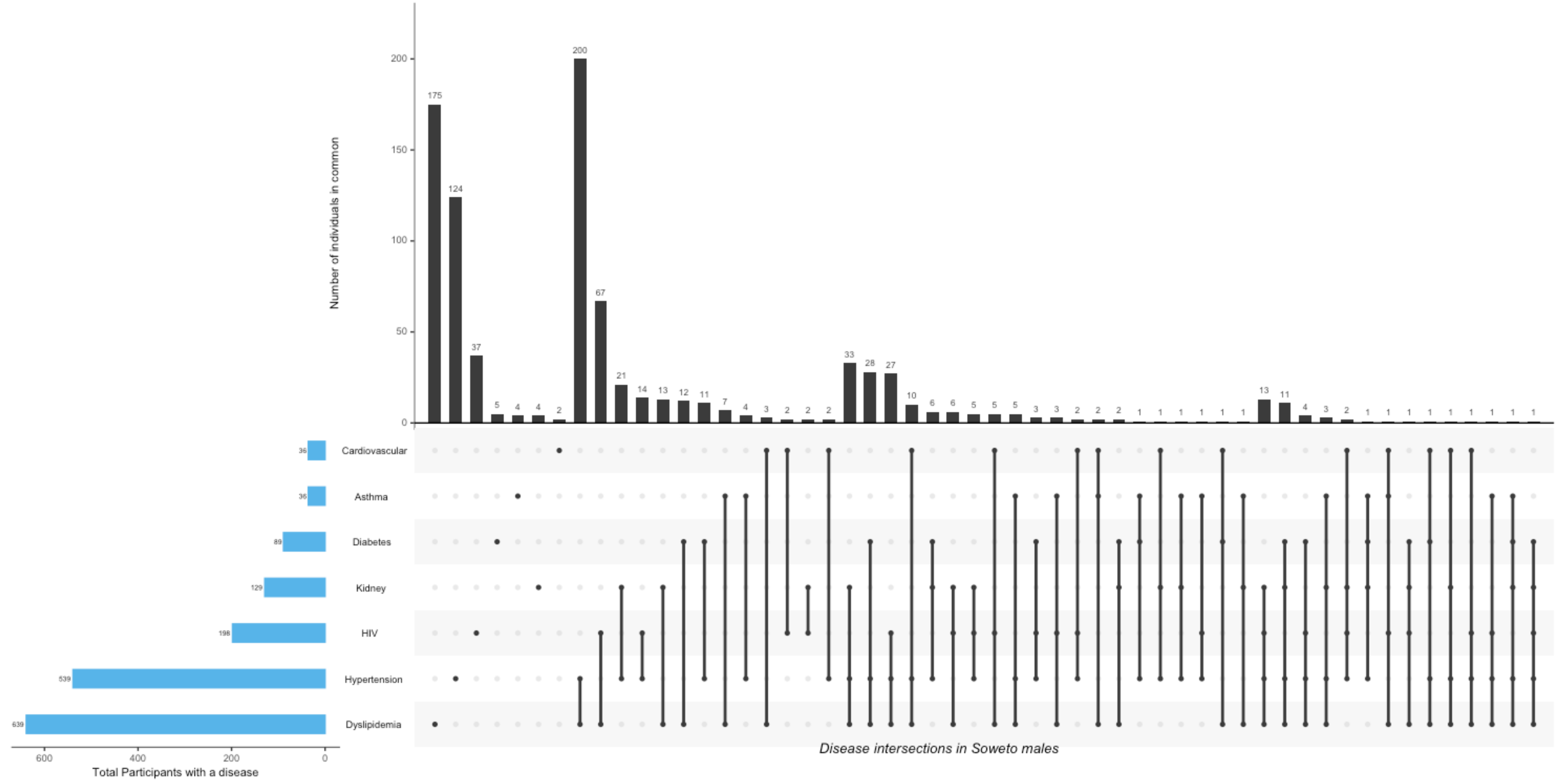

Supplementary figure 6

f

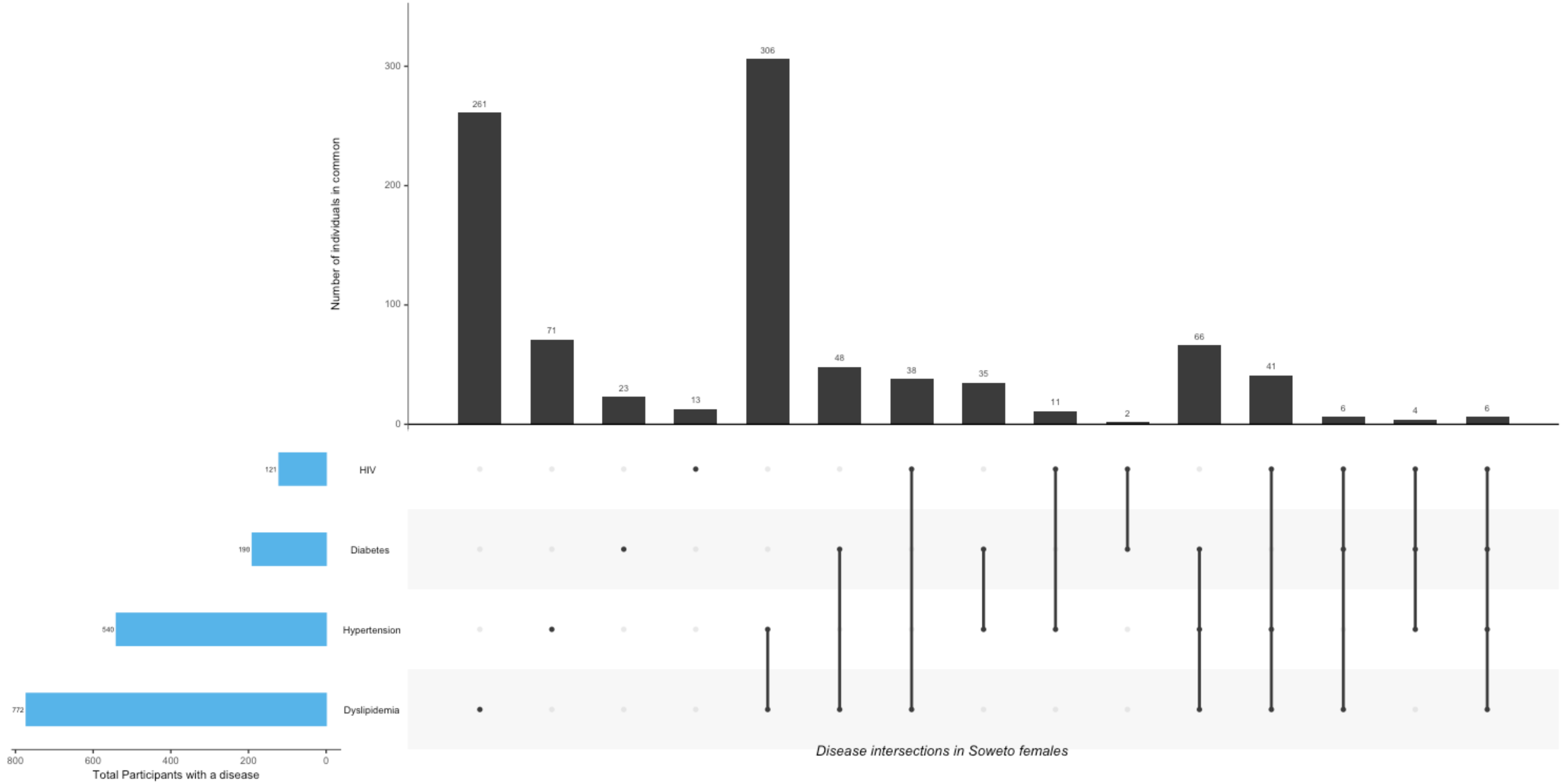

Supplementary figure 7

g

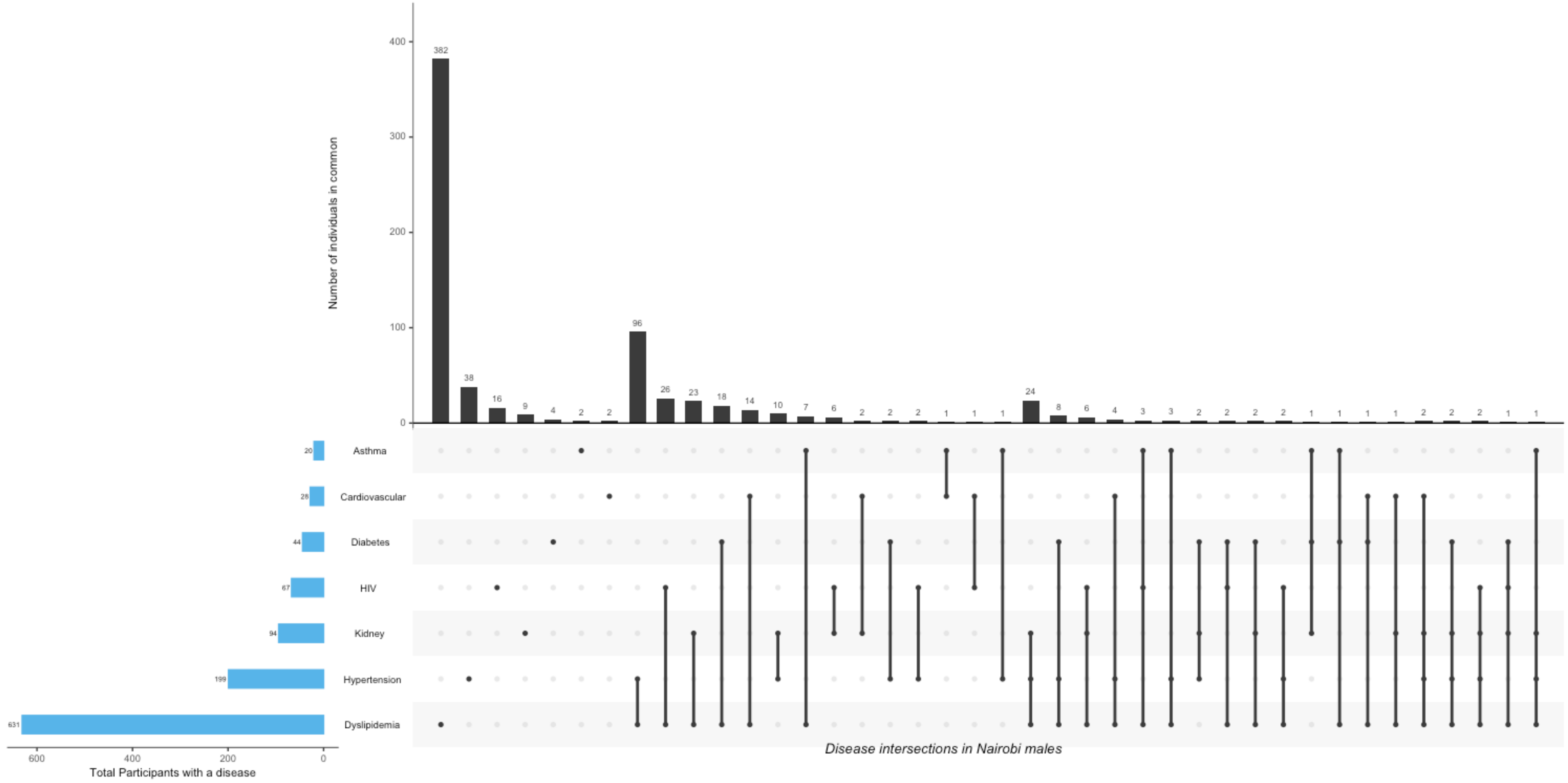

Supplementary figure 8

h

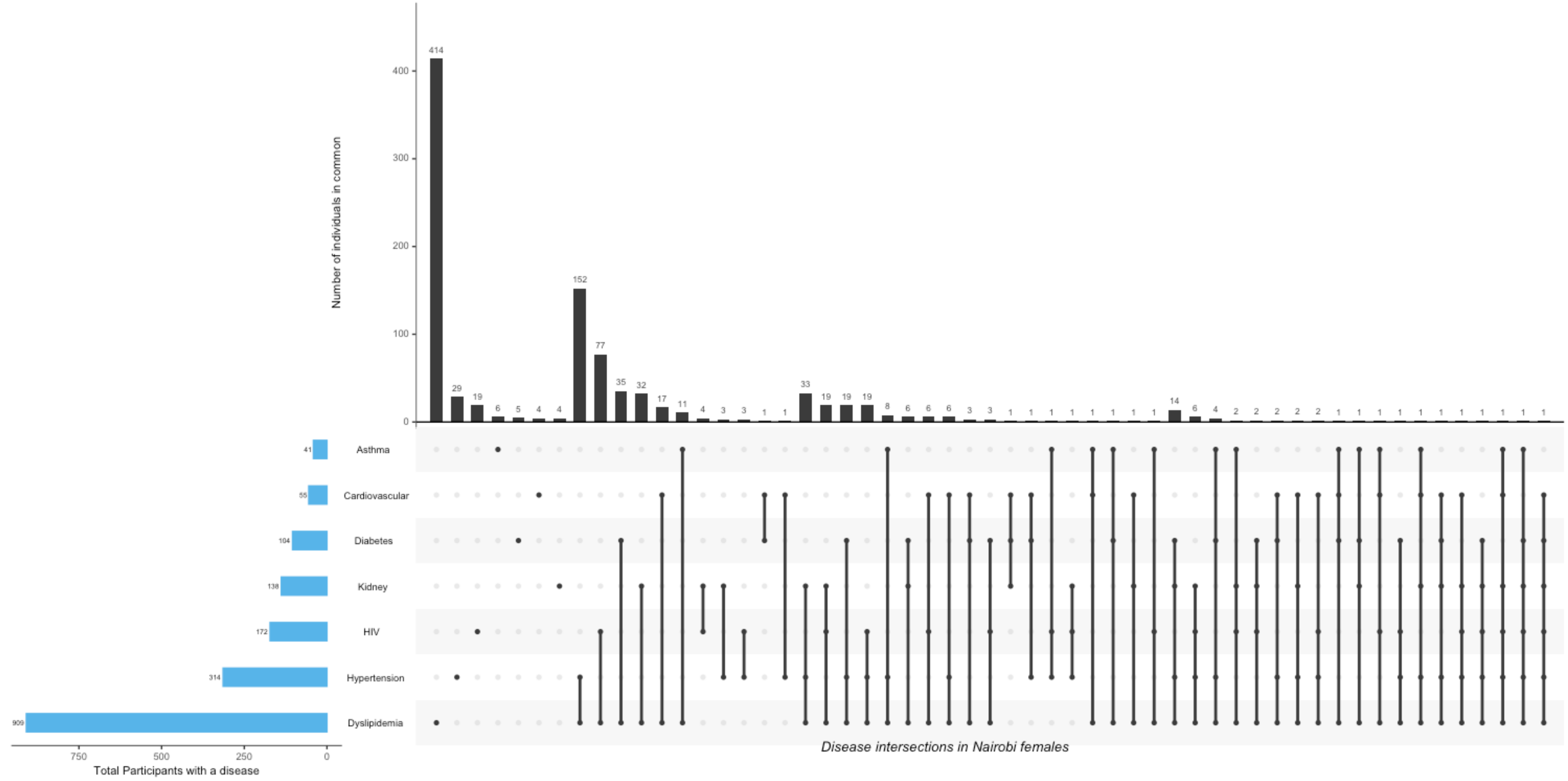

Supplementary figure 9

i

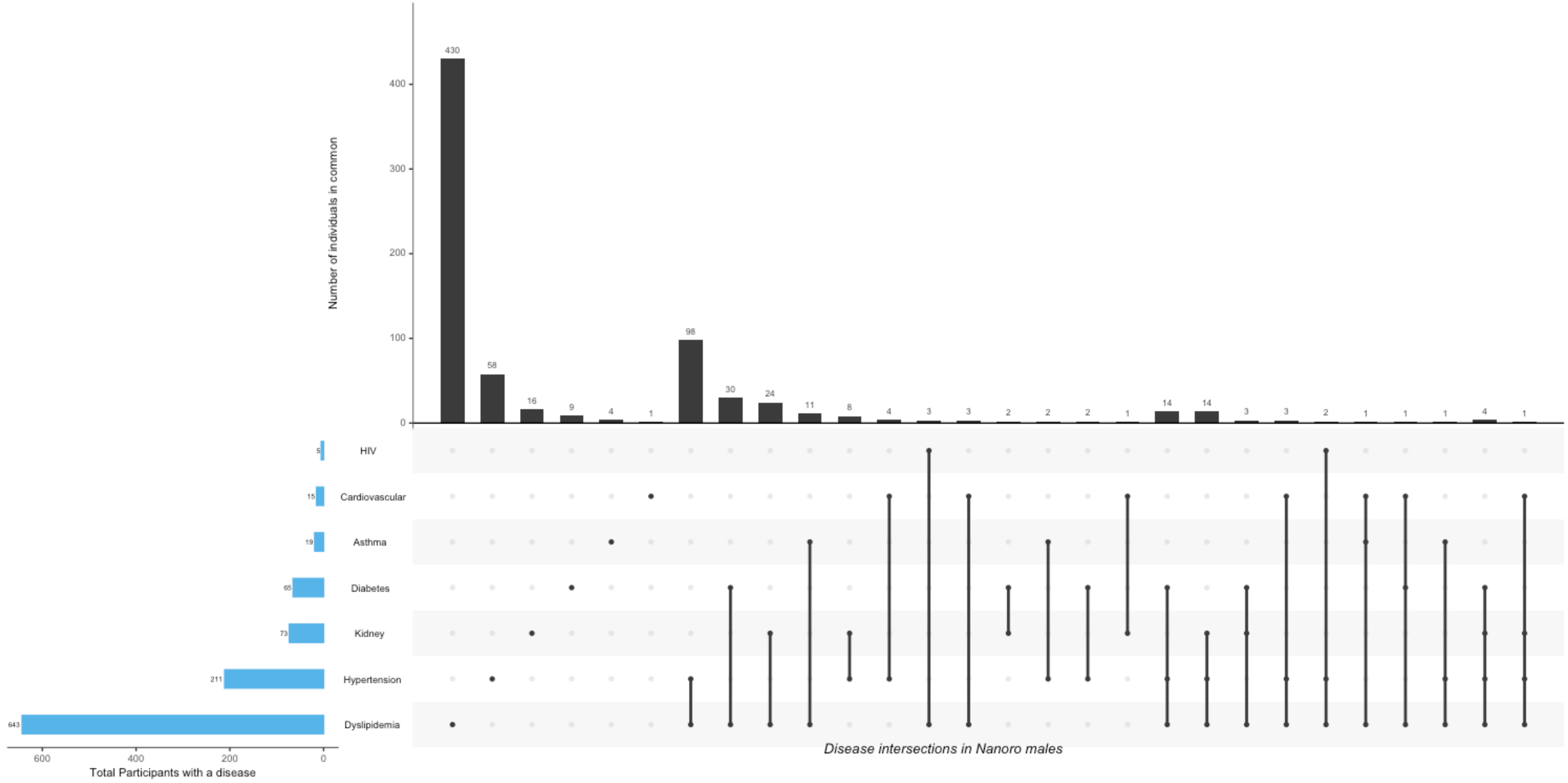

Supplementary figure 10

j

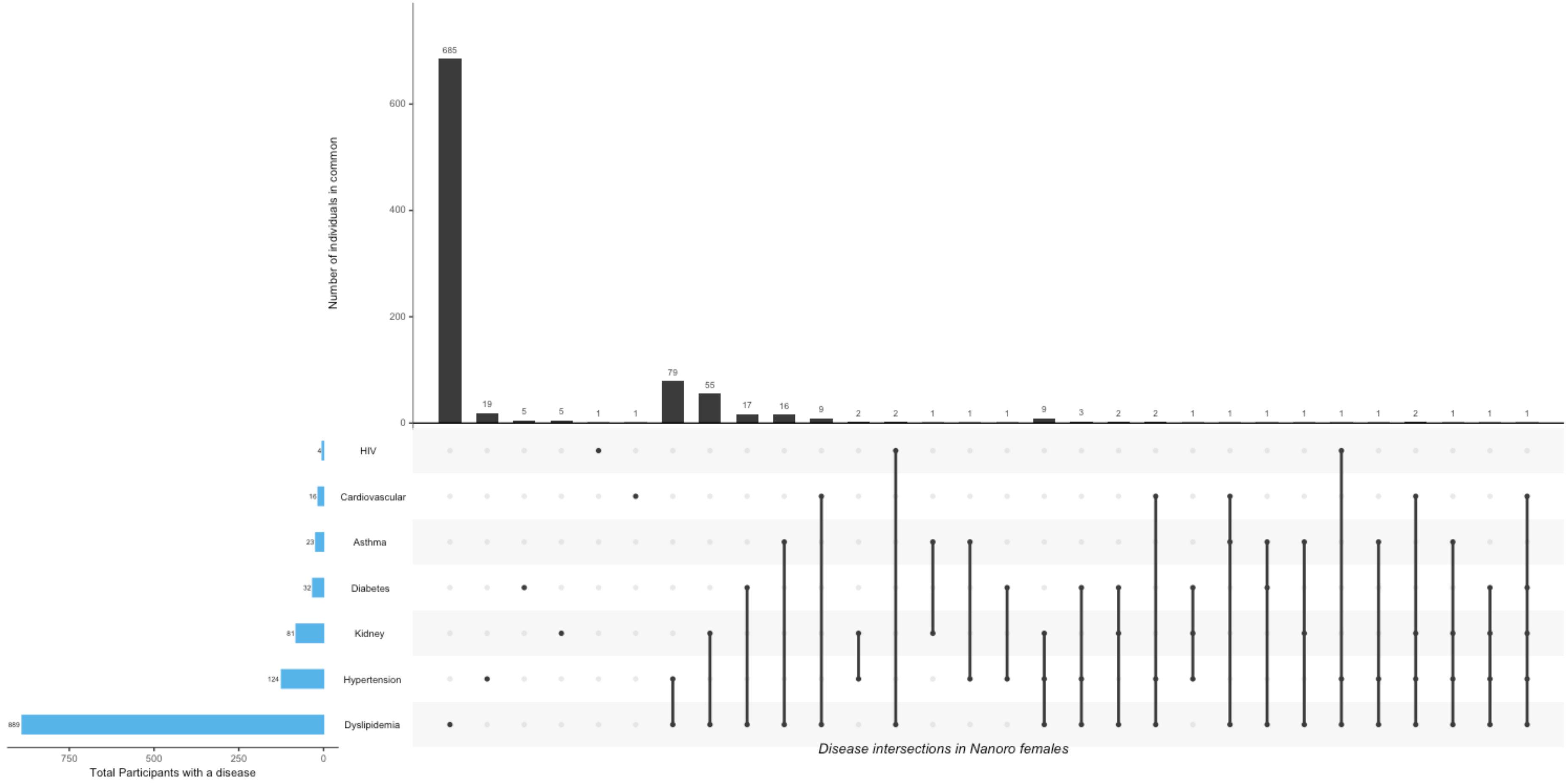

Supplementary figure 11

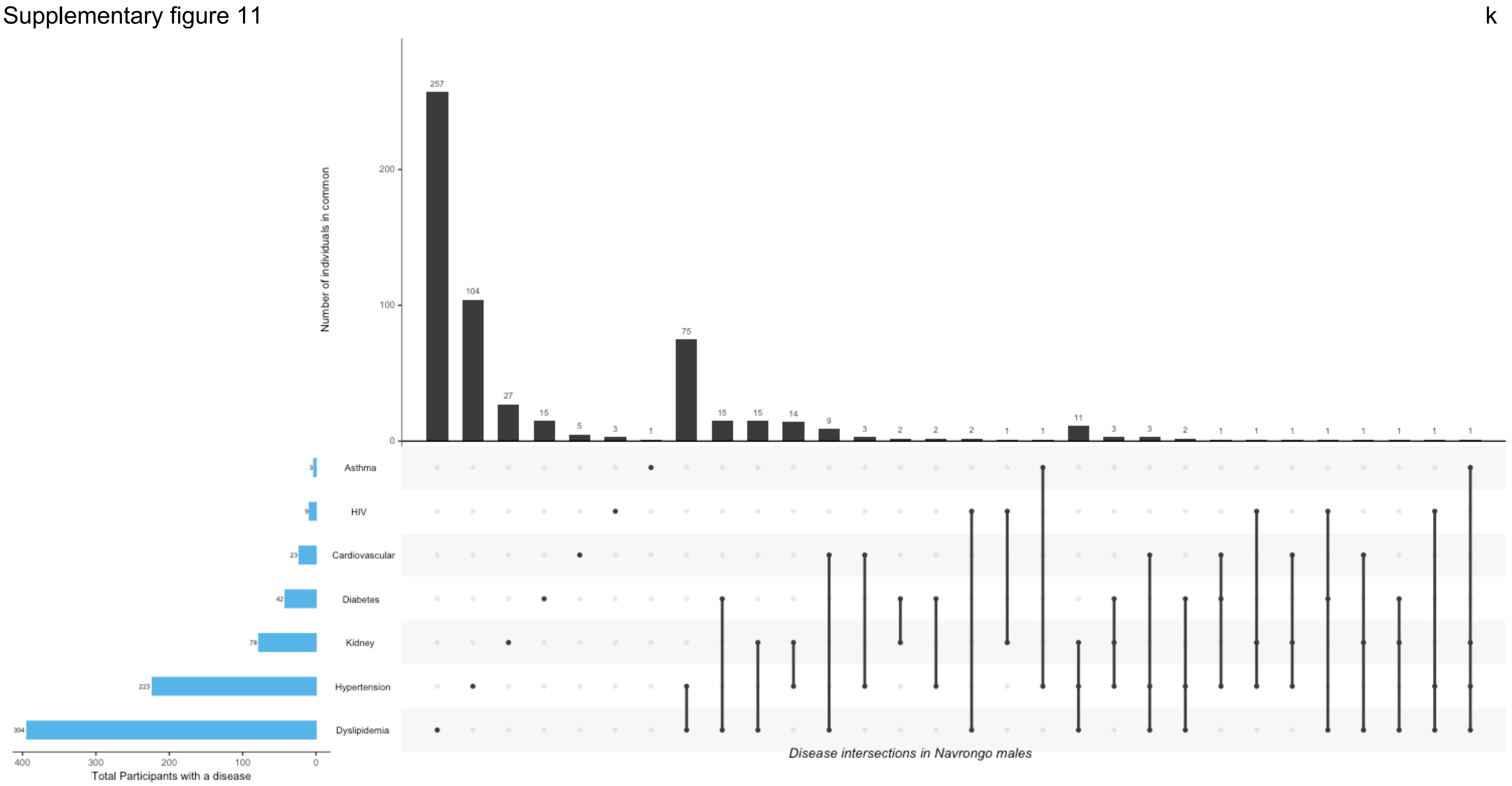

Supplementary figure 12

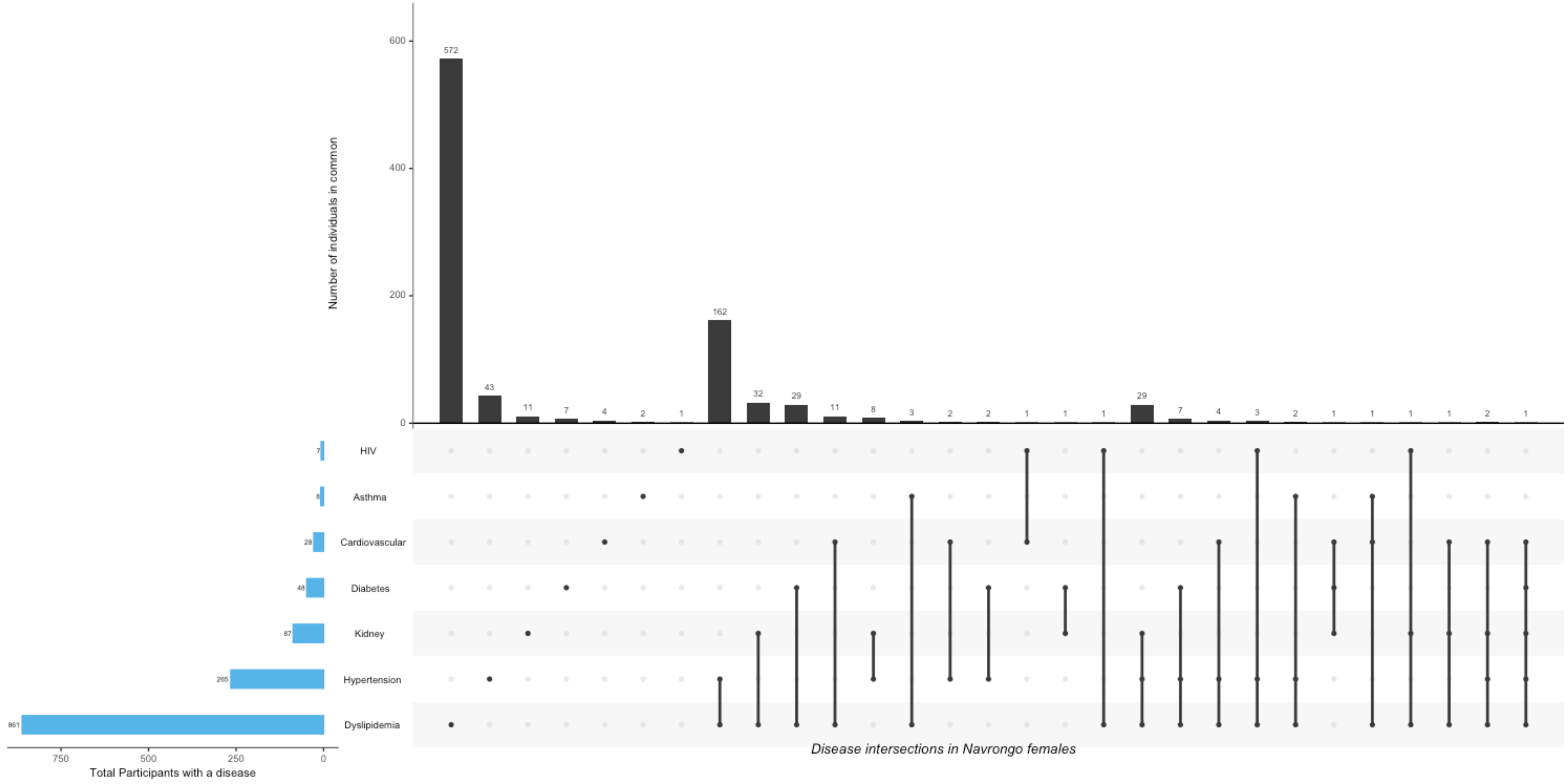

Reporting checklist for cross sectional study.

Based on the STROBE cross sectional guidelines.

Instructions to authors

Complete this checklist by entering the page numbers from your manuscript where readers will find each of the items listed below.

Your article may not currently address all the items on the checklist. Please modify your text to include the missing information. If you are certain that an item does not apply, please write "n/a" and provide a short explanation.

Upload your completed checklist as an extra file when you submit to a journal.

In your methods section, say that you used the STROBE cross sectionalreporting guidelines, and cite them as:

von Elm E, Altman DG, Egger M, Pocock SJ, Gotzsche PC, Vandenbroucke JP. The Strengthening the Reporting of Observational Studies in Epidemiology (STROBE) Statement: guidelines for reporting observational studies.

		Reporting Item	Page Number
**Title and abstract**			
Title	#1a	Indicate the study's design with a commonly used term in the title or the abstract	1
Abstract	#1b	Provide in the abstract an informative and balanced summary of what was done and what was found	3
**Introduction**			
Background / rationale	#2	Explain the scientific background and rationale for the investigation being reported	5
Objectives	#3	State specific objectives, including any prespecified hypotheses	6
**Methods**			
Study design	#4	Present key elements of study design early in the paper	7
Setting	#5	Describe the setting, locations, and relevant dates, including periods of	7

For peer review only

		recruitment, exposure, follow-up, and data collection	
Eligibility criteria	#6a	Give the eligibility criteria, and the sources and methods of selection of participants.	7
	#7	Clearly define all outcomes, exposures, predictors, potential confounders, and effect modifiers. Give diagnostic criteria, if applicable	7-9
Data sources / measurement	#8	For each variable of interest give sources of data and details of methods of assessment (measurement). Describe comparability of assessment methods if there is more than one group. Give information separately for for exposed and unexposed groups if applicable.	7-9
Bias	#9	Describe any efforts to address potential sources of bias	7
Study size	#10	Explain how the study size was arrived at	7
Quantitative variables	#11	Explain how quantitative variables were handled in the analyses. If applicable, describe which groupings were chosen, and why	9
Statistical methods	#12a	Describe all statistical methods, including those used to control for confounding	9
Statistical methods	#12b	Describe any methods used to examine subgroups and interactions	9
Statistical methods	#12c	Explain how missing data were addressed	9
Statistical methods	#12d	If applicable, describe analytical methods taking account of sampling strategy	9
Statistical methods	#12e	Describe any sensitivity analyses	n/a
**Results**			
Participants	#13a	Report numbers of individuals at each stage of study—eg numbers potentially eligible, examined for eligibility, confirmed eligible, included in the study, completing follow-up, and analysed. Give information separately for for exposed and unexposed groups if applicable.	10
Participants	#13b	Give reasons for non-participation at each stage	n/a
Participants	#13c	Consider use of a flow diagram	n/a

For peer review only

Descriptive data	#14a	Give characteristics of study participants (eg demographic, clinical, social) and information on exposures and potential confounders. Give information separately for exposed and unexposed groups if applicable.	10
Descriptive data	#14b	Indicate number of participants with missing data for each variable of interest	n/a
Outcome data	#15	Report numbers of outcome events or summary measures. Give information separately for exposed and unexposed groups if applicable.	10-11
Main results	#16a	Give unadjusted estimates and, if applicable, confounder-adjusted estimates and their precision (eg, 95% confidence interval). Make clear which confounders were adjusted for and why they were included	n/a
Main results	#16b	Report category boundaries when continuous variables were categorized	10-12
Main results	#16c	If relevant, consider translating estimates of relative risk into absolute risk for a meaningful time period	n/a
Other analyses	#17	Report other analyses done—e.g., analyses of subgroups and interactions, and sensitivity analyses	n/a
**Discussion**			
Key results	#18	Summarise key results with reference to study objectives	13
Limitations	#19	Discuss limitations of the study, taking into account sources of potential bias or imprecision. Discuss both direction and magnitude of any potential bias.	16
Interpretation	#20	Give a cautious overall interpretation considering objectives, limitations, multiplicity of analyses, results from similar studies, and other relevant evidence.	13-16
Generalisability	#21	Discuss the generalisability (external validity) of the study results	16
**Other Information**			
Funding	#22	Give the source of funding and the role of the funders for the present study and, if applicable, for the original study on which the present article is based	24

The STROBE checklist is distributed under the terms of the Creative Commons Attribution License CC-BY.
This checklist was completed on 23. August 2022 using https://www.goodreports.org/, a tool made by the EQUATOR Network in collaboration with Penelope.ai

For peer review only

BMJ Open

Identifying the prevalence and correlates of multimorbidity in middle-aged men and women: a cross-sectional population-based study in four African countries

Journal:	*BMJ Open*
Manuscript ID	bmjopen-2022-067788.R1
Article Type:	Original research
Date Submitted by the Author:	07-Dec-2022

Complete List of Authors:	Micklesfield, Lisa; University of the Witwatersrand Johannesburg Faculty of Health Sciences, SAMRC/Wits Developmental Pathways for Health Research Unit, Department of Paediatrics<br>Munthali, Richard; University of the Witwatersrand Johannesburg Faculty of Health Sciences, SAMRC/Wits Developmental Pathways for Health Research Unit, Department of Paediatrics<br>Agongo, Godfred; C K Tedam University of Technology and Applied Sciences, Department of Biochemistry and Forensic Sciences; Ghana Health Service, Navrongo Health Research Centre<br>Asiki, G; African Population and Health Research Center<br>Boua, Palwende; Institut de Recherche en Sciences de la Santé, Clinical Research Unit of Nanoro; University of the Witwatersrand Johannesburg, Sydney Brenner Institute of Molecular Bioscience<br>Choma, Solomon SR; University of Limpopo, Department of Pathology<br>Crowther, Nigel; University of the Witwatersrand Johannesburg Faculty of Health Sciences, Department of Chemical Pathology<br>Fabian, June; University of the Witwatersrand Johannesburg, Wits Donald Gordon Medical Centre<br>Gómez-Olivé, F. Xavier; University of the Witwatersrand Johannesburg Faculty of Health Sciences, Medical Research Council/Wits Rural Health and Health Transitions Unit (Agincourt), School of Public Health, Faculty of Health Sciences<br>Kabudula, Chodziwadziwa; University of the Witwatersrand School of Public Health, MRC/Wits Rural Public Health and Health Transitions Research Unit (Agincourt),<br>Maimela, Eric; University of Limpopo, Department of Pathology<br>Mohamed, Shukri; African Population and Health Research Center, Health and Systems for Health<br>Nonterah, Engelbert ; Ghana Health Service, Navongo Health Research Centre; Utrecht University, Julius Global Health, Julius Centre for Health Sciences and Primary Care<br>Raal, Frederick; University of the Witwatersrand Johannesburg Faculty of Health Sciences, Department of Medicine<br>Sorgho, Hermann; Institut de Recherche en Sciences de la Santé<br>Tluway, Furahini; University of the Witwatersrand Johannesburg Faculty of Health Sciences, Sydney Brenner Institute for Molecular Bioscience<br>Wade, Alisha N.; University of the Witwatersrand Johannesburg Faculty of Health Sciences, 9MRC/Wits Rural Public Health and Health Transitions Research Unit, School of Public Health

	Norris, Shane; University of the Witwatersrand Faculty of Health Sciences, SAMRC/Wits Developmental Pathways for Health Research Unit, Department of Paediatrics<br>Ramsay, Michele; University of the Witwatersrand Johannesburg Faculty of Health Sciences, Sydney Brenner Institute for Molecular Bioscience

**Primary Subject Heading**:	Epidemiology
Secondary Subject Heading:	Epidemiology, Global health
Keywords:	EPIDEMIOLOGY, PUBLIC HEALTH, Lipid disorders < DIABETES & ENDOCRINOLOGY

SCHOLARONE™
Manuscripts

I, the Submitting Author has the right to grant and does grant on behalf of all authors of the Work (as defined in the below author licence), an exclusive licence and/or a non-exclusive licence for contributions from authors who are: i) UK Crown employees; ii) where BMJ has agreed a CC-BY licence shall apply, and/or iii) in accordance with the terms applicable for US Federal Government officers or employees acting as part of their official duties; on a worldwide, perpetual, irrevocable, royalty-free basis to BMJ Publishing Group Ltd ("BMJ") its licensees and where the relevant Journal is co-owned by BMJ to the co-owners of the Journal, to publish the Work in this journal and any other BMJ products and to exploit all rights, as set out in our licence.

The Submitting Author accepts and understands that any supply made under these terms is made by BMJ to the Submitting Author unless you are acting as an employee on behalf of your employer or a postgraduate student of an affiliated institution which is paying any applicable article publishing charge ("APC") for Open Access articles. Where the Submitting Author wishes to make the Work available on an Open Access basis (and intends to pay the relevant APC), the terms of reuse of such Open Access shall be governed by a Creative Commons licence – details of these licences and which Creative Commons licence will apply to this Work are set out in our licence referred to above.

Other than as permitted in any relevant BMJ Author's Self Archiving Policies, I confirm this Work has not been accepted for publication elsewhere, is not being considered for publication elsewhere and does not duplicate material already published. I confirm all authors consent to publication of this Work and authorise the granting of this licence.

For peer review only

Identifying the prevalence and correlates of multimorbidity in middle-aged men and women: a cross-sectional population-based study in four African countries

Running title: Multimorbidity in African adults

Lisa K. Micklesfield[1], Richard Munthali[1], Godfred Agongo[2,3], Gershim Asiki[4], Palwende Romuald Boua[5,12], Solomon SR Choma[6], Nigel J. Crowther[7], June Fabian[8], F. Xavier Gomez-Olive[9], Chodziwadziwa W Kabudula[9], Eric Maimela[6], Shukri F Mohamed[4], Engelbert A. Nonterah[3,10], Frederick Raal[11], Hermann Sorgho[5], Furahini D. Tluway[12], Alisha N. Wade[9], Shane A. Norris[1], Michèle Ramsay[12] as members of AWI-Gen and the H3Africa Consortium.

[1]South African Medical Research Council/Wits Developmental Pathways for Health Research Unit, Department of Paediatrics, Faculty of Health Sciences, University of the Witwatersrand, Johannesburg, South Africa

[2]Department of Biochemistry and Forensic Sciences, C.K. Tedam University of Technology and Applied Sciences, Navrongo, Ghana

[3]Navrongo Health Research Centre, Ghana Health Service, Navrongo, Ghana

[4]African Population and Health Research Center, Nairobi

[5]Clinical Research Unit of Nanoro, Institut de Recherche en Sciences de la Santé, Burkina Faso

[6]DIMAMO Health and Population Research Centre, Department of Pathology, Faculty of Health Sciences University of Limpopo

[7]Department of Chemical Pathology, National Health Laboratory Service, University of the Witwatersrand Faculty of Health Sciences, Johannesburg

[8]Wits Donald Gordon Medical Centre, School of Clinical Medicine, Faculty of Health Sciences, 7 York Rd, Parktown, 2193, Johannesburg, South Africa

[9]MRC/Wits Rural Public Health and Health Transitions Research Unit, School of Public Health, Faculty of Health Sciences, University of the Witwatersrand, 27 St. Andrew's Road, Parktown, Johannesburg 2193, South Africa

[10]Julius Global Health, Julius Centre for Health Sciences and Primary Care, University Medical Centre Utrecht, Utrecht University, Utrecht, the Netherlands

[11]Carbohydrate & Lipid Metabolism Research Unit, Division of Endocrinology & Metabolism, Department of Medicine, Faculty of Health Sciences, University of the Witwatersrand, Parktown, Johannesburg, South Africa

For peer review only

[12]Sydney Brenner Institute for Molecular Bioscience, Faculty of Health Sciences, University of the Witwatersrand, Johannesburg, South Africa

[#]**Corresponding author**: Professor Lisa Micklesfield, SAMRC/Wits Developmental Pathways for Health Research Unit, University of the Witwatersrand, Johannesburg, South Africa. Email: lisa.micklesfield@wits.ac.za; Tel: 0027 (0)11 9331122.

Key words: multimorbidity, Africa, chronic disease, prevalence, disease risk

Word count: 4594

For peer review only

Abstract

Objectives: To determine the prevalence of multimorbidity, to identify which chronic conditions cluster together, and to identify factors associated with a greater risk for multimorbidity in sub-Saharan Africa.

Design: Cross-sectional, multicentre, population-based study

Setting: Six urban and rural communities in four sub-Saharan African countries.

Participants: Men (n=4808) and women (n=5892) between the ages of 40 and 60 years from the AWI-Gen study.

Measures: Sociodemographic and anthropometric data, and multimorbidity as defined by the presence of two or more of the following conditions: HIV infection, cardiovascular disease, chronic kidney disease, asthma, diabetes, dyslipidaemia, hypertension.

Results: Multimorbidity prevalence was higher in women compared to men (47.2 vs 35%), and higher in South African men and women compared to their East and West African counterparts. The most common disease combination at all sites was dyslipidaemia and hypertension, with this combination being more prevalent in South African women than any single disease (25 vs 21.6%). Age and BMI were associated with a higher risk of multimorbidity in men and women, however lifestyle correlates such as smoking and physical activity were different between the sexes.

Conclusions: The high prevalence of multimorbidity in middle-aged adults in sub-Saharan Africa is of concern, with women currently at higher risk. This prevalence is expected to increase in men, as well as in the East and West African region with the ongoing epidemiological transition. Identifying common disease clusters and correlates of multimorbidity is critical to providing effective interventions.

For peer review only

Strengths and limitations of this study

- A strength of the study is that it provides multimorbidity prevalence data on a large (n=11 273), population-based sample of middle-aged men and women from sub-Saharan Africa.
- Study sites include urban and rural communities from South, East and West Africa.
- A limitation is the cross-sectional study design with some of the diseases measured via self-report with no longitudinal follow-up.
- The cross-sectional study design only allows for the identification of correlates of multimorbidity, rather than causal factors.

For peer review only

Introduction

Sub-Saharan Africa (SSA) is experiencing the highest rate of urbanisation globally and together with an increase in life expectancy must prepare for the prevalence of non-communicable diseases (NCDs) to increase further (1). Multimorbidity, the co-occurrence of two or more chronic diseases in one individual, is common, challenging the affected individual, their attending healthcare professionals, and an overstretched health system. A recent systematic review highlighted a paucity of multimorbidity costing studies from low- and middle-income countries (2). When describing global patterns of multimorbidity Afshar et al., reported a positive but non-linear association between country GDP and multimorbidity prevalence, and also identified an inverse association between multimorbidity and SES in countries with the highest GDP, with the gradient sometimes reversed in countries with lower GDP(3). Understanding the social and structural forces that drive the clustering of diseases thereby exacerbating their impact on disease outcomes, is defined as syndemics(4), and is critical to our understanding of multimorbidity.

Recent data from low- and middle-income countries (LMICs), including South Africa, have been reported in a scoping review of NCD multimorbidity, which ranged in prevalence from 3.2% to 90.5%, and reported that 95.3% of the studies found female sex to be a risk factor for multimorbidity (5). The studies included in this review used various chronic diseases comprising the multimorbidity profile, and different diagnostic criteria for each chronic disease, highlighting the need for a consensus on the definition of multimorbidity and the core conditions that it is comprised of. Further the inclusion of chronic infectious diseases such as HIV and tuberculosis should also be considered when quantifying the burden of multimorbidity but may be more relevant in some settings than others. There is a dearth of multimorbidity prevalence data from Africa, with studies reporting data from single countries such as South Africa (6,7), Ghana (8), Burkina Faso (9), and Kenya (10). Although the influence of age and socioeconomic status on multimorbidity prevalence are well recognised globally (3,5,11,12), several African studies have also identified associations between lifestyle factors, mental health and multimorbidity (7,10,12). Comparing and contrasting different African settings and identifying which diseases cluster together and the factors associated with this clustering, will assist in the design of effective interventions.

The double burden of non-communicable and infectious diseases as well as the impact of urbanisation on disease risk, highlights the need to undertake multimorbidity research in Africa,

For peer review only

and the heterogeneity in the epidemiological and nutrition transition, and disease burden of different African countries, provides an opportunity to compare and contrast the correlates and prevalence of multimorbidity across different African settings. The aim of this study was to determine the prevalence of multimorbidity, to identify which chronic conditions cluster together, and to identify factors associated with a greater risk for multimorbidity in six rural and urban communities in four SSA countries. This study will contribute to the evidence-base on multimorbidity by providing prevalence data across different African settings and identifying common disease clusters to potentially understand the pathogenesis of different diseases. This will inform the design of more effective interventions and provide formative data on multimorbidity for policymakers in the planning and implementation of more effective health systems with particular relevance to other low- and middle- income countries.

For peer review only

Methods

Study population

Data included in this study are from the Africa Wits-INDEPTH (University of the Witwatersrand, Johannesburg, and the International Network for the Demographic Evaluation of Populations and Their Health) partnership for Genomic Studies (AWI-Gen) (13), which is a National Institutes of Health funded Collaborative Centre of the Human Heredity and Health in Africa (H3Africa) Consortium. AWI-Gen is a population-based cross-sectional study of adults and includes six participating urban and rural centres in four SSA countries. West Africa included two countries, Ghana (Navrongo) and Burkina Faso (Nanoro); East Africa included Kenya (Nairobi); and South Africa had three study sites (Soweto, Agincourt and Dikgale). Details of the recruitment strategy as well as other data collected are described in Ali et al.(14). For this study we only included participants from 40 to 60 years (n=11 273).

Socio-demographic and anthropometric data

Standard structured AWI-Gen questionnaires were administered by trained field staff with some country and site-specific modifications to suit their context (14). Data on self-reported socio-demographics (age, highest level of education attained, partnership status, employment status) were collected. Each study participant was assigned to a socio-economic status (SES) quintile which was determined for each study site by categorizing factor scores that were predicted from a principal component analysis of the number of household assets. Alcohol consumption was categorised as never, current problematic, current non-problematic or former consumer (15), but was not available for the Soweto women. Smoking of tobacco products was categorised as never, current or previous smoker. Total moderate-vigorous intensity physical activity (MVPA) was calculated as minutes per week from the accumulation of occupation, walking for travel and leisure time activity collected by self-report using the Global Physical Activity Questionnaire (GPAQ) (16). Weight and height were used to calculate body mass index (BMI: weight (kg)/height (m^2)).

Multimorbidity

We defined multimorbidity (MM) as the presence of two or more of the following seven conditions (HIV infection, cardiovascular disease, chronic kidney disease (CKD), asthma, diabetes, dyslipidemia and hypertension) for all sites except for women from Soweto, where data was only available for four of the conditions (HIV infection, diabetes, dyslipidemia and hypertension).

For peer review only - http://bmjopen.bmj.com/site/about/guidelines.xhtml

Obesity was not included as one of the conditions as in the context of this paper it is considered an NCD risk factor rather than a disease. For this reason, BMI was included as an independent variable in the multinomial regression.

HIV status was self-reported although participants in South Africa and Kenya were offered a voluntary government-approved rapid HIV test. Due to the low prevalence of HIV in Burkina Faso and Ghana participants were not offered HIV tests.

Hypertension was defined as systolic blood pressure equal or greater than 140 mm Hg and/or diastolic blood pressure equal or greater than 90 mm Hg, in line with the seventh report of the Joint National Committee on Prevention, Detection, Evaluation, and Treatment of High Blood Pressure, or if the participant was taking hypertension medication(17).

Diabetes was defined using the World Health Organisation criteria which are the presence of a previous diagnosis of diabetes by a healthcare professional or fasting blood glucose >=7 mmol/L or random glucose >=11.1 mmol/L, or on diabetes medication at the time of recruitment (18).

Dyslipidemia was defined as the presence of one of the following: total cholesterol (TC) ≥5.0mmol/L, or high-density lipoprotein cholesterol (HDL-C) <1.0mmol/L for men and <1.3mmol/L for women, or low-density lipoprotein cholesterol (LDL-C) ≥3. 0mmol/L, or triglycerides (TG) ≥1.7 mmol/L, or on lipid-modifying medication (19), or self-report of ever being diagnosed by a health professional with high cholesterol. The Randox Daytona Plus (Randox Laboratories Ltd, UK) autoanalyzer was used to analyze HDL-C, TG and TC on fasting venous blood samples. Low density lipoprotein cholesterol (LDL-C) was calculated using the Friedewald equation (20).

Chronic kidney disease was defined as estimated glomerular filtration rate (eGFR) <60 mL/min per 1.73 m^2 (calculated using the CKD-EPI (creatinine) equation 2009, without adjustment for African American ethnicity), presence of albuminuria (urine albumin creatinine ratio >3 mg/mmol) or both(21). As the study was cross-sectional, low EGFR and albuminuria were not confirmed with follow up testing. At each partner site, blood and urine specimens were processed and stored at -112°F. After completion of the study each partner site transported samples on dry ice to a central laboratory in Johannesburg, South Africa. At the central laboratory all analyses were batched and performed according to good laboratory practice with external monitoring for quality control. Serum and urine creatinine (mg/dL) were measured using

For peer review only

Jaffe's kinetic method calibrated to an isotope dilution mass spectrometry (IDMS)-traceable standard. Urinary albumin concentration was measured with immunoturbidimetry. The urine albumin to urine creatinine ratio (ACR; mg/mmol) was calculated from these measurements.

Cardiovascular disease (CVD) was defined as present if the participant reported having had a heart attack or stroke or transient ischemic attack (TIA). Participants previously diagnosed with congestive heart failure (CHF) or angina were also classified as having CVD. No data were available for angina or heart attack prevalence for the Soweto men so cardiovascular disease prevalence were calculated using the remaining data.

Asthma was determined by self-report or use of medication for the condition.

Statistical analysis

Data were summarized using means and standard deviations (±SD) for continuous parametric data, and median (interquartile range) for non-parametric data. A Students t-test was used to test for differences between men and women on parametric continuous variables within each site while Kruskal–Wallis test was used for non-parametric data. Chi-square test was used for categorical data. Due to significant sex differences within sites (Supplementary Tables 1-4) all further analyses were stratified by sex. Since the outcome had three categories, zero conditions, one condition, and at least two conditions (multimorbidity) of the seven conditions under study, multinomial logistic regression was used to explore the factors associated with either one condition or at least two conditions, with none as the reference group, in men and women separately. A *p*-value less than 0.05 was considered statistically significant in all tests carried out using STATA version 14.1 SE (Stata Corporation, College Station, TX).

To understand the different multimorbidity patterns between men and women, within and between countries, and between regions i.e. South (South African sites), East (Kenyan site), and West (Ghana and Burkina Faso) Africa, different diseases were plotted using UpSetR, an R package (22). Separate analyses were done for men and women, separated by site and then geographic region.

Patient and public involvement

This study did not involve any patients and/or the public.

For peer review only

Results

Socio-demographic and lifestyle factors

Only participants between the ages of 40 and 60 years were selected for these analyses (n=11 273), and the median age ranged from 48 years in Nairobi to 51 years in Agincourt, Dikgale and Navrongo (Supplementary tables 1 and 2). The only site where there was a significant sex difference in age was Navrongo where women were older than men. Sex differences in education level attained were significant at all sites, except Dikgale where the majority of participants had completed secondary school (55.2%), while in Navrongo and Nanoro the majority of the participants did not have any formal education. Employment was above 90% in both Nairobi and Nanoro, 62.6% and 60% in Navrongo and Soweto, respectively and only 36.1% in Agincourt and 37.7% in Dikgale. There were significant sex differences in SES at all six sites. Smoking and alcohol consumption were significantly different between the sexes at all sites except Soweto (alcohol status was not known in the women), as previously reported (23).

Multimorbidity prevalence

For the total study population as well as when the sites were combined by geographical area, multimorbidity prevalence was higher in women compared to men, with nearly 50% of the women in the total sample presenting with multimorbidity compared to 35% of the men (Figure 1). Multimorbidity prevalence was highest in the South African men and women (51.7% and 64.9%, respectively), followed by East Africa (31.3% and 48.4%, respectively), and then West Africa (20.2% and 24.1%, respectively). Overall, the site with the highest multimorbidity prevalence was Agincourt with 66.6%, and the site with the lowest prevalence was Nanoro with 21.2% (Supplementary tables 3 and 4).

Multimorbidity clusters

Multimorbidity clustering for the total study population, and for the 3 geographical regions, stratified by sex, are presented in Figures 2 and 3, respectively.

Study population, stratified by sex (Figure 2)

The majority of the total study population of men (42.9%) and women (44.8%) had one disease only, and in those with only two diseases the most common combination was dyslipidemia and hypertension (men 12%; women 18%) (Figure 2a and b). While in men, the most common

For peer review only

combination of three diseases was dyslipidemia, hypertension, and chronic kidney disease (2% of the total study population), in women the most common combination was dyslipidemia, hypertension, and HIV infection (3.2%). The prevalence of four or more diseases was marginally higher in women compared to men (3.3% vs 2.1%) with the most common cluster of four diseases being dyslipidemia, hypertension, HIV, and chronic kidney disease in both sexes.

Geographical regions, stratified by sex (Figure 3)

The majority of men (51.7%) and women (64.9%) living in South Africa presented with multimorbidity (Supplementary Table 2, Figure 1), with two diseases (hypertension and dyslipidemia) (Figure 3a and b) as the most common combination in the men (33.4%) and women (41.3%). In contrast the majority of men (51.1%) and women (45.6%) from East Africa reported only one disease, although the pattern in those with two diseases was similar to South Africa as dyslipidemia and hypertension were the most common cluster (Figures 3c and d), and this was the same in West Africa (Figures 3e and f). It is in the clustering of three or more diseases that there are differences between the sites as the most common clustering of three diseases in South African men and women was dyslipidemia, hypertension and HIV, while in East and West Africa dyslipidemia and hypertension were most commonly clustered with chronic kidney disease.

Sites, stratified by sex

At all sites except Nanoro the prevalence of multimorbidity was higher in women than men, with the prevalence being highest in Agincourt women (74.1%) and lowest in men from Navongo (18%) (Supplementary tables 3 and 4). In all the sites the most common disease combination in men and women with two diseases only was dyslipidemia and hypertension (Supplementary figures 1 to 12), with the lowest prevalence in Nanoro women (7.6%) and the highest prevalence in Soweto women (30.5%). There were differences between the sites in the most common disease clusters in men and women with three diseases only, with dyslipidemia and hypertension clustered with CKD in Soweto men, Nairobi men and women, Navrongo men and women, and Nanoro women. In men from Dikgale, and Agincourt men and women, the most common disease cluster in those with only three diseases was dyslipidemia, hypertension and HIV, while in Dikgale and Soweto women, and Nanoro men, it was dyslipidemia, hypertension and diabetes. The highest prevalence with four or more diseases was reported in women from the South African sites: 8% of the Agincourt women and 7.6% of the Dikgale women (data was

For peer review only

not available for Soweto women). Except for Nairobi women, less than 1% of the men and women from the East and West African sites presented with four or more diseases.

Multinomial logistic regression, stratified by sex

Risk factors associated with having one condition and with having multimorbidity, compared to no condition, were determined using multinomial regression, and are presented for women (Table 1) and men (Table 2).

Table 1: Multinomial logistic regression to determine factors associated with either one condition or multimorbidity, with none as the reference group, in AWI-Gen women

	No disease vs one disease			No disease vs multimorbidity		
	RRR	95% CI	p value	RRR	95% CI	p value
Site (Ref: Agincourt)						
Dikgale	1.42	0.84, 2.43	0.185	0.77	0.46, 1.28	0.306
Nairobi	1.92	1.18, 3.11	0.009	0.58	0.36,0.93	0.023
Nanoro	3.10	1.78, 5.41	0.000	0.38	0.22,0.67	0.001
Navrongo	1.56	0.97, 2.52	0.068	0.25	0.16, 0.41	0.000
Age (yrs)	1.00	0.98, 1.02	0.932	1.04	1.02, 1.06	0.000
BMI (kg/m^2)	1.07	1.04, 1.10	0.000	1.11	1.08, 1.14	0.000
Smoking status (Ref: never smoked)						
Current smoker	0.58	0.27, 1.26	0.171	0.82	0.38, 1.80	0.628
Previous smoker	0.56	0.27, 1.18	0.126	0.76	0.37, 1.58	0.468
Alcohol consumption (Ref: never consumed)						
Current non-problematic consumer	0.86	0.64, 1.16	0.327	0.97	0.70, 1.34	0.851
Current consumer (problematic)	1.01	0.66, 1.55	0.959	1.14	0.72, 1.79	0.580
Former consumer	1.49	1.04, 2.12	0.028	1.97	1.37, 2.84	0.000
GPAQ MVPA (mins/wk/100)	0.98	0.99, 1.01	0.550	0.99	0.99, 1.00	0.132
Partnership status (Ref: never married or co-habited)						
Married or co-habiting	1.40	0.82, 2.4	0.223	1.13	0.66, 1.90	0.658

For peer review only

Divorced, separated, partner deceased	1.62	0.93, 2.82	0.090	1.48	0.86, 2.54	0.157
Highest level of education attained (Ref: no formal education)						
Primary education	1.12	0.80, 1.57	0.505	1.34	0.95, 1.90	0.094
Secondary education	1.31	0.84, 2.03	0.235	1.51	0.97, 2.36	0.067
Tertiary education	2.95	0.67, 12.9	0.152	2.81	0.65, 12.18	0.168
Employment status (Ref: unemployed)						
Employed	0.76	0.57, 1.02	0.069	0.79	0.59, 1.07	0.125
SES quintile (Ref: quintile 1)						
Quintile 2	0.96	0.68, 1.34	0.799	1.16	0.81, 1.66	0.412
Quintile 3	1.27	0.88, 1.83	0.195	1.35	0.92, 1.98	0.127
Quintile 4	0.99	0.70, 1.39	0.945	1.03	0.72, 1.48	0.857
Quintile 5	0.99	0.68, 1.42	0.940	1.15	0.78, 1.68	0.489

Table 2: Multinomial logistic regression to determine factors associated with either one condition or multimorbidity, with none as the reference group, in AWI-Gen men

	No disease vs one disease			No disease vs multimorbidity		
	RRR	95% CI	p value	RRR	95% CI	p value
**Site** (Ref: Agincourt)						
Dikgale	0.75	0.48, 1.17	0.204	0.59	0.38, 0.92	0.019
Nairobi	0.95	0.64, 1.40	0.781	0.42	0.28, 0.63	0.000
Nanoro	0.80	0.54, 1.18	0.256	0.29	0.19, 0.43	0.000
Navrongo	0.55	0.38, 0.80	0.002	0.16	0.11, 0.24	0.000
Soweto	1.15	0.75, 1.78	0.515	1.19	0.77, 1.84	0.422
Age (yrs)	1.01	0.99,1.02	0.369	1.03	1.02, 1.05	0.000
BMI (kg/m^2)	1.09	1.06, 1.12	0.000	1.14	1.11, 1.18	0.000
Smoking status (Ref: never smoked)						
Current smoker	1.07	0.87, 1.32	0.520	0.95	0.75, 1.20	0.671

For peer review only

Previous smoker	1.25	0.99, 1.58	0.061	1.17	0.91, 1.52	0.223
Alcohol consumption (Ref: never consumed)						
Current non-problematic consumer	0.66	0.52, 0.85	0.001	0.68	0.51,0.89	0.006
Current consumer (problematic)	0.63	0.47, 0.83	0.001	0.77	0.56, 1.06	0.105
Former consumer	1.09	0.81, 1.48	0.571	1.10	0.79, 1.54	0.568
GPAQ MVPA (mins/wk/100)	0.99	0.99, 0.10	0.015	0.99	0.99, 0.10	0.004
Partnership status (Ref: never married or co-habited)						
Married or co-habiting	1.39	1.01, 1.92	0.045	1.53	1.10, 2.15	0.011
Divorced, separated, partner deceased	1.61	1.08, 2.40	0.018	2.43	1.62, 3.65	0.000
Highest level of education attained (Ref: no formal education)						
Primary education	1.11	0.89, 1.40	0.355	1.30	1.00, 1.69	0.049
Secondary education	1.10	0.85, 1.44	0.460	1.22	0.91,1.64	0.183
Tertiary education	1.04	0.65, 1.68	0.858	1.10	0.67, 1.82	0.710
Employment status (Ref: unemployed)						
Employed	0.94	0.75, 1.17	0.555	0.77	0.61, 0.98	0.033
SES quintile (Ref: quintile 1)						
Quintile 2	1.09	0.83, 1.43	0.525	1.18	0.87, 1.61	0.293
Quintile 3	1.07	0.81, 1.41	0.630	1.08	0.79, 1.49	0.632
Quintile 4	1.12	0.86, 1.48	0.381	1.11	0.81, 1.51	0.512
Quintile 5	1.03	0.78, 1.36	0.823	1.303	0.96, 1.78	0.093

Due to the lower prevalence of multimorbidity in women from East and West Africa compared to South Africa, women living in Nairobi and Nanoro were more likely to have one condition compared to Agincourt women, and conversely, together with women from Navrongo, they were at a lower relative risk of multimorbidity compared to women from Agincourt. In women, being older was associated with a higher relative risk of multimorbidity, and a 1 kg/m^2 higher BMI was associated with a 7% higher risk of having one condition and an 11% higher risk of multimorbidity. Further, being a former consumer of alcohol compared to never consuming

For peer review only

alcohol, was associated with a higher risk of having one disease, and with multimorbidity. None of the other measures of socio-economic status or lifestyle behaviors were associated with multimorbidity risk in women.

When compared to the Agincourt men the relative risk of multimorbidity was lower at all sites, except Soweto. Similar to the women, older age and BMI were associated with a higher risk of multimorbidity with every year of age being associated with a 3% higher risk of multimorbidity, and a 1 kg/m^2 higher BMI was associated with a 9% higher risk of having one condition, and a 14% higher risk of multimorbidity. While smoking was not associated with disease risk the current consumption of alcohol, whether non-problematic or problematic, was associated with a lower risk of having one disease, and current non-problematic consumption of alcohol was associated with a 32% lower risk of multimorbidity. Another lifestyle behavior that was associated with disease risk was time spent in moderate-vigorous intensity physical activity, with more time spent in MVPA associated with a lower risk of having one condition, and with multimorbidity. Living with a partner either presently or in the past was associated with a higher risk of one condition and an even higher risk of multimorbidity when compared to not living with a partner. The attainment of primary school level education was associated with a 30% higher risk of multimorbidity compared to no formal education, and being employed was associated with a 33% lower risk of having multimorbidity compared to being unemployed, however there was no association between socio-economic status quintile and risk for one disease or multimorbidity.

Discussion

In this study of middle-aged men and women living in South, East and West Africa we have reported sex differences in the prevalence of multimorbidity, and identified socio-demographic, socio-economic and lifestyle factors associated with multimorbidity risk in men and women. While the prevalence of multimorbidity was more than 50% in South Africa, it was only just over 20% in West Africa, with East Africa reporting a prevalence of 31.3% in men and 48.4% in women. What was consistent across all sites was the higher prevalence of multimorbidity in women compared to men, and in both men and women age and BMI were independently associated with a higher risk of multimorbidity. These findings highlight the need for longitudinal studies of aging African populations to examine the effect of multimorbidity on mortality, as studies from high-income countries have shown that this is influenced by disease type, number and certain combinations that comprise multimorbidity(24,25). Our study has also shown that

For peer review only

the most commonly occurring disease cluster at all SSA sites was dyslipidaemia with hypertension.

A recent systematic review of prevalence studies from South Africa reported a 3-23% prevalence of multimorbidity in studies with a wide range of age groups, and 30-71% in older adults (26). The higher prevalence of multimorbidity in the South African sites in the current study (54-66%) may be attributed to the advanced epidemiological transition and increasing urbanisation in South Africa (27). This coincides with a higher BMI and prevalence of obesity in the three South African sites compared to the West and East African sites (28). Despite being considered a rural site, Agincourt reported the highest prevalence of multimorbidity at 66.6%. This prevalence is similar to those reported from the same study site but including larger sample sizes of participants older than 40 years (n>3000) and different chronic diseases in the definition of multimorbidity (29,30). Kabudula et al., (31) have described a 'protracted' epidemiological transition in Agincourt between 1993 and 2013 as a result of the co-existence of infectious and non-communicable diseases, as well as the influence of social changes, and their results highlight the different transition experience in low and middle-income countries compared to high income countries (27). Although the West African sites, Ghana and Burkina Faso, reported the lowest prevalence of multimorbidity and a similar prevalence between men and women, it is expected that this will increase, following a similar trajectory to the South African sites. Mohamed et al., (10) have reported a lower multimorbidity prevalence of 28.7% (31.5% in women vs 21.4% in men) in the same AWI-Gen study participants from Nairobi, Kenya. Reasons for the different prevalence compared to the current study is that they included 16 chronic conditions, and all measurements, except for hypertension and obesity, were via self-report. This may have resulted in an underestimation of multimorbidity prevalence as it is well recognised that many of these chronic diseases are undiagnosed in Africa (32–34). The higher prevalence of multimorbidity in women compared to men has been well described (35), with the studies from South Africa showing less consistent results (26).

Dyslipidaemia was the most commonly occurring disease across all the sites, with the prevalence ranging from 42.7% in men from Navrongo, to 87.4% in women from Dikgale. These results are similar to other African studies (36,37), with a recent study by Masilela et al., (38) reporting a prevalence of 76.7% in South African adults receiving care for diabetes and hypertension, and Reiger et al.,(39) reporting a prevalence of 67.3% in over 4000 adults aged 40 years and older from Agincourt, South Africa. Many of these African studies have reported that low HDL-C is the main driver of the high prevalence of dyslipidaemia, and it is important to

For peer review only

debate whether the current international cut-offs for HDL-C, triglyceride and other cholesterol measures are relevant to the African population. A low HDL-C is frequently seen with obesity and the metabolic syndrome and is associated with hypertriglyceridemia, particularly post-prandially (40). The main driver of atherosclerotic cardiovascular disease is LDL-C so this may be a more important marker of risk for cardiovascular disease (41). If this is the main criterion for defining dyslipidaemia and low HDL-C were excluded from the definition, then the prevalence of dyslipidaemia would be considered to have a lesser effect in the context of multimorbidity. Data from Agincourt has reported that while low HDL (<1.19 mmol/L) was prevalent in 26.5% of the adults aged 40 years and older, high LDL (>4.1 mmol/L) was measured in only 3.7% (42).

In the current study the most common cluster of two diseases at all sites was dyslipidaemia with hypertension, with this prevalence being higher than the prevalence of any single disease in Agincourt women, and Soweto men and women. This is in contrast to a recent systematic review from South Africa which identified the common disease clusters as hypertension and diabetes, hypertension and HIV, and TB and HIV (26). Irrespective of the difference in findings these studies highlight the high prevalence of hypertension in these large samples of middle-aged men and women from East, West and South Africa as previously reported by Gomez-Olive et al (34). A recent systematic review and meta-analysis assessing chronic kidney disease in African participants with hypertension provides strong evidence for the devastating outcomes of untreated hypertension as it reports a 17.8% pooled prevalence of chronic kidney disease in people with hypertension(43). In the total sample of men in the current study the most prevalent combination of three diseases was dyslipidaemia, hypertension and kidney disease (2% of the total sample) while in the women this was dyslipidaemia, hypertension and HIV (3% of the total sample). The co-occurrence of kidney disease and hypertension was more common than kidney disease and diabetes in both men and women, a finding which has previously been reported in Africa (44). Several multimorbidity patterns including a ‘cardio-respiratory’ pattern and a ‘metabolic’ pattern were identified across countries in the SAGE study reporting data on 12 chronic conditions, although the factors associated with multimorbidity differed between countries and could be explained by their diverse development status (11). Reporting on the South African data only Chidumwa et al., identified three groups using latent class analysis (7). While 88% of the sample were classified as minimal multimorbidity risk, 11% were classified as concordant (hypertension and diabetes) multimorbidity and 6% as discordant (angina, asthma, chronic lung disease, arthritis and depression) multimorbidity(7).

For peer review only

Identifying factors associated with multimorbidity clustering is important in designing country or region-specific strategies to manage multimorbidity. Results from this study as well as global and African studies have reported an increased risk of multimorbidity with increasing age (10,30). Although this association is well accepted and understood, in the study by Garin et al., (11)multimorbidity prevalence decreased with age in the South African cohort, which they suggeseted may be due to the decrease in HIV prevalence with age. The current study also reported an 11-14% higher risk of multimorbidity with each 1kg/m^2 increase in BMI in both men and women. Overweight and obesity are well known risk factors for non-communicable diseases such as diabetes, hypertension and dyslipidaemia, particularly a low HDL-C, and obesity is a targeted risk factor by the World Health Organisation in their Global Action Plan for the prevention and control of NCDs (45). Although age and BMI were associated with multimorbidity prevalence in both men and women, lifestyle correlates displayed sex differences. In the women being a former consumer of alcohol was associated with nearly twice the risk of having multimorbidity, while men who were current, non-problematic consumers of alcohol were at lower risk of multimorbidity. Data from the same SSA cohort has reported that current alcohol consumption is lower in women compared to men at all AWI-Gen sites, with the characteristics of alcohol consumption such as type and frequency of consumption being significantly different between the sexes (23). This may explain the different associations with multimorbidity risk in the current study, and together with the finding that time spent in moderate-vigorous intensity physical activity was inversely associated with multimorbidity risk in men only, suggests that further intervention studies to reduce multimorbidity risk may need to be stratified by sex. The influence of socioeconomic patterns on disease risk is well recognised, and may differ between countries at different stages of the epidemiological transition (27). Employment was associated with a reduced risk of multimorbidity in this study and supports the inverse association between socio-economic status and disease risk reported by others in LMICs (46). We have shown that when compared to men who have never married or been in a union, men who are and men who have previously been in a union (divorced, separated, partner deceased) were at a significantly higher risk of multimorbidity. In their study of adults living in four SSA countries Ajayi et al., (47) reported similar associations with BMI but in only two of the sites, rural and peri-urban Uganda, with no associations in adults from Tanzania and South Africa. We have previously shown in the AWI-Gen cohort that married men had a higher BMI compared to their unmarried counterparts, which consisted of those who had never been married or were no longer married (48), while results from the PURE study have shown that people who have experienced marital loss are at higher cardiovascular disease risk (49).

For peer review only

Marital status may represent social position, socio-economic status as well as cultural beliefs around body size and marriage, and these results highlight the complexity of the association with disease risk.

It is acknowledged that these data are cross-sectional and single screen testing without follow up may result in the overestimation of diseases such as chronic kidney disease. A further limitation of the study is that data were only available for four of the seven conditions for the Soweto women, however description of the disease profile of this sub-sample still makes a contribution to the limited literature from LMICs. Further, only prevalence and correlates of multimorbidity could be identified, however the sample size is large and represents four SSA countries to help understand differences and similarities between populations at different stages of the epidemiological transition. These prevalence figures make an important contribution to the increasing knowledge around multimorbidity and may be particularly useful in developing indices that can be used as part of machine learning approaches in predicting multimorbidity in the future.

In conclusion, this study has shown that nearly half of the population-based sample of women and more than a third of the men between the ages of 40 and 60 years from South, East and West Africa, are living with two or more chronic diseases. Common disease clusters have been identified and future efforts should focus on managing multiple commonly occurring diseases rather than single diseases. Although the prevalence of multimorbidity is higher in women living in South Africa, it is expected that as the obesity epidemic continues to increase that the prevalence will increase in men as well as East and West Africa. Identifying correlates of multimorbidity is critical to providing focussed and effective interventions.

Contributorship statement

LM, SN and MR conceptualized the manuscript. GA, GA, PRB, SC, NC, FG, SM, EN, HS, SN, MR contributed to the design of the AWI-Gen study and/or coordinated data collection and data preparation. RM did the analyses and prepared all the tables and figures. LM wrote the first manuscript draft and RM, SN and MR offered further guidance on the analyses, interpretation and writing. All authors (LM, RM, GA, GA, PRB, SC, NC, JF, FG, CK, EM, SM, EN, FR, HS, FT, AW, SN, MR) reviewed the manuscript and approved the manuscript before submission.

Competing interests

None declared.

For peer review only

Funding

The AWI-Gen Collaborative Centre is funded by the National Human Genome Research Institute (NHGRI), the National Institute of Environmental Health Sciences (NIEHS), the Office of AIDS research (OAR) and the National Institute of Diabetes and Digestive and Kidney Diseases (NIDDK), of the National Institutes of Health (NIH) under award number U54HG006938, as part of the H3Africa Consortium, and by the Department of Science and Innovation, South Africa, award number DST/CON 0056/2014. MR is a South African Research Chair in Genomics and Bioinformatics of African populations hosted by the University of the Witwatersrand, funded by the Department of Science and Technology and administered by National Research Foundation of South Africa (NRF). The Birth to Twenty Cohort (Soweto, South Africa) is supported by University of the Witwatersrand, the South African Medical Research Council, and The Wellcome Trust, UK. SAN is supported by the DST/NRF Centre of Excellence in Human Development at the University of the Witwatersrand, Johannesburg. ANW is supported by the Fogarty International Centre, National Institutes of Health [grant number K43TW010698].

This paper describes the views of the authors and does not necessarily represent the official views of the National Institutes of Health or the National Research Foundation of South Africa who funded this research.

Data sharing statement

The data have been submitted to the European Genome-Phenome Archive (EGA), accession number EGA00001002482. The Human Heredity and Health in Africa (H3Africa) Data and Biospecimen Access Committee (DBAC) will review requests for the AWI-Gen phenotype dataset. Related documents including study protocol and statistical analysis plan will be available upon request from the corresponding author.

Acknowledgements

This study would not have been possible without the generosity of the participants who spent many hours responding to questionnaires, being measured and having samples taken. We wish to acknowledge the contributions of our field workers, phlebotomists, laboratory scientists, administrators, data personnel and other investigators who contributed to the data and sample collections, processing, storage and shipping.

For peer review only

Investigators responsible for the conception and design of the AWI-Gen study include the following Michèle Ramsay (PI, Wits), Osman Sankoh (co-PI, INDEPTH), Stephen Tollman and Kathleen Kahn (Agincourt PI), Marianne Alberts (Dikgale (renamed DIMAMO) PI (deceased)), Catherine Kyobutungi (Nairobi PI), Halidou Tinto (Nanoro PI), Abraham Oduro (Navrongo PI), Shane Norris (Soweto PI), and Scott Hazelhurst, Nigel Crowther, Himla Soodyall and Zane Lombard (Wits). We would like to acknowledge each of the following investigators for the significant contributions to this research, mentioned according to affiliation are: Wits AWI-Gen Collaborative Centre - Stuart Ali, Ananyo Choudhury, Scott Hazelhurst, Freedom Mukomana, Cassandra Soo; Soweto (DPHRU): Nomses Baloyi, Yusuf Guman.

Ethics and consent

All participants provided written informed consent before any study procedures were conducted. The Human Research Ethics Committee (Medical) of the University of the Witwatersrand approved the protocol (certificate numbers M121029 and M170880). Each study centre obtained local ethics approval as detailed in Ali et al. 2018 (14).

For peer review only

References

1. Gouda HN, Charlson F, Sorsdahl K, Ahmadzada S, Ferrari AJ, Erskine H, et al. Burden of non-communicable diseases in sub-Saharan Africa,1990–2017: results from the Global Burden of Disease Study 2017. Lancet Glob Health. 2019;7:e1375–87.
2. Tran PB, Kazibwe J, Nikolaidis GF, Linnosmaa I, Rijken M, van Olmen J. Costs of multimorbidity: a systematic review and meta-analyses. BMC Med. 2022;20:234.
3. Afshar S, Roderick PJ, Kowal P, Dimitrov BD, Hill AG. Multimorbidity and the inequalities of global ageing: A cross-sectional study of 28 countries using the World Health Surveys. BMC Public Health. 2015;15:776.
4. Mendenhall E. Syndemics: a new path for global health research. Vol. 389, The Lancet. 2017;889–91.
5. Abebe F, Schneider M, Asrat B, Ambaw F. Multimorbidity of chronic non-communicable diseases in low- and middle-income countries: A scoping review. J Comorb. 2020;10:2235042X2096191.
6. Lalkhen H, Mash R. Multimorbidity in non-communicable diseases in South African primary healthcare. South African Medical Journal. 2015;105:134–8.
7. Chidumwa G, Maposa I, Corso B, Minicuci N, Kowal P, Micklesfield LK, et al. Identifying co-occurrence and clustering of chronic diseases using latent class analysis: Cross-sectional findings from SAGE South Africa Wave 2. BMJ Open. 2021;11:e041604.
8. Nimako BA, Baiden F, Sackey SO, Binka F. Multimorbidity of chronic diseases among adult patients presenting to an inner-city clinic in Ghana. Global Health. 2013;9:61.
9. Hien H, Berthé A, Drabo MK, Meda N, Konaté B, Tou F, et al. Prevalence and patterns of multimorbidity among the elderly in Burkina Faso: Cross-sectional study. Tropical Medicine and International Health. 2014;19:1328–33.
10. Mohamed SF, Haregu TN, Uthman OA, Khayeka-Wandabwa C, Muthuri SK, Asiki G, et al. Multimorbidity from chronic conditions among adults in urban slums: The Awi-Gen Nairobi site study findings. Glob Heart. 2021;16:6.
11. Garin N, Koyanagi A, Chatterji S, Tyrovolas S, Olaya B, Leonardi M, et al. Global Multimorbidity Patterns: A Cross-Sectional, Population-Based, Multi-Country Study. Journals of Gerontology - Series A Biological Sciences and Medical Sciences. 2016;71:205–14.
12. Alaba O, Chola L. The social determinants of multimorbidity in South Africa. Int J Equity Health. 2013;12:63.

For peer review only

13. Ramsay M, Crowther N, Tambo E, Agongo G, Baloyi V, Dikotope S, et al. H3Africa AWI-Gen Collaborative Centre: A resource to study the interplay between genomic and environmental risk factors for cardiometabolic diseases in four sub-Saharan African countries. Glob Health Epidemiol Genom. 2016;1e20.

14. Ali SA, Soo C, Agongo G, Alberts M, Amenga-Etego L, Boua RP, et al. Genomic and environmental risk factors for cardiometabolic diseases in Africa: methods used for Phase 1 of the AWI-Gen population cross-sectional study. Glob Health Action. 2018;11(sup2):1507133.15.

15. Ewing JA. Detecting alcoholism. The CAGE questionnaire. JAMA. 1984;252(14):1905–7.

16. Bull FC, Maslin TS, Armstrong T. Global Physical Activity Questionnaire (GPAQ): Nine Country Reliability and Validity Study. Journal of Physical Activity and Health. 2009;6:790-804.

17. Chobanian A v., Bakris GL, Black HR, Cushman WC, Green LA, Izzo JL, et al. Seventh Report of the Joint National Committee on Prevention, Detection, Evaluation, and Treatment of High Blood Pressure. Hypertension. 2003;42:1206–52.

18. Classification and Diagnosis of Diabetes: *Standards of Medical Care in Diabetes—2021*. Diabetes Care. 2021 Jan 1;44(Supplement_1):S15–33.

19. Klug EQ, Raal FJ, Marais AD, Smuts CM, Schamroth C, Jankelow D, et al. South African dyslipidaemia guideline consensus statement: 2018 update A joint statement from the South African Heart Association (SA Heart) and the Lipid and Atherosclerosis Society of Southern Africa (LASSA). South African Medical Journal. 2018;108:975–1000.

20. Friedewald WT, Levy RI, Fredrickson DS. Estimation of the concentration of Low-Density Lipoprotein Cholesterol in plasma, without use of the preparative ultracentrifuge. Clinical Chemistry 1972;18:499-502.

21. Stevens PE, Levin A. Evaluation and Management of Chronic Kidney Disease: Synopsis of the Kidney Disease: Improving Global Outcomes 2012 Clinical Practice Guideline. Ann Intern Med. 2013;158:825–30.

22. Conway JR, Lex A, Gehlenborg N. UpSetR: An R package for the visualization of intersecting sets and their properties. Bioinformatics. 2017;33(18):2938–40.

23. Boua PR, Soo CC, Debpuur C, Maposa I, Nkoana S, Mohamed SF, et al. Prevalence and socio-demographic correlates of tobacco and alcohol use in four sub-Saharan African countries: a cross-sectional study of middle-aged adults. BMC Public Health. 2021;21:1126.

For peer review only

24. Schäfer I, Kaduszkiewicz H, Nguyen TS, van den Bussche H, Scherer M, Schön G. Multimorbidity patterns and 5-year overall mortality: Results from a claims data–based observational study. J Comorb. 2018;8(1):2235042X1881658.
25. Willadsen TG, Siersma V, Nicolaisdóttir DR, Køster-Rasmussen R, Jarbøl DE, Reventlow S, et al. Multimorbidity and mortality: A 15-year longitudinal registry-based nationwide Danish population study. J Comorb. 2018;8; 2235042X18804063.
26. Roomaney RA, van Wyk B, Turawa EB, Pillay-van Wyk V. Multimorbidity in South Africa: a systematic review of prevalence studies. BMJ Open. 2021;11(10):e048676.
27. Stringhini S, Forrester TE, Plange-Rhule J, Lambert E v., Viswanathan B, Riesen W, et al. The social patterning of risk factors for noncommunicable diseases in five countries: Evidence from the modeling the epidemiologic transition study (METS). BMC Public Health. 2016;16(1).
28. Ramsay M, Crowther NJ, Agongo G, Ali SA, Asiki G, Boua RP, et al. Regional and sex-specific variation in BMI distribution in four sub-Saharan African countries: The H3Africa AWI-Gen study. Glob Health Action. 2018 Nov 16;11(sup2): 1556561.
29. Chang AY, Gómez-Olivé FX, Payne C, Rohr JK, Manne-Goehler J, Wade AN, et al. Chronic multimorbidity among older adults in rural South Africa. BMJ Glob Health. 2019;4:e001386.
30. Wade AN, Payne CF, Berkman L, Chang A, Gómez-Olivé FX, Kabudula C, et al. Multimorbidity and mortality in an older, rural black South African population cohort with high prevalence of HIV findings from the HAALSI Study. BMJ Open. 2021:;11(9).
31. Kabudula CW, Houle B, Collinson MA, Kahn K, Gómez-Olivé FX, Clark SJ, et al. Progression of the epidemiological transition in a rural South African setting: findings from population surveillance in Agincourt, 1993-2013. BMC Public Health. 2017;17(1).
32. Hoffman RM, Chibwana F, Kahn D, Banda BA, Phiri L, Chimombo M, et al. High Rates of Uncontrolled Blood Pressure in Malawian Adults Living with HIV and Hypertension. Glob Heart. 2021;16:81.
33. Stokes A, Berry KM, Mchiza Z, Parker W ah, Labadarios D, Chola L, et al. Prevalence and unmet need for diabetes care across the care continuum in a national sample of South African adults: Evidence from the SANHANES-1, 2011-2012. PLoS One. 2017;12:e0184264.
34. Gómez-Olivé FX, Ali SA, Made F, Kyobutungi C, Nonterah E, Micklesfield L, et al. Regional and Sex Differences in the Prevalence and Awareness of Hypertension: An

For peer review only

H3Africa AWI-Gen Study Across 6 Sites in Sub-Saharan Africa. Glob Heart. 2017;12(2):81–90.

35. Violan C, Foguet-Boreu Q, Flores-Mateo G, Salisbury C, Blom J, Freitag M, et al. Prevalence, Determinants and Patterns of Multimorbidity in Primary Care: A Systematic Review of Observational Studies. PLoS One. 2014;9(7):e102149.

36. Chori B, Danladi B, Nwakile P, Okoye I, Abdullahi U, Zawaya K, et al. Prevalence, patterns and predictors of dyslipidaemia in Nigeria: a report from the REMAH study. Cardiovasc J Afr. 2022;33(2):14–21.

37. Fiseha T, Alemu W, Dereje H, Tamir Z, Gebreweld A. Prevalence of dyslipidaemia among HIV-infected patients receiving combination antiretroviral therapy in North Shewa, Ethiopia. PLoS One. 2021;16:e0250328.

38. Masilela C, Adeniyi OV, Benjeddou M. Prevalence, patterns and determinants of dyslipidaemia among South African adults with comorbidities. Sci Rep. 2022;12:337.

39. Reiger S, Jardim TV, Abrahams-Gessel S, Crowther NJ, Wade A, Gomez-Olive FX, et al. Awareness, treatment, and control of dyslipidemia in rural South Africa: The HAALSI (Health and Aging in Africa: A Longitudinal Study of an INDEPTH Community in South Africa) study. PLoS One. 2017;12: e0187347.

40. Klop B, Elte JWF, Cabezas MC. Dyslipidemia in Obesity: Mechanisms and Potential Targets. Nutrients. 2013;5:1218–40.

41. Ference BA, Ginsberg HN, Graham I, Ray KK, Packard CJ, Bruckert E, et al. Low-density lipoproteins cause atherosclerotic cardiovascular disease. 1. Evidence from genetic, epidemiologic, and clinical studies. A consensus statement from the European Atherosclerosis Society Consensus Panel. Eur Heart J. 2017;38:2459–72.

42. Gaziano TA, Abrahams-Gessel S, Gomez-Olive FX, Wade A, Crowther NJ, Alam S, et al. Cardiometabolic risk in a population of older adults with multiple co-morbidities in rural South Africa: the HAALSI (Health and Aging in Africa: longitudinal studies of INDEPTH communities) study. BMC Public Health. 2017;17:1–10.

43. Ajayi SO, Ekrikpo UE, Ekanem AM, Raji YR, Ogah OS, Ojji DB, et al. Prevalence of Chronic Kidney Disease as a Marker of Hypertension Target Organ Damage in Africa: A Systematic Review and Meta-Analysis. International Journal of Hypertension.2021: 7243523.

44. Bikbov B, Purcell CA, Levey AS, Smith M, Abdoli A, Abebe M, et al. Global, regional, and national burden of chronic kidney disease, 1990–2017: a systematic analysis for the Global Burden of Disease Study 2017. The Lancet. 2020;395:709–33.

For peer review only

45. World Health Organisation. WHO Global NCD Action Plan 2013-2020. 2013.

46. Hosseinpoor AR, Bergen N, Kunst A, Harper S, Guthold R, Rekve D, et al. Socioeconomic inequalities in risk factors for non communicable diseases in low-income and middle-income countries: results from the World Health Survey. BMC Public Health. 2012;12:912.

47. Ajayi IOO, Adebamowo C, Adami HO, Dalal S, Diamond MB, Bajunirwe F, et al. Urban-rural and geographic differences in overweight and obesity in four sub-Saharan African adult populations: a multi-country cross-sectional study. BMC Public Health. 2016;16:1–13.

48. Micklesfield LK, Kagura J, Munthali R, Crowther NJ, Jaff N, Gradidge P, et al. Demographic, socio-economic and behavioural correlates of BMI in middle-aged black men and women from urban Johannesburg, South Africa. Glob Health Action. 2018;11:1448250.

49. Egbujie BA, Igumbor EU, Puoane T. A cross-sectional study of socioeconomic status and cardiovascular disease risk among participants in the prospective Urban Rural Epidemiological (PURE) study. South African Medical Journal. 2016;106:900–6.

For peer review only

Figures

Figure 1: Prevalence of 0, 1, 2, 3 and 4 or more chronic conditions (including HIV infection, cardiovascular disease, kidney disease, asthma, diabetes, dyslipidemia and hypertension) in men and women from South, East and West Africa, and the full AWI-Gen cohort

Figure 2: Multimorbidity clustering for the total AWI-Gen cohort in a) women and b) men

Figure 3: Multimorbidity clustering in a) South African women; b) South African men; c) East African women; d) East African men; e) West African women; f) West African men

Supplementary Figure 1: Multimorbidity clustering in Agincourt men from the AWI-Gen study

Supplementary Figure 2: Multimorbidity clustering in Agincourt women from the AWI-Gen study

Supplementary Figure 3: Multimorbidity clustering in Dikgale men from the AWI-Gen study

Supplementary Figure 4: Multimorbidity clustering in Dikgale women from the AWI-Gen study

Supplementary Figure 5: Multimorbidity clustering in Soweto men from the AWI-Gen study

Supplementary Figure 6: Multimorbidity clustering in Soweto women from the AWI-Gen study

Supplementary Figure 7: Multimorbidity clustering in Nairobi men from the AWI-Gen study

Supplementary Figure 8: Multimorbidity clustering in Nairobi women from the AWI-Gen study

Supplementary Figure 9: Multimorbidity clustering in Nanoro men from the AWI-Gen study

Supplementary Figure 10: Multimorbidity clustering in Nanoro women from the AWI-Gen study

Supplementary Figure 11: Multimorbidity clustering in Navrongo men from the AWI-Gen study

Supplementary Figure 12: Multimorbidity clustering in Navrongo women from the AWI-Gen study

Tables

Table 1: Multinomial logistic regression to determine factors associated with either one condition or at least two conditions, with none as the reference group, in AWI-Gen women

Table 2: Multinomial logistic regression to determine factors associated with either one condition or at least two conditions, with none as the reference group, in AWI-Gen men

Supplementary Table 1: Descriptive characteristics of socio-demographic, socio-economic and lifestyle factors of men and women from the AWI-Gen Agincourt, Dikgale and Soweto sites

Supplementary Table 2: Descriptive characteristics of socio-demographic, socio-economic and lifestyle factors of men and women from the AWI-Gen Nairobi, Nanoro and Navrongo sites

Supplementary Table 3: Prevalence of the seven chronic diseases and multimorbidity of men and women from the AWI-Gen Agincourt, Dikgale and Soweto sites

For peer review only

Supplementary Table 4: Prevalence of the seven chronic diseases and multimorbidity of men and women from the AWI-Gen Nairobi, Nanoro and Navrongo sites

For peer review only

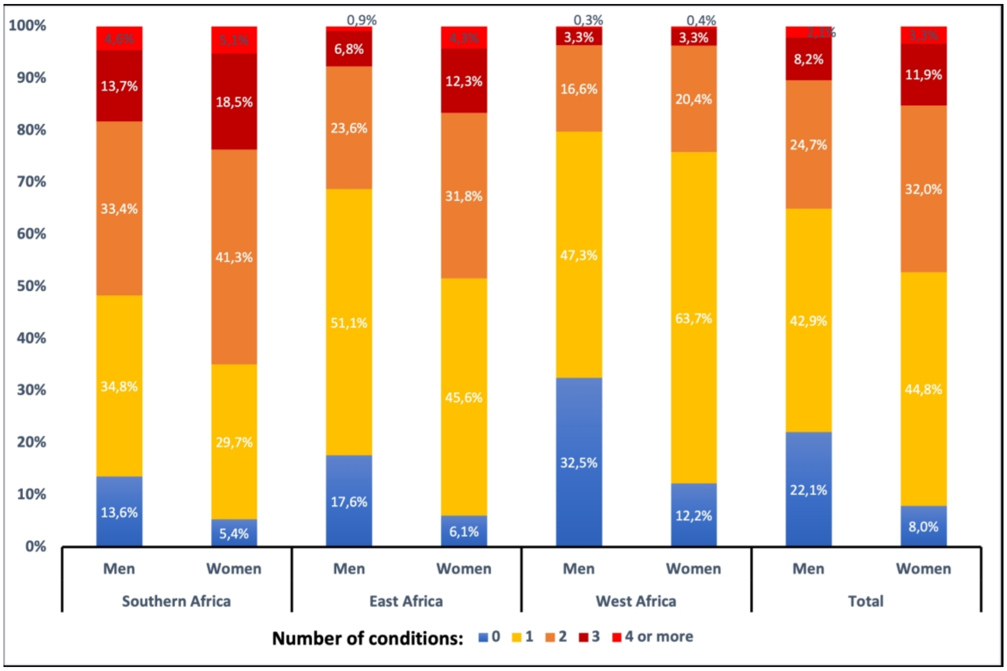

Prevalence of 0, 1, 2, 3 and 4 or more chronic conditions (including HIV infection, cardiovascular disease, kidney disease, asthma, diabetes, dyslipidemia and hypertension) in men and women from South, East and West Africa, and the full AWI-Gen cohort

479x317mm (130 x 130 DPI)

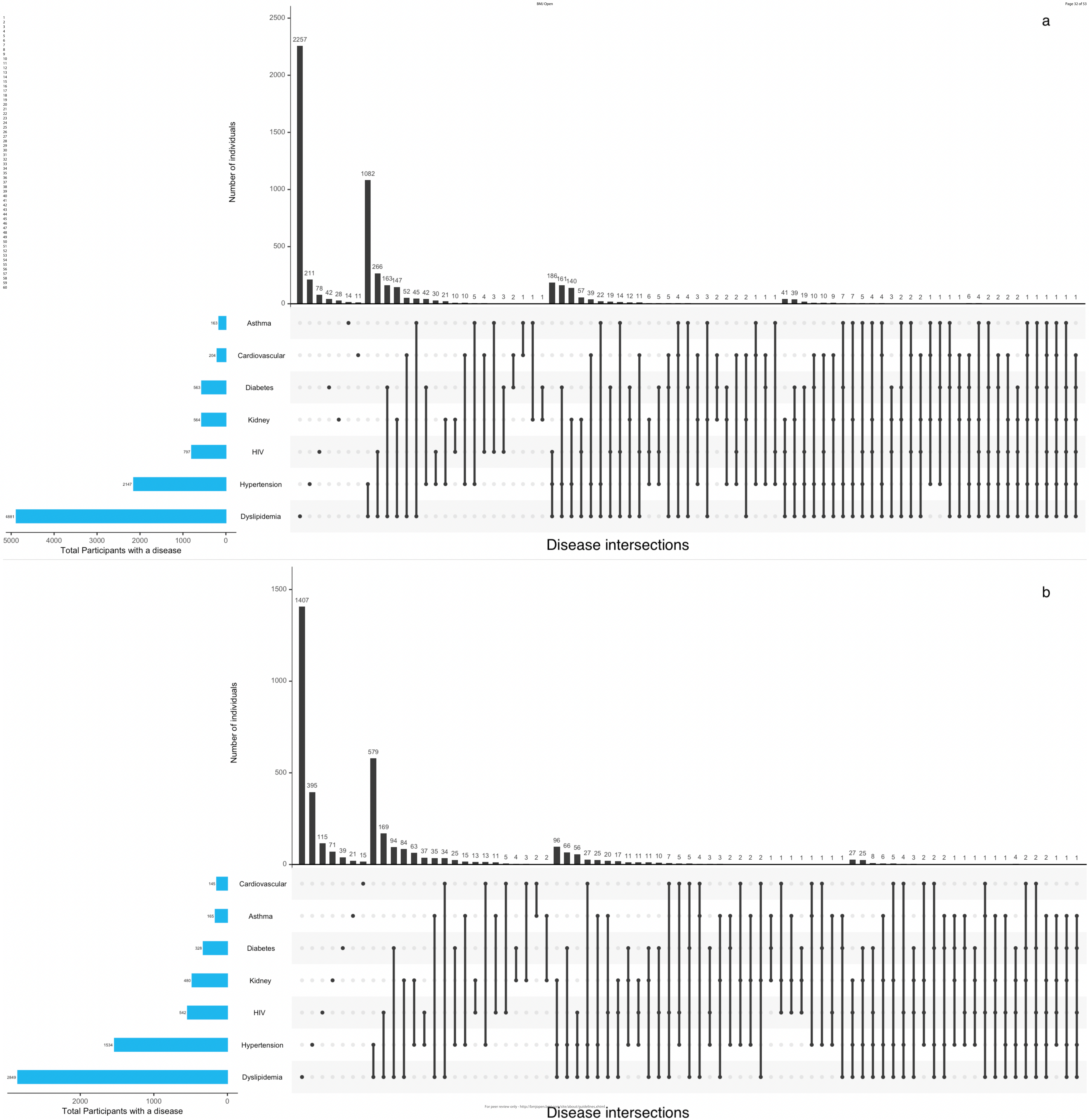
a
Number of individuals
2500
2000
1500
1000
500
0
2257 211 78 42 28 14 11 1082 266 163 147 52 45 42 30 21 10 10 5 4 3 3 2 1 1 1 186 161 140 57 39 22 19 14 12 11 6 5 5 4 4 3 3 2 2 2 2 1 1 1 41 39 19 10 10 9 7 7 5 4 4 3 2 2 2 1 1 1 1 6 4 2 2 2 2 1 1 1 1 1 1
163 Asthma
204 Cardiovascular
563 Diabetes
564 Kidney
797 HIV
2147 Hypertension
4881 Dyslipidemia
5000 4000 3000 2000 1000 0
Total Participants with a disease
Disease intersections
b
Number of individuals
1500
1000
500
0
1407 395 115 71 39 21 15 579 169 94 84 63 37 35 34 25 15 13 13 11 5 4 3 2 2 96 66 56 27 25 20 17 11 11 11 10 7 5 5 4 3 3 2 2 2 2 1 1 1 1 1 1 1 1 27 25 8 6 5 4 3 2 2 2 1 1 1 1 1 1 1 4 2 2 2 1 1 1
145 Cardiovascular
165 Asthma
328 Diabetes
480 Kidney
542 HIV
1534 Hypertension
2849 Dyslipidemia
2000 1000 0
Total Participants with a disease
Disease intersections

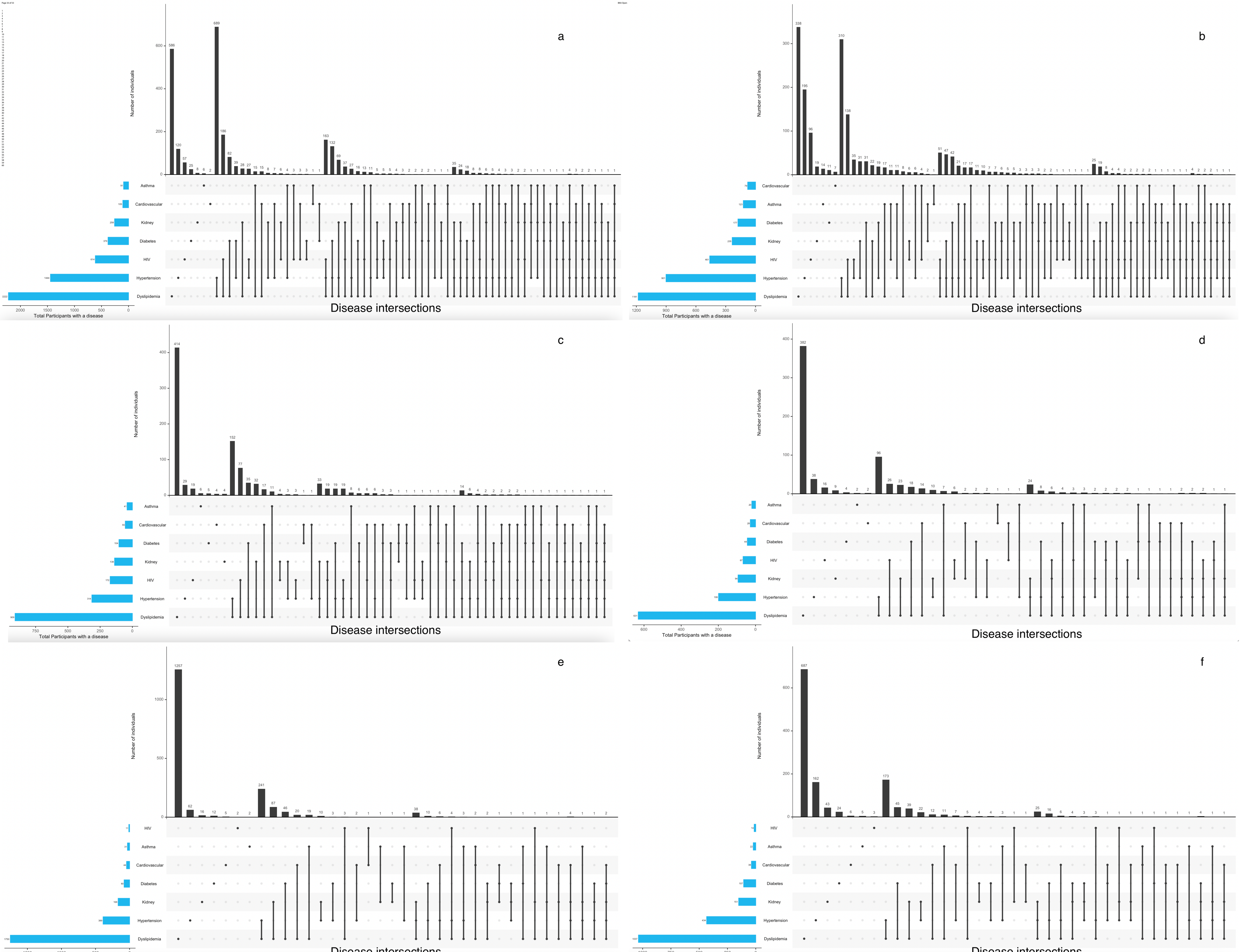

For peer review only - http://bmjopen.bmj.com/site/about/guidelines.xhtml

Supplementary Table 1

	Agincourt, SA			Dikgale, SA			Soweto, SA		
	Men (n=573)	Women (n=892)	Total (n=1465)	Men (n=356)	Women (n=812)	Total (n=1168)	Men (n=1025)	Women (n=1002)	Total (n=2027)
**Age (yrs)**	51 (45-56)	51 (46-56)	51 (46-56)	50 (45-55)	51 (46-56)	51 (45-55)	49 (44-55)	49 (44-54)	49 (44-54)
**Highest level of education attained**									
No formal	123 (21.5)	280 (31.4)	403 (27.5)*	22 (6.2)	74 (9.1)	96 (8.2)	8 (0.8)	2 (0.3)	10 (0.6)*
Primary	235 (41.1)	340 (38.1)	575 (39.3)	113 (31.7)	273 (33.7)	386 (33.1)	117 (11.4)	636 (80.9)	753 (41.6)
Secondary	175 (30.6)	223 (25.0)	398 (27.2)	204 (57.3)	440 (54.2)	644 (55.2)	749 (73.0)	147 (18.7)	895 (49.4)
Tertiary	39 (6.8)	49 (5.5)	88 (6.0)	17 (4.8)	24 (3.0)	41 (3.5)	152 (14.8)	1 (0.1)	153 (8.4)
**Employment**									
Unemployed	326 (62.3)	558 (64.8)	884 (63.9)	193 (54.7)	532 (65.6)	725 (62.3)*	355 (34.6)	455 (45.4)	810 (40.0)*
Employed	197 (37.7)	303 (35.2)	500 (36.1)	160 (45.3)	279 (34.4)	439 (37.7)	670 (65.4)	547 (54.6)	1217 (60.0)
**Partnership status**									
Never married or co-habited	75 (13.1)	59 (6.3)	134 (9.2)*	103 (28.9)	185 (22.8)	288 (24.7)	265 (25.9)	51 (7.7)	316 (18.7)*
Married or co-habiting	445 (77.1)	537 (60.2)	982 (67.0)	178 (50.0	427 (52.6)	605 (51.8)	570 (55.7)	266 (40.1)	836 (49.5)
Divorced, separated, partner deceased	53 (9.3)	296 (33.2)	349 (23.8)	75 (21.1)	200 (24.6)	275 (23.5)	189 (18.5)	347 (52.3)	536 (31.8)
**SES site quintile**	3 (2-4)	4 (2-5)	3 (2-5)*	3 (2-4)	4 (2-5)	4 (2-5)*	4 (3-5)	2 (2-3)	3 (2-5)*
**BMI (kg/m^2)**	23 (20-27)	28.6 (24-33)	26 (22-31) *	20.6 (19-24)	30.1 (25-36)	26.9 (21-33)*	24.2 (20-28)	32.9 (29-38)	28.4 (23-34)*
**Smoking**									
Never smoker	291 (51.0)	882 (98.8)	1173(80.1)*	54 (15.2)	757 (93.3)	811 (69.6)*	311 (30.4)	893 (89.9)	1204(59.7)*
Current smoker	155 (27.1)	3 (0.3)	158 (10.8)	225 (63.4)	25 (3.1)	250 (21.4)	540 (52.8)	49 (4.9)	589 (29.2)

For peer review only

	Agincourt, SA			Dikgale, SA			Soweto, SA		
	Men (n=573)	Women (n=892)	Total (n=1465)	Men (n=356)	Women (n=812)	Total (n=1168)	Men (n=1025)	Women (n=1002)	Total (n=2027)
Previous smoker	125 (21.9)	7 (0.8)	132 (9.0)	76 (21.4)	29 (3.6)	105 (9.0)	172 (16.8)	51 (5.1)	223 (11.1)
**Alcohol status**									
Never consumed alcohol	187 (32.6)	731 (82.0)	918 (62.7)*	55 (15.5)	561 (69.3)	616 (52.9)*			
Current non-problematic consumer	218 (38.1)	52 (5.8)	270 (18.4)	77 (21.6)	45 (5.6)	122 (10.5)	726 (100.0)		726 (100.0)
Current problematic consumer	18 (3.1)	2 (0.2)	20 (1.4)	142 (39.9)	57 (7.1)	199 (17.1)			
Former consumer	150 (26.2)	107 (12.0)	257 (17.5)	82 (23.0)	146 (18.0)	228 (19.9)			
**GPAQ MVPA (mins/wk)/100**	7.2 (2-14.4)	6 (1.8-13.8)	6.3 (1.8-14.1)	11.4 (6.6-25.8)	12.6 (7.2-21)	12.1 (7.2-21.9)	6.6 (2.1-17.4)	2.1 (0.6-8.6)	1.2 (4.15-13.8)*

Continuous data presented as median (IQR) and categorical data as n(%); * = p<0.05 for difference between men and women in respective sites.

For peer review only

Supplementary Table 2

	Nairobi, Kenya			Nanoro, Burkina Faso			Navrongo, Ghana		
	Men (n=886)	Women (n=1056)	Total (n=1942)	Men (n=1045)	Women (n=1039)	Total (n=2084)	Men (n=923)	Women (n=1091)	Total (n=2014)
**Age (yrs)**	48 (44-53)	48 (44-52)	48 (44-53)	50 (44-55)	50 (45-54)	50 (45-55)	50 (46-55)	52 (47-56)	51 (46-56)*
**Highest level of education attained (%)**									
No formal	34 (3.8)	113 (10.7)	147 (7.6)*	758 (72.8)	960 (93.2)	1718 (83.0)*	570 (61.9)	843 (77.6)	1413 (70.4)*
Primary	447 (50.5)	663 (62.8)	1110 (57.2)	181 (17.4)	58 (5.6)	239 (11.5)	206 (22.4)	177 (16.3)	383 (19.1)
Secondary	383 (43.2)	276 (26.1)	659 (34.0)	86 (8.3)	10 (1.0)	96 (4.6)	118 (12.8)	57 (5.3)	175 (8.7)
Tertiary	22 (2.5)	4 (0.4)	26 (1.3)	16 (1.5)	2 (0.2)	18 (0.9)	27 (2.9)	9 (0.8)	36 (1.8)
**Employment (%)**									
Unemployed	23 (2.6)	90 (8.5)	113 (5.8)*	16 (1.5)	5 (.5)	21 (1.0)*	321 (34.9)	429 (39.4)	750 (37.4)*
Employed	860 (97.4)	966 (91.8)	1826 (94.2)	1026 (98.5)	1030 (99.5)	2056 (99.0)	599 (65.1)	659 (60.6)	1258 (62.6)
**Partnership status (%)**									
Never married or co-habited	13 (1.5)	70 (6.6)	83 (4.3)*	14 (1.3)	3 (0.3)	17* (0.8)	15 (1.6)	5 (0.5)	20* (1.0)
Married or co-habiting	808 (91.2)	486 (46.1)	1,294 (66.7)	1021 (97.9)	794 (76.7)	1815 (87.3)	787 (85.4)	694 (63.6)	1481 (73.6)
Divorced, separated, partner deceased	65 (7.3)	499 (47.3)	564 (29.1)	8 (0.8)	238 (23.0)	246 (11.8)	120 (13.0)	392 (35.9)	512 (25.4)
**SES site quintile (median)**	3 (2-5)	3 (2-4)	3 (2-4)*	3 (2-5)	3 (2-4)	3 (2-5)*	3 (2-5)	3 (2-4)	3 (2-4)*
**BMI (median)**	22.2 (20-25)	26.9 (23-32)	24.4 (21-29)*	21.1 (19-23)	19.8 (18-22)	20.4 (19-23)*	20.6 (19-22)	21.4 (20-24)	21 (19-23)*
**Smoking (%)**									
Never smoker	418 (47.2)	975 (92.3)	1393 (71.8)*	779 (74.7)	1033 (99.8)	1812 (87.2)*	332 (36.0)	1052 (96.6)	1384 (68.8)*
Current smoker	208 (23.5)	27 (2.6)	235 (12.1)	142 (13.6)	0.0 (0.0)	142 (6.8)	388 (42.0)	21 (1.9)	409 (20.3)

For peer review only

	Nairobi, Kenya			Nanoro, Burkina Faso			Navrongo, Ghana		
	Men (n=886)	Women (n=1056)	Total (n=1942)	Men (n=1045)	Women (n=1039)	Total (n=2084)	Men (n=923)	Women (n=1091)	Total (n=2014)
Previous smoker	259 (29.3)	54 (5.1)	313 (16.1)	122 (11.7)	2 (0.2)	124 (6.0)	203 (22.0)	16 (1.5)	219 (10.9)
**Alcohol status (%)**									
Never consumed alcohol	257 (29.0)	758 (71.8)	1015 (52.3)*	273 (26.2)	277 (26.7)	550 (26.5)*	71 (7.7)	231 (21.2)	302 (15.0)*
Current non-problematic consumer	174 (19.6)	32 (3.0)	206 (10.6)	544 (52.3)	520 (50.2)	1064 (51.2)	252 (27.4)	426 (39.1)	678 (33.7)
Current problematic consumer	126 (14.2)	30 (2.8)	156 (8.0)	160 (15.4)	100 (9.67	260 (12.5)	461 (50.0)	167 (15.3)	628 (31.3)
Former consumer	329(37.1)	236 (22.4)	565 (29.1)	64 (6.1)	139 (13.4)	203 (9.8)	137 (14.9)	265 (24.3)	402 (20.0)
**GPAQ MVPA (mins/wk)/100**	12.6 (5.4-31.8)	8.4 (3.9-21.6)	10.5 (4.2-26)*	21.6 (1.2-30.6)	29.4 (5.7-36)	25.2 (3.6-33.6)*	24 (9-30.6)	16.2 (3-26.1)	19.8 (4.5-28.8)*

Continuous data presented as median (IQR) and categorical data as n(%); * = p<0.05 for difference between men and women in respective sites.

For peer review only

Supplementary Table 3

	Agincourt: SA			Dikgale: SA			Soweto, SA		
	Men (n=573)	Women (n=892)	Total (n=1465)	Men (n=356)	Women (n=812)	Total (n=1168)	Men (n=1025)	Women (n=1002)	Total (n=2027)
HIV	190 (33.2)	315 (35.3)	505 (34.5)	73 (19.0)	178 (22.2)	251 (21.9)	198 (20.5)	121 (20.5)	319 (21.1)
Asthma	65 (12.8)	58 (8.0)	123 (10.0)	22 (6.6)	33 (4.2)	55 (4.9)	36 (3.6)		36 (3.6)
Diabetes	55 (9.6)	84 (9.4)	139 (9.5)	33 (9.3)	105 (12.9)	138 (11.8)	89 (8.7)	190 (19.0)	279 (13.8)
Chronic kidney disease	76 (13.9)	145 (17.4)	221 (16.0)	30 (9.4)	113 (15.5)	143 (13.6)	129 (13.4)	-	129 (13.4)
Dyslipidaemia	334 (58.3)	740 (83.0)	1074 (73.3)	208 (58.4)	710 (87.4)	918 (78.6)	639 (62.3)	772 (77.1)	1,411 (69.6)
Hypertension	248 (43.3)	513 (57.5)	761 (52.0)	114 (43.2)	391 (48.1)	505 (43.2)	539 (52.6)	540 (53.9)	1079 (53.2)
Cardiovascular disease	22 (3.8)	46 (5.2)	68 (4.6)	21 (5.9)	59 (7.3)	80 (6.9)	36 (3.5)		36 (3.5)
Number of conditions									
0	62 (10.8)	38 (4.3)	100 (6.8) *	72 (20.2)	36 (4.7)	108 (9.3) *	131 (12.8)	71 (7.1)	202 (10.0) *
1	196 (34.2)	193 (21.6)	389 (26.5)	133 (37.4)	243 (29.9)	376 (32.2)	351 (34.2)	368 (36.7)	719 (35.5)
2	192 (33.5)	354 (39.7)	546 (37.3)	102 (28.6)	323 (39.8)	425 (36.4)	358 (34.9)	440 (43.9)	798 (39.4)
3	89 (15.5)	236 (26.5)	325 (22.2)	36 (10.1)	148 (18.2)	184 (15.8)	143 (14.0)	117 (11.7)	260 (12.8)
4	27 (4.7)	63 (7.1)	90 (6.1)	9 (2.5)	55 (6.8)	64 (5.5)	40 (3.9)	6 (0.6)	46 (2.3)
5	7 (1.2)	8 (0.9)	15 (1.0)	4 (1.1)	6 (0.7)	10 (0.9)	2 (0.2)	-	2 (0.1)
6	0	0	0	0	1 (0.1)	1 (0.1)	0	-	0
Multimorbidity	315 (54.9)	661 (74.1) *	976 (66.6)	151 (42.4)	533 (65.5) *	684 (58.6)	543 (53)	563 (56.2) *	1106 (54.6)

Data presented as n(%); * = p<0.05 for difference between men and women in respective sites

For peer review only

Supplementary Table 4

	Nairobi, Kenya			Nanoro, Burkina Faso			Navrongo, Ghana		
	Men (n=886)	Women (n=1056)	Total (n=1942)	Men (n=1045)	Women (n=1039)	Total (n=2084)	Men (n=923)	Women (n=1091)	Total (n=2014)
HIV	67 (8.9)	172 (17.7)	239 (13.8)	5 (0.5)	4 (0.4)	9 (0.4)	9 (1.0)	7 (0.6)	16 (0.8)
Asthma	20 (2.3)	41 (4.0)	61 (3.2)	19 (2.1)	23 (2.2)	42 (2.2)	3 (0.3)	8 (0.7)	11 (0.5)
Diabetes	44 (5.0)	104 (9.2)	148 (7.6)	65 (6.2)	32 (3.1)	97 (4.6)	42 (4.6)	48 (4.4)	90 (4.5)
Chronic kidney disease	94 (11.5)	138 (14.4)	232 (13.1)	73 (7.4)	81 (7.9)	154 (7.7)	78 (9.0)	87 (8.5)	165 (8.7)
Dyslipidaemia	631 (71.2)	909 (86.1)	1540(79.3)	643 (61.5)	889 (85.6)	1,532 (73.5)	394 (42.7)	861 (78.9)	1,255 (62.3)
Hypertension	199 (22.5)	314 (29.7)	513 (26.4)	211 (20.2)	124 (11.9)	335 (16.1)	223 (24.2)	265 (24.3)	488 (24.2)
Cardiovascular disease	28 (3.2)	55 (5.2)	83 (4.3)	15 (1.4)	15 (1.4)	31 (1.5)	23 (2.5)	28 (2.6)	51 (2.5)
Number of conditions									
0	156 (17.6)	64 (6.1)	220 (11.3) *	295 (28.2)	113 (10.9)	408 (19.6) *	345 (37.4)	147 (13.5)	492 (24.4) *
1	453 (51.1)	481 (45.6)	934 (48.1)	518 (49.6)	716 (68.9)	1234 (59.2)	412 (44.6)	640 (58.7)	1052 (52.2)
2	209 (23.6)	336 (31.8)	545 (28.1)	188 (18.0)	183 (17.6)	371 (17.8)	139 (15.1)	252 (23.1)	391 (19.4)
3	60 (6.8)	130 (12.3)	190 (9.8)	39 (3.7)	22 (2.1)	61 (2.9)	26 (2.8)	49 (4.5)	75 (3.7)
4	8 (0.9)	38 (3.6)	16 (0.8)	5 (0.5)	4 (0.4)	9 (0.4)	1 (0.1)	2 (0.2)	3 (0.1)
5	0	4 (0.4)	4 (0.2)	0	1 (0.1)	1 (0.1)	0	1 (0.1)	1 (0.1)
6	0	3 (0.3)	3 (0.2)	0	0	0	0	0	0
Multimorbidity	277 (31.3)	511 (48.4) *	758 (39)	232 (22.2)	210 (20.2) *	442 (21.2)	166 (18)	304 (27.9) *	470 (23.3)

Data presented as n(%); * = p<0.05 for difference between men and women in respective site

For peer review only

For peer review only - http://bmjopen.bmj.com/site/about/guidelines.xhtml

For peer review only

Supplementary figure 1

a

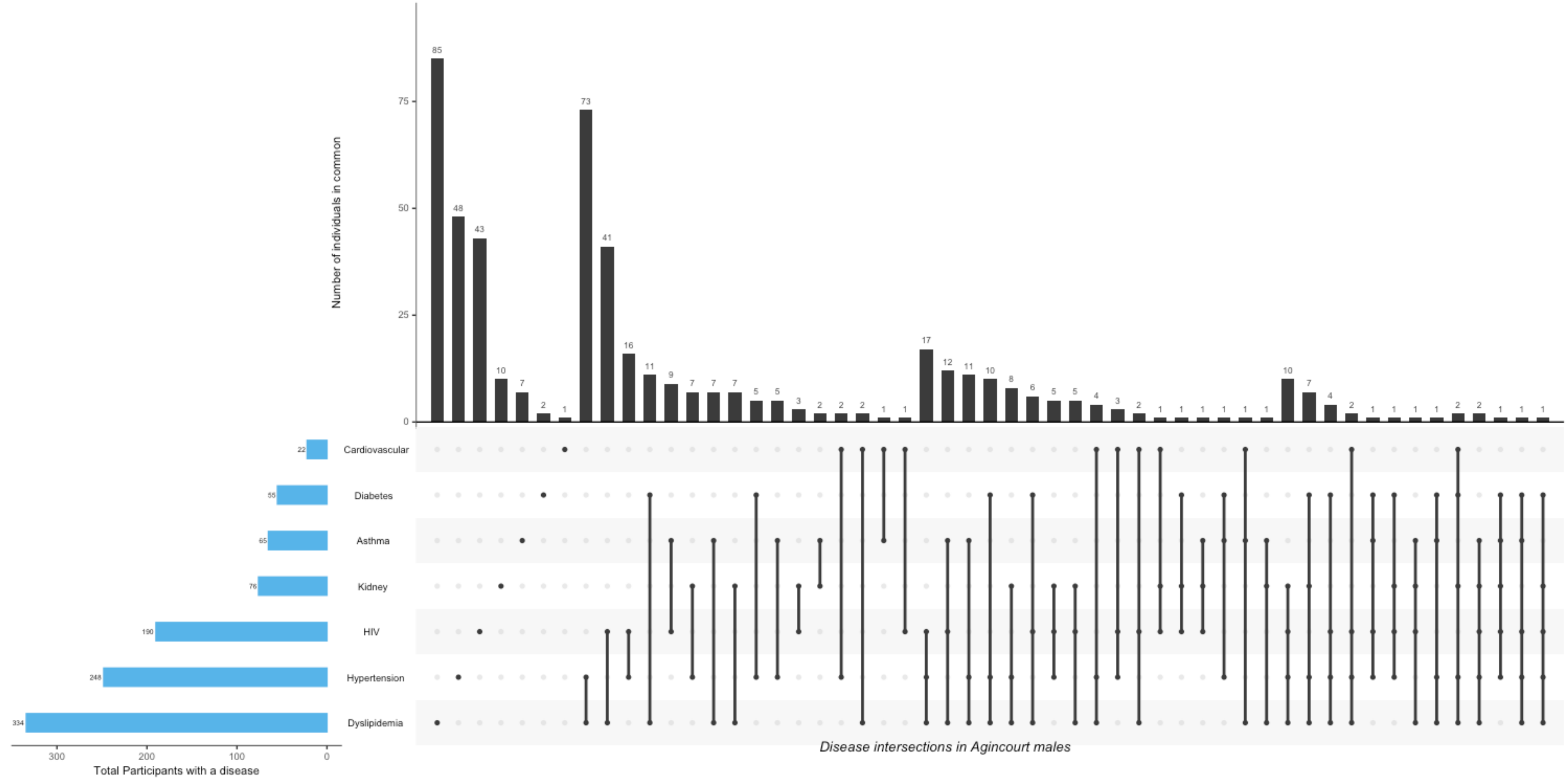

Supplementary figure 2

b

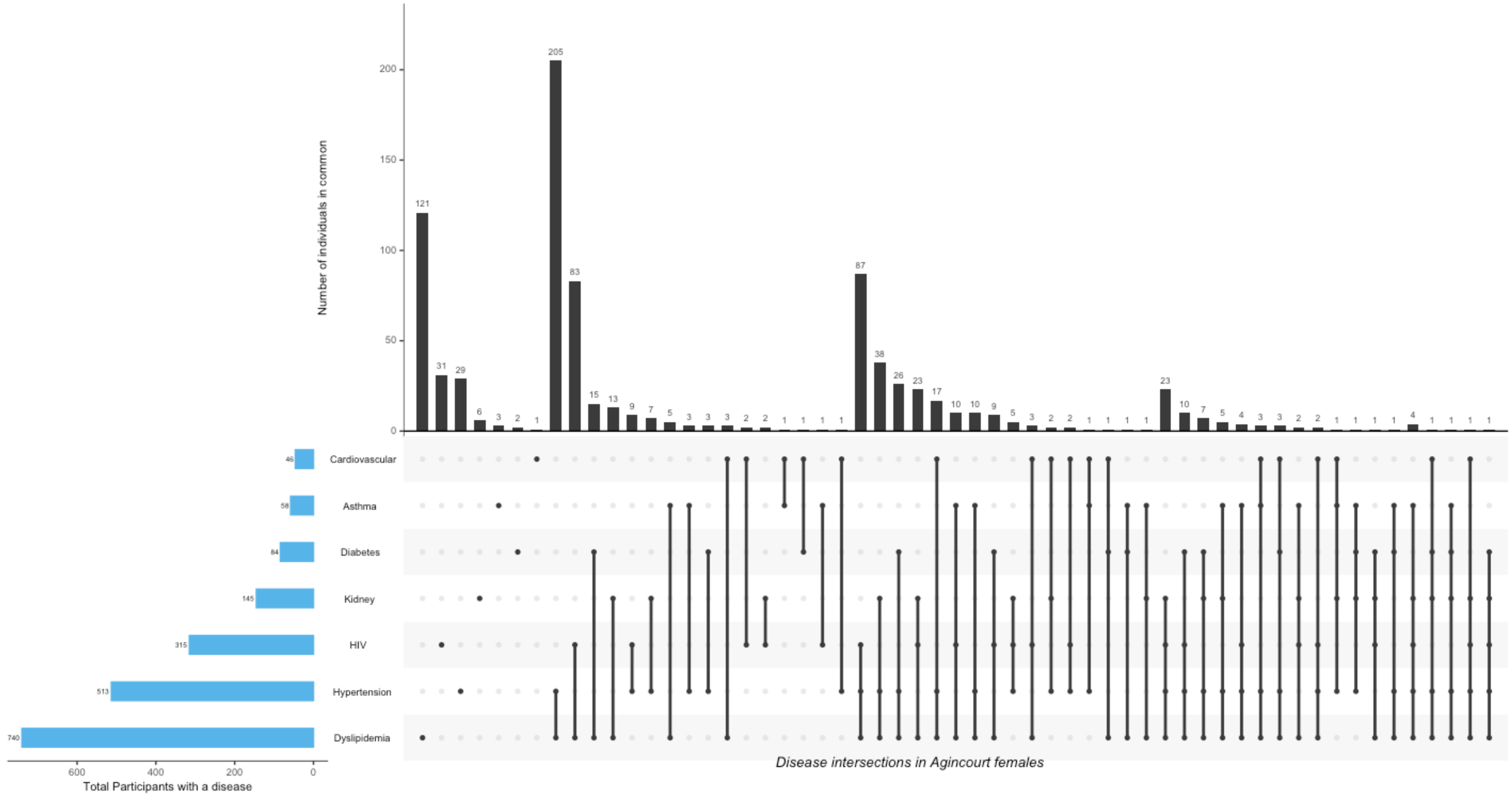

Supplementary figure 3

c

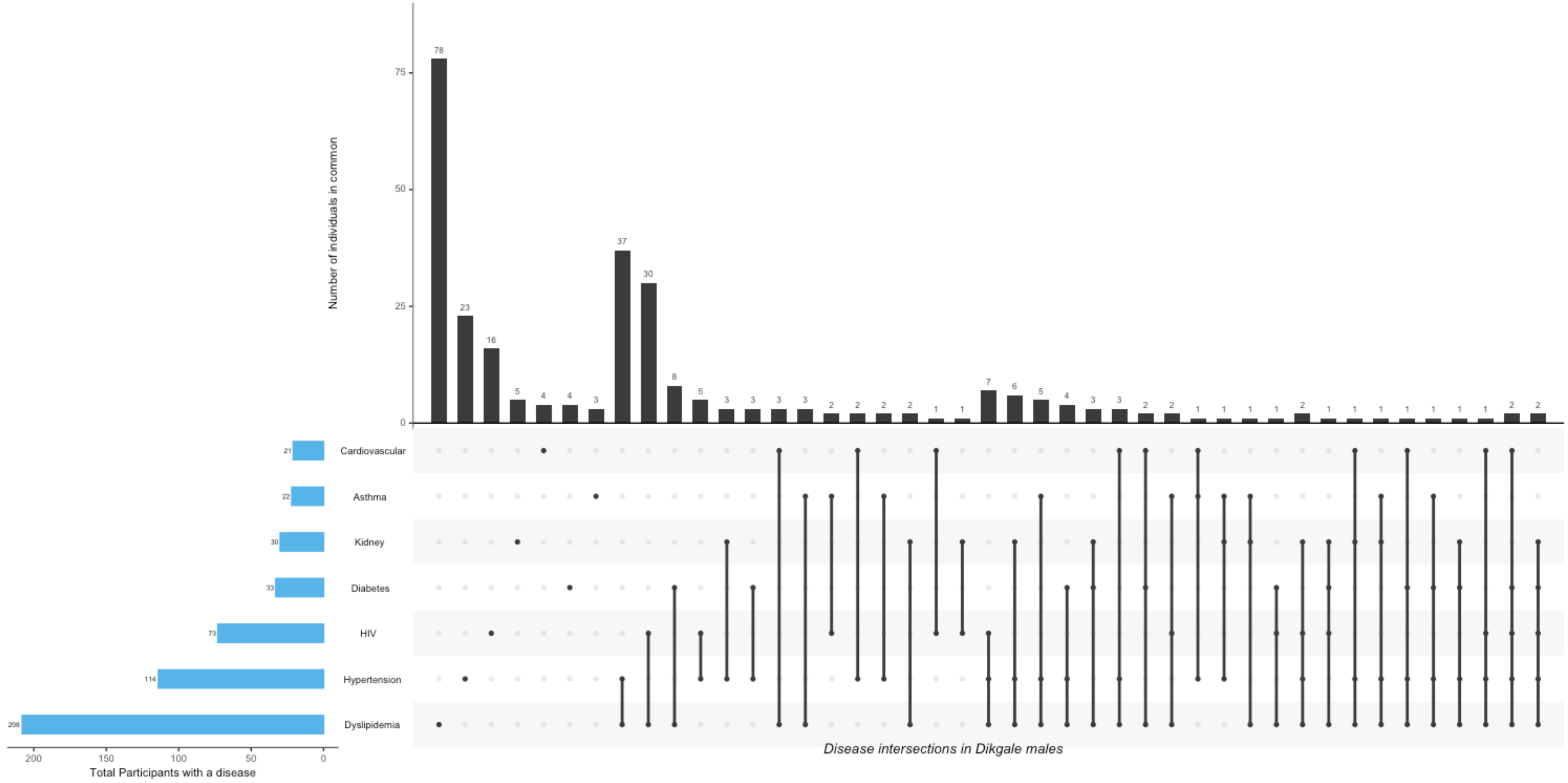

Supplementary figure 4

d

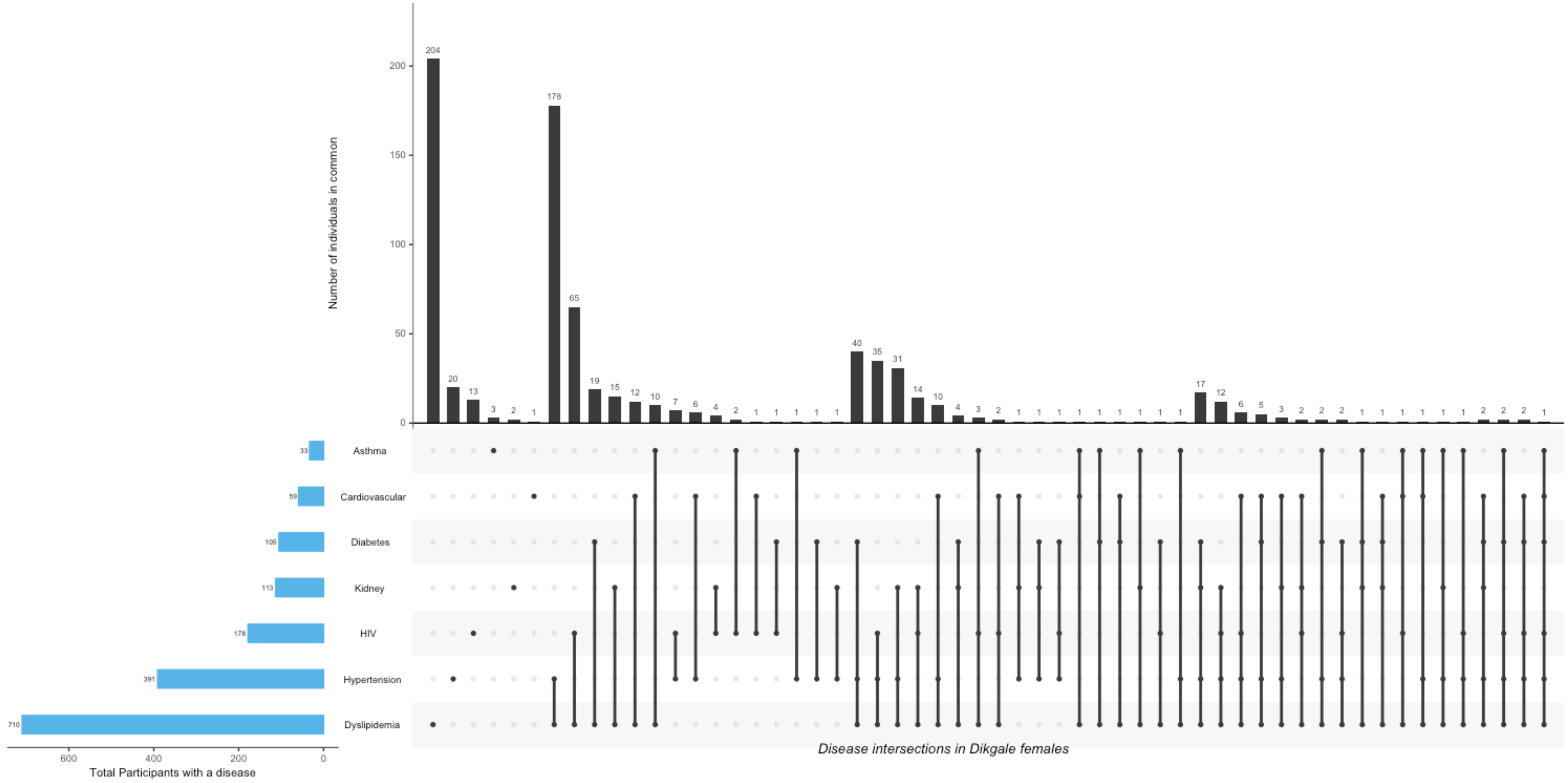

Supplementary figure 5

e

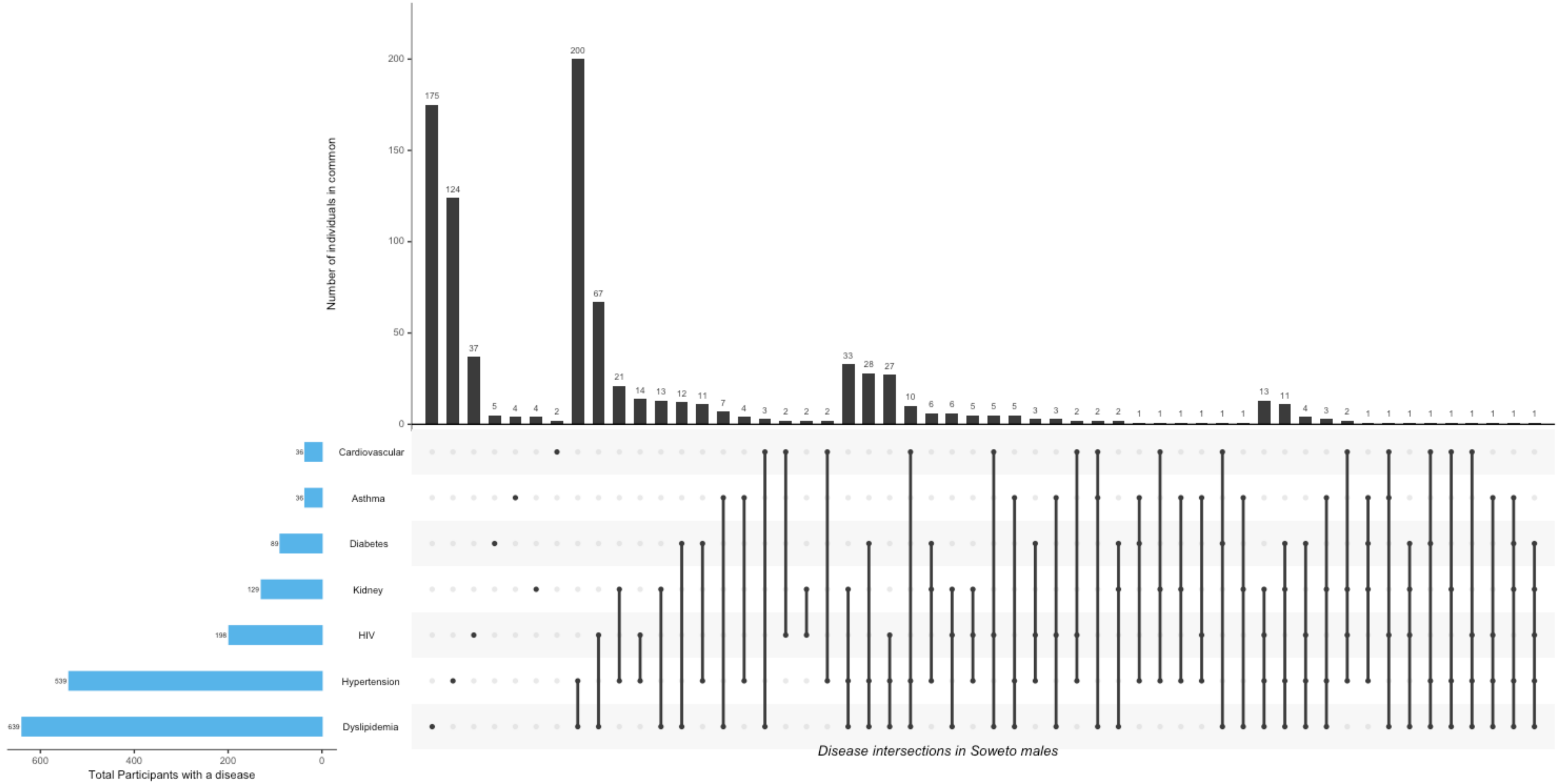

Supplementary figure 6

f

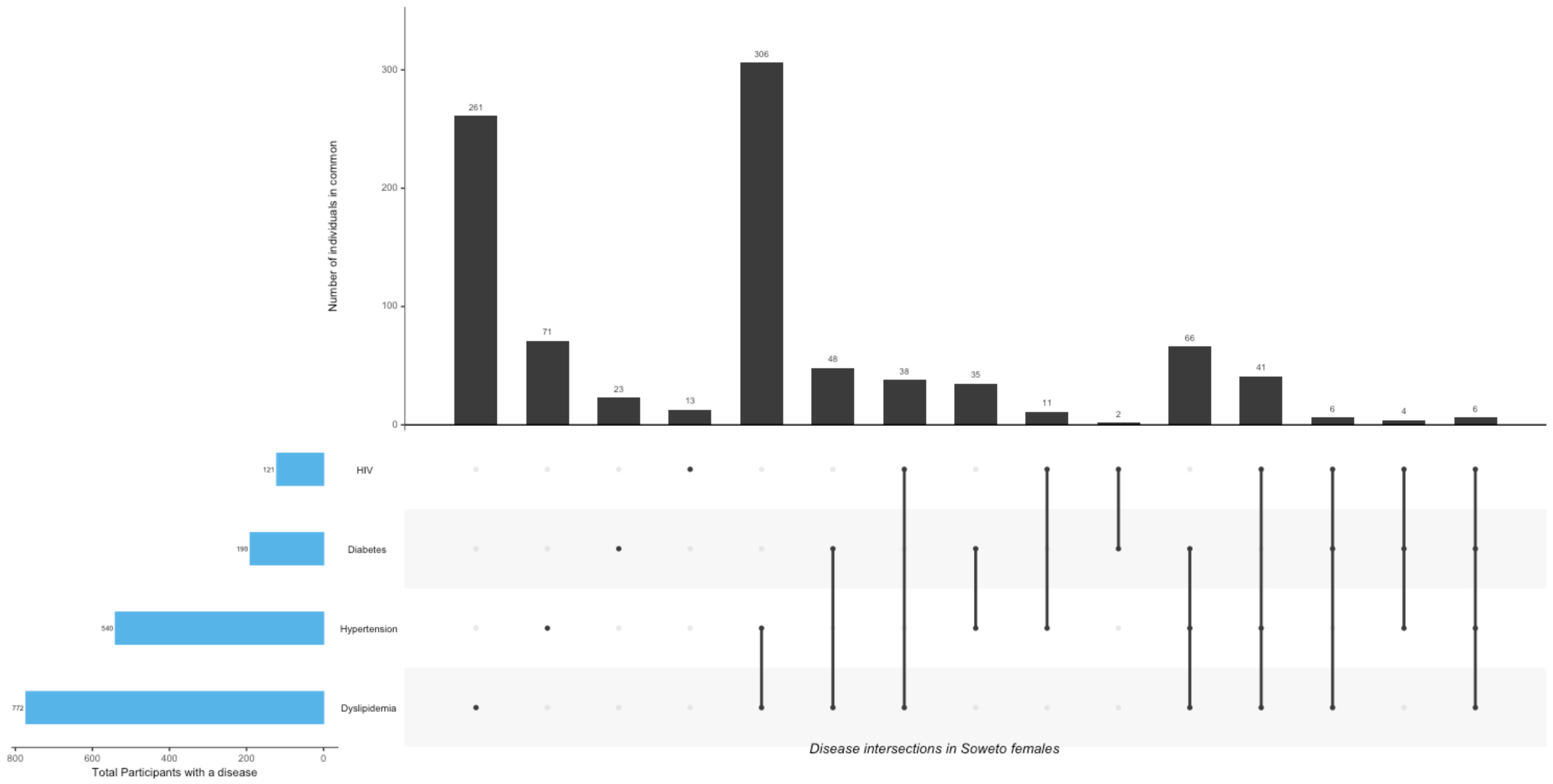

Supplementary figure 7

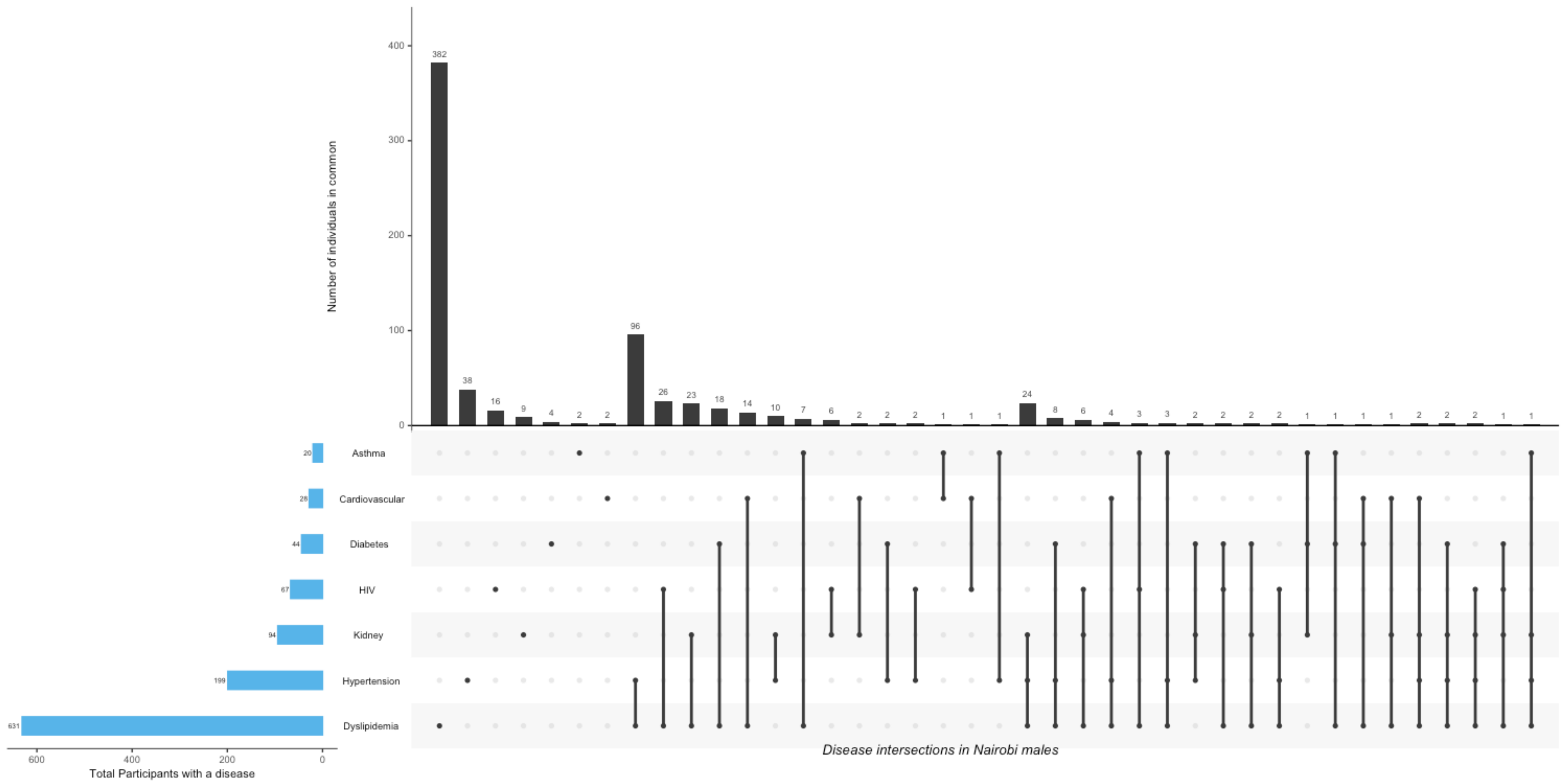

Supplementary figure 8

h

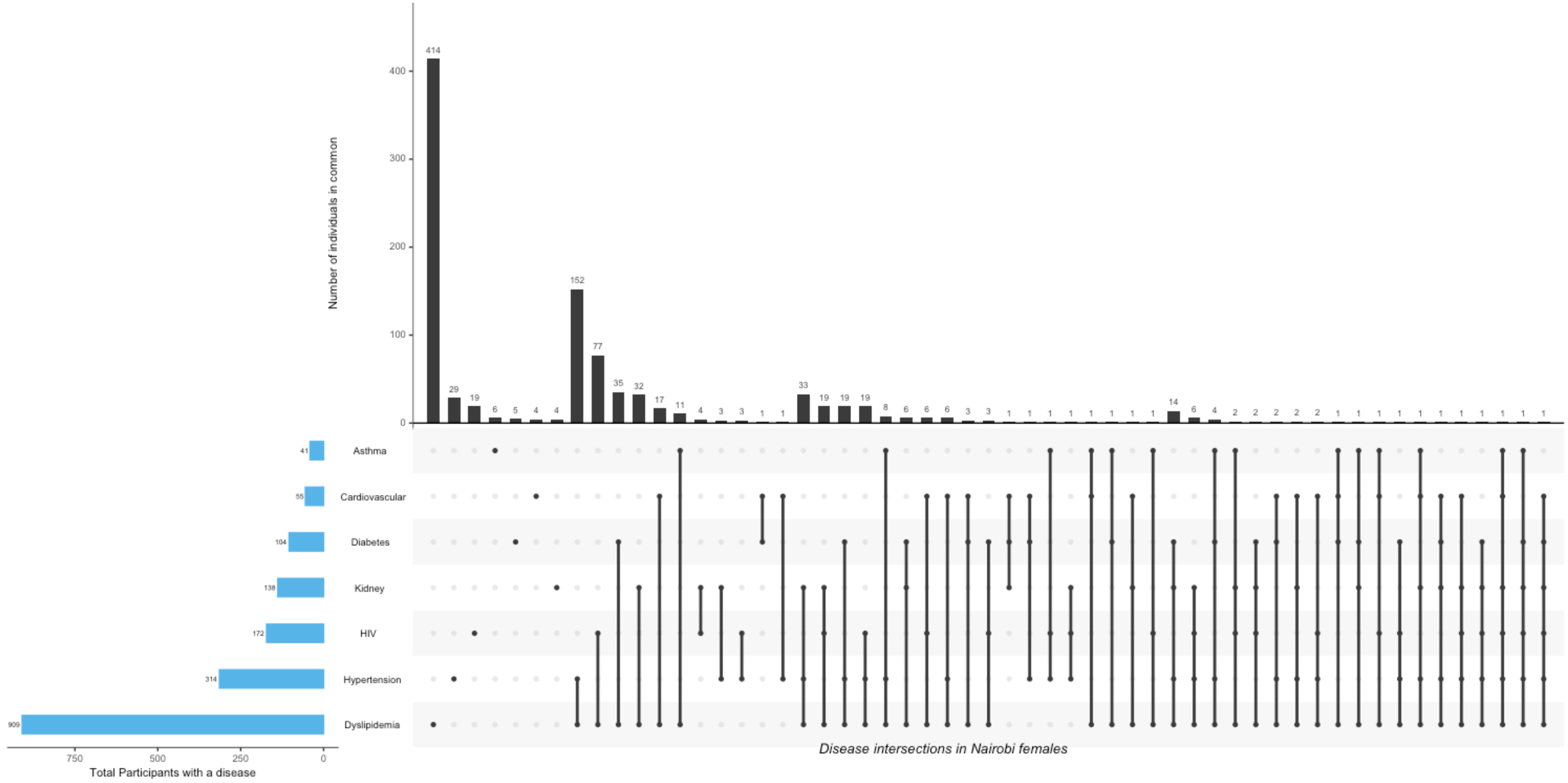

Supplementary figure 9 i

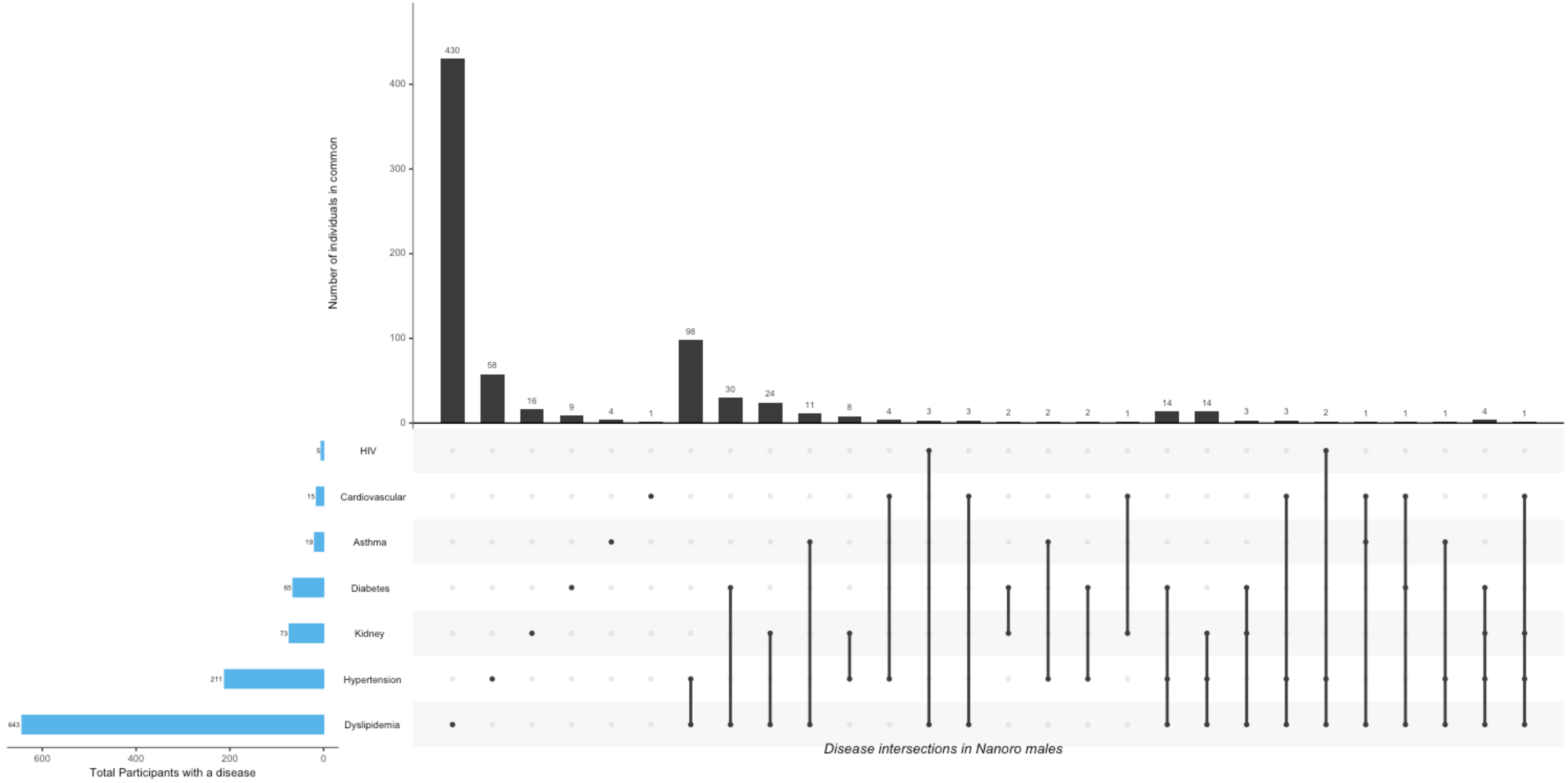

Supplementary figure 10

j

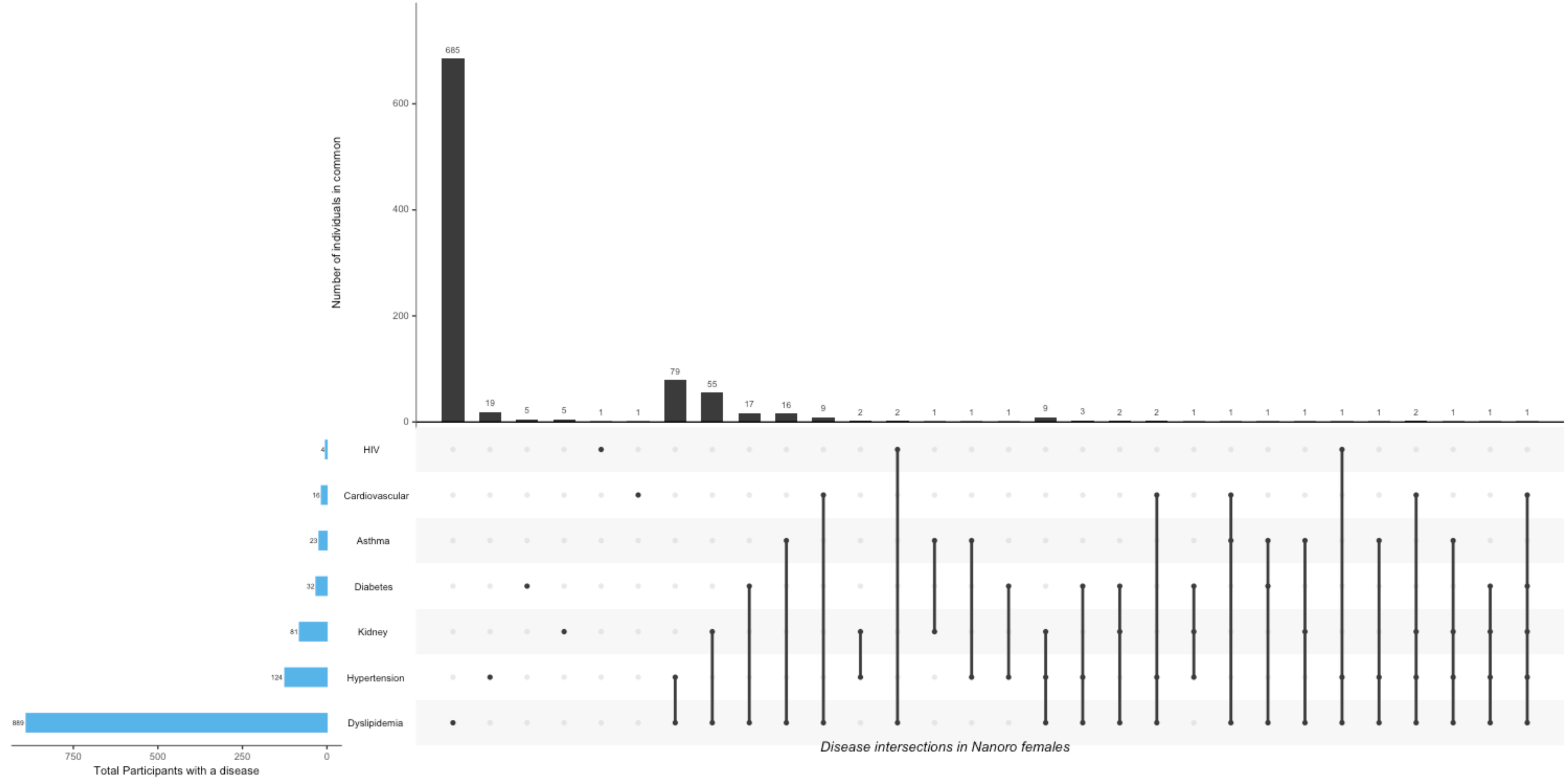

Supplementary figure 11

k

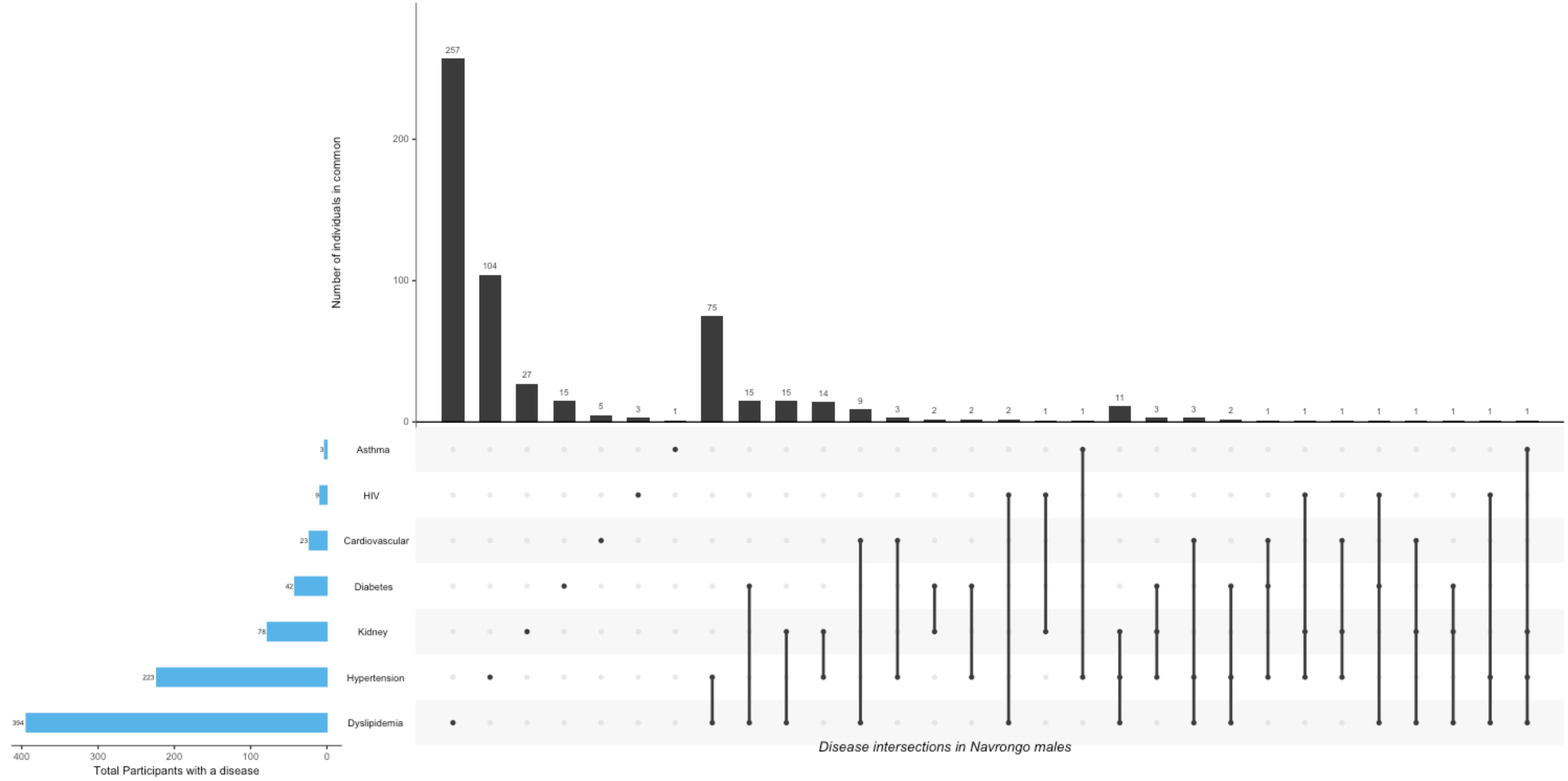

Supplementary figure 12

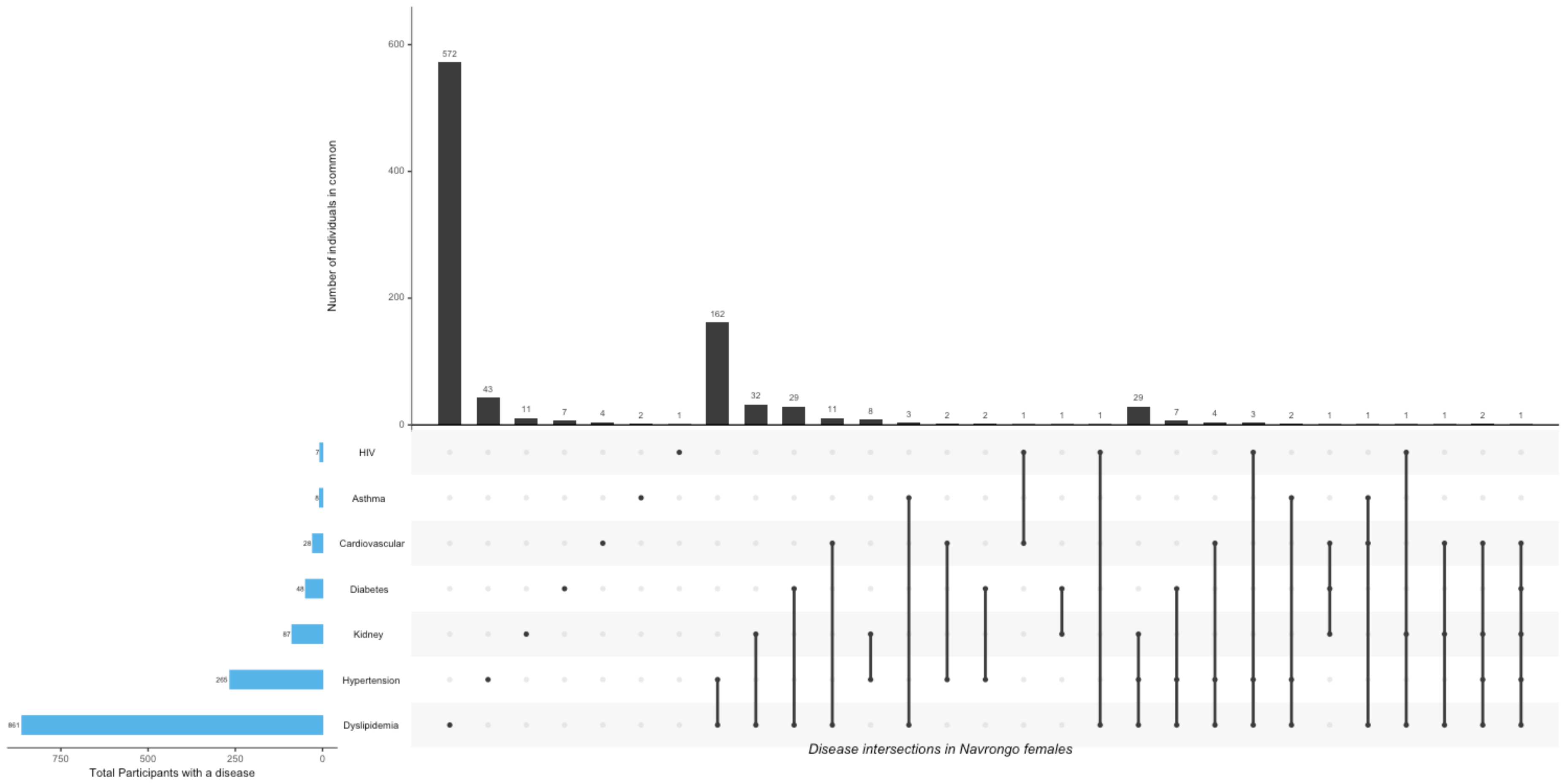

Reporting checklist for cross sectional study.

Based on the STROBE cross sectional guidelines.

Instructions to authors

Complete this checklist by entering the page numbers from your manuscript where readers will find each of the items listed below.

Your article may not currently address all the items on the checklist. Please modify your text to include the missing information. If you are certain that an item does not apply, please write "n/a" and provide a short explanation.

Upload your completed checklist as an extra file when you submit to a journal.

In your methods section, say that you used the STROBE cross sectionalreporting guidelines, and cite them as:

von Elm E, Altman DG, Egger M, Pocock SJ, Gotzsche PC, Vandenbroucke JP. The Strengthening the Reporting of Observational Studies in Epidemiology (STROBE) Statement: guidelines for reporting observational studies.

		Reporting Item	Page Number
**Title and abstract**			
Title	#1a	Indicate the study's design with a commonly used term in the title or the abstract	1
Abstract	#1b	Provide in the abstract an informative and balanced summary of what was done and what was found	3
**Introduction**			
Background / rationale	#2	Explain the scientific background and rationale for the investigation being reported	5
Objectives	#3	State specific objectives, including any prespecified hypotheses	6
**Methods**			
Study design	#4	Present key elements of study design early in the paper	7
Setting	#5	Describe the setting, locations, and relevant dates, including periods of	7

For peer review only

		recruitment, exposure, follow-up, and data collection	
Eligibility criteria	#6a	Give the eligibility criteria, and the sources and methods of selection of participants.	7
	#7	Clearly define all outcomes, exposures, predictors, potential confounders, and effect modifiers. Give diagnostic criteria, if applicable	7-9
Data sources / measurement	#8	For each variable of interest give sources of data and details of methods of assessment (measurement). Describe comparability of assessment methods if there is more than one group. Give information separately for for exposed and unexposed groups if applicable.	7-9
Bias	#9	Describe any efforts to address potential sources of bias	7
Study size	#10	Explain how the study size was arrived at	7
Quantitative variables	#11	Explain how quantitative variables were handled in the analyses. If applicable, describe which groupings were chosen, and why	9
Statistical methods	#12a	Describe all statistical methods, including those used to control for confounding	9
Statistical methods	#12b	Describe any methods used to examine subgroups and interactions	9
Statistical methods	#12c	Explain how missing data were addressed	9
Statistical methods	#12d	If applicable, describe analytical methods taking account of sampling strategy	9
Statistical methods	#12e	Describe any sensitivity analyses	n/a
**Results**			
Participants	#13a	Report numbers of individuals at each stage of study—eg numbers potentially eligible, examined for eligibility, confirmed eligible, included in the study, completing follow-up, and analysed. Give information separately for for exposed and unexposed groups if applicable.	10
Participants	#13b	Give reasons for non-participation at each stage	n/a
Participants	#13c	Consider use of a flow diagram	n/a

For peer review only

Descriptive data	#14a	Give characteristics of study participants (eg demographic, clinical, social) and information on exposures and potential confounders. Give information separately for exposed and unexposed groups if applicable.	10
Descriptive data	#14b	Indicate number of participants with missing data for each variable of interest	n/a
Outcome data	#15	Report numbers of outcome events or summary measures. Give information separately for exposed and unexposed groups if applicable.	10-11
Main results	#16a	Give unadjusted estimates and, if applicable, confounder-adjusted estimates and their precision (eg, 95% confidence interval). Make clear which confounders were adjusted for and why they were included	n/a
Main results	#16b	Report category boundaries when continuous variables were categorized	10-12
Main results	#16c	If relevant, consider translating estimates of relative risk into absolute risk for a meaningful time period	n/a
Other analyses	#17	Report other analyses done—e.g., analyses of subgroups and interactions, and sensitivity analyses	n/a
**Discussion**			
Key results	#18	Summarise key results with reference to study objectives	13
Limitations	#19	Discuss limitations of the study, taking into account sources of potential bias or imprecision. Discuss both direction and magnitude of any potential bias.	16
Interpretation	#20	Give a cautious overall interpretation considering objectives, limitations, multiplicity of analyses, results from similar studies, and other relevant evidence.	13-16
Generalisability	#21	Discuss the generalisability (external validity) of the study results	16
**Other Information**			
Funding	#22	Give the source of funding and the role of the funders for the present study and, if applicable, for the original study on which the present article is based	24

The STROBE checklist is distributed under the terms of the Creative Commons Attribution License CC-BY.

This checklist was completed on 23. August 2022 using https://www.goodreports.org/, a tool made by the EQUATOR Network in collaboration with Penelope.ai

For peer review only
